# STAMBP Accelerates Progression and Tamoxifen Resistance of Breast Cancer Through Deubiquitinating ERα

**DOI:** 10.3390/biom15111502

**Published:** 2025-10-24

**Authors:** Zhihuai Wang, Likai Gu, Mei Yang, Yi Zhou, Xihu Qin, Chen Xiong

**Affiliations:** 1 Department of General Surgery, The Affiliated Hospital of Guizhou Medical University, Guiyang 550001, China; wzh19940119@126.com; 2 Clinical Medical College, Guizhou Medical University, Guiyang 550001, China; 3 Changzhou Medical Center, Nanjing Medical University, Changzhou 213000, China; 18762313461@163.com (L.G.); victoryyangmei@163.com (M.Y.); ascis530@163.com (Y.Z.); 4 Clinical Medical College, Shandong University, Jinan 250012, China

**Keywords:** STAMBP, breast cancer, estrogen receptor α, endocrine therapy, deubiquitination

## Abstract

Breast cancer (BRCA) remains a global health burden, with endocrine-resistant ER-positive BRCA posing therapeutic challenges. This study investigates STAMBP’s role in breast cancer progression and evaluates its potential as a therapeutic target. Through siRNA library screening in ER-positive cell lines, we identified STAMBP as a key regulator of ERα signaling and observed its upregulation in BRCA samples. (fold changes > 2, sample sizes = 30, *p* < 0.001), particularly in ER-positive subtypes. Prognostic analysis demonstrated that STAMBP overexpression correlates with poor clinical outcomes in ER-positive BRCA patients (*p* < 0.05). In vitro functional assays showed STAMBP promoted proliferation, metastasis, and epithelial–mesenchymal transition of ER-positive cells by regulating the activity of ERα signaling. Mechanistically, the deubiquitinase STAMBP directly reduces the K48-linked polyubiquitination levels of ERα, enhancing its protein stability and activating downstream oncogenic signaling. STAMBP knockdown restored tamoxifen sensitivity in endocrine-resistant BRCA cells by reducing ERα stability. This study has certain limitations, including the absence of pharmacological validation and reliance on small, single-center clinical cohorts, which should be addressed in future research to further substantiate the clinical relevance of targeting STAMBP in BRCA. Collectively, our findings identified STAMBP as a prognostic marker and demonstrated its dual role in driving ER-positive BRCA malignancy and mediating endocrine resistance. Targeting STAMBP may represent an innovative approach to improve endocrine therapeutic efficacy in ER-positive BRCA.

## 1. Introduction

Breast cancer (BRCA) remains the leading cause of cancer-related mortality in women worldwide, responsible for approximately 685,000 deaths in 2020 [1]. Approximately 70% of breast tumors express estrogen receptor α (ERα), establishing endocrine therapy as a fundamental treatment strategy [2]. Current therapeutic approaches for BRCA include surgical intervention, chemotherapy, targeted therapy, and endocrine agents such as tamoxifen and aromatase inhibitors—all of which have significantly reduced BRCA-related mortality rates [3]. The ER functions as the primary transcriptional regulator in ~75% of breast cancer cases, making it the central target of endocrine therapies [4]. However, resistance to endocrine therapy persists as a major clinical challenge. The molecular mechanisms driving endocrine resistance are multifaceted, involving ERα mutations, crosstalk with growth factor signaling pathways, and metabolic reprogramming [5]. Despite progress in personalized medicine, the identification of novel biomarkers and therapeutic targets remains crucial to overcoming resistance and enhancing patient outcomes.

As a master regulator of proteostasis, the ubiquitin system governs protein degradation and dysregulation of key pathways implicated in tumorigenesis [6]. Deubiquitinases (DUBs), which catalyze the removal of Ub, serve as key regulators of all Ub-dependent protein degradation processes [7]. The human proteome encodes seven structurally and mechanistically distinct DUB families: (1) ubiquitin-specific proteases (USPs), (2) Machado–Joseph disease protein domain proteases (MJDs), (3) ubiquitin C-terminal hydrolases (UCHs), (4) monocyte chemotactic protein-induced proteins (MCPIPs), (5) ovarian tumor-related proteases (OTUs), (6) JAB1/MPN-domain-associated metalloisopeptidases (JAMMs), and (7) the motif-interacting with ubiquitin-containing novel DUB family (MINDY) [8,9]. Several DUBs have been identified as critical regulators in the initiation and progression of breast cancer. For instance, USP51, a member of USPs, was found to regulate the chemoresistance of triple-negative BRCA by deubiquitinating GRP78 [10]. Another DUB USP24 was also demonstrated to inhibit ferroptosis in triple-negative BRCA through stabilizing DHODH protein [11]. The oncoprotein HER2 was found to be stabilized through USP7 activity, providing a molecular mechanism for HER2-driven breast cancer progression [12]. Immune therapeutic responses in BRCA were also regulated by DUBs. The member of UCHs, UCHL1, could inhibit the ubiquitination of PD-L1 to influence the immunotherapeutic effects of BRCA [13]. In addition, OTUD3, a member of OTUs, could suppress the TGF-β signaling pathway by deubiquitinating SMAD7 to inhibit BRCA metastasis [14]. Targeting DUBs in breast cancer represents a potential therapeutic strategy, warranting further mechanistic exploration.

The deubiquitinating enzyme STAM binding protein (STAMBP), a JAMM family member, fine tunes protein stability and signaling cascades through targeted ubiquitin chain editing [15]. Dysregulated STAMBP expression induces oncogenic features through altered ubiquitin signaling of substrate proteins by directly binding with the C-terminal JAMM domain of STAMBP, which could facilitate cellular transformation and tumor development. STAMBP has been illustrated to regulate several cancers, including lung cancer [16], pancreatic cancer [17], and triple-negative BRCA [18], by the function of DUBs. However, while STAMBP’s oncogenic role is emerging, its function in ER-positive BRCA remains unexplored.

In this study, we identify STAMBP as an important regulatory DUB of the ER signaling pathway. Unlike previously reported ERα-stabilizing DUBs identified in MCF-7 cells, STAMBP was discovered using T47D cells—an alternative ER-positive cell model. Different deubiquitinases may regulate ubiquitination of the same substrate protein, so we focused on finding more novel regulatory DUBs of the ER signaling pathway, which might provide more potential therapeutic targets. The expression levels, prognostic significances, functional roles, and molecular mechanisms of STAMBP in ER-positive BRCA were explored. The C-terminal JAMM domain of STAMBP, which contains its catalytic subunit, may bind directly to ERα and function as a deubiquitinase to suppress ERα ubiquitination. Our study attempts to seek to address how STAMBP regulates ER-positive tumor progression and whether STAMBP inhibition can resensitize endocrine-resistant cells to tamoxifen. By combining clinical sample analysis, in vitro functional assays, and mechanistic studies, we strive to establish STAMBP as a novel oncogenic driver in BRCA. The findings may unveil STAMBP as both a prognostic indicator and a potential target for treatment of tamoxifen-resistant ER-positive BRCA.

## 2. Materials and Methods

### 2.1. Clinical Samples Acquisition

All clinical BRCA and para-tumor samples (within 2 cm of the surrounding tumor tissues) in this study were acquired from Changzhou Medical Center, Nanjing Medical University. Patients were diagnosed with ER-positive BRCA in the last 3 years. All patients have approved the collection and signed the informed consent forms.

### 2.2. Acquisition and Analysis of Online Data

The mRNA data and prognostic information of BRCA and para-tumor tissues were gained from GEO (Gene Expression Omnibus), TCGA (The Cancer Genome Atlas Program) databases, and the Kaplan–Meier (K-M) Plotter online website (https://www.kmplot.com/). Detailed information of BRCA patients in the K-M Plotter website was listed in a previous report [19], which contains data from more than 50 GEO and EGA (European Genome-phenome Archive) databases. The TCGA-BRCA dataset contains the mRNA expression data of tumor samples and clinicopathological information from 1054 patients with breast cancer. GSE45827 contains the mRNA expression data of tumor samples and para-tumor samples from 131 patients with breast cancer. The GSE21653 dataset contains the mRNA expression data of tumor samples and clinicopathological information from 260 patients with breast cancer. The GSE25066 dataset contains the mRNA expression data of tumor samples and clinicopathological information from 472 patients with breast cancer. The GSE61304 dataset contains the mRNA expression data of tumor samples and clinicopathological information from 29 patients with breast cancer. The GSE22219 dataset contains the mRNA expression and prognostic data from 216 patients with breast cancer. Differential expression of mRNA was compared by the “limma” package in R software. These data from the TCGA and GEO databases were normalized by “limma” and “impute” in R software. Patients were stratified into high and low STAMBP expression groups based on median expression levels. Prognostic K-M curves of patients in each group were plotted by “ggplot2”, “survival”, and “survminer” R packages or the K-M Plotter website.

### 2.3. Functional Enrichment Analyses

A total of 807 patients with ER-positive BRCA were divided into a low expression group (404 patients) or a high expression group (403 patients). Differentially expressed genes (DEGs) between different groups were identified via the “limma” package (logFC ≥ 1, *p* < 0.05). GO and KEGG analyses were performed through the DAVID database to find potential biological functions correlated with STAMBP in BRCA; the enriched terms were assumed by the FDR (false discovery rate) value (−log10(*p*-value)). GSEA analysis was performed via “clusterProfiler” and “enrichplot” packages in R software.

### 2.4. Cell Cultures

MCF-7 and T47D cell lines were acquired from Guizhou Medical University. All cell lines were maintained in high-glucose DMEM (Gibco, Grand Island, NY, USA) supplemented with 10% heat-inactivated FBS (Gibco, USA) and 1% streptomycin-penicillin (Yeasen, Shanghai, China), cultured at 37 °C in 5% CO_2_. Cells were passaged at 80–90% confluence using 0.25% trypsin-EDTA, with medium changes every 48 h and routine mycoplasma testing.

### 2.5. RNA Isolation and Quantitative Real-Time PCR (qRT-PCR)

Total RNA was extracted using Trizol reagent (Beyotime, Beijing, China), with cDNA synthesized from 1 μg RNA using HiScript II Q RT SuperMix (Vazyme, Nanjing, China). qPCR was performed in triplicate using SYBR Master Mix (Yeasen, China) on a Bio-Rad CFX96 equipment. Detailed primer sequences were listed in Table S1.

### 2.6. Western Blot (WB)

Following RIPA buffer lysis (Solarbio, Beijing, China) with protease/phosphatase inhibitors, protein concentrations were normalized by BCA assay (Beyotime, China). Samples (20–30 μg) were separated on 10% SDS-PAGE gels and transferred to 0.45 μm PVDF membranes. After 1 h blocking with 5% non-fat milk/TBST, membranes were incubated with primary antibodies (4 °C overnight) and HRP-conjugated secondaries (1:5000, 2 h RT). Protein bands were detected by ECL (Yeasen, China).

### 2.7. Immunohistochemical (IHC) Stain and Immunofluorescence (If)

All specimens, including tumor implants, were fixed in 4% neutral buffered formalin and paraffin embedded. Tissue sections (4 μm thickness) were deparaffinized and subjected to antigen retrieval. After quenching endogenous peroxidase activity and blocking non-specific proteins, sections were incubated overnight at 4 °C with primary antibodies. HRP-polymer conjugated secondary antibodies were applied for 1 h at 37 °C, followed by DAB chromogenic development for 3 min. The staining intensity and the percentage of positively stained cells were quantitatively assessed to derive a composite immunostaining score for each tissue specimen. A semi-quantitative immunohistochemical scoring system was utilized, incorporating both staining intensity (graded 0–3: absent, weak, moderate, strong) and the percentage of positive cells (0: <25%; 1: 25–50%; 2: 51–75%; 3: >75%). The final immunoreactivity score (0–9) was derived by multiplying these two values, ensuring a standardized and reproducible assessment of protein expression across tissue sections.

Cells were fixed with 4% PFA (20 min, RT), permeabilized with 0.2% Triton X-100 (15 min), and blocked with 5% BSA (1 h, 37 °C). Primary antibody incubation was performed overnight at 4 °C. After washing, cells were incubated with fluorescent secondaries for 1 h at RT. Nuclei were stained with DAPI (1 μg/mL, 5 min) before imaging. Images were photographed by a fluorescence microscope (Nikon, Tokyo, Japan).

### 2.8. Plasmids and Short Hairpin RNA Transfection

Full-length Flag-STAMBP plasmids, full-length ERα plasmids, MYC-ERα plasmids, Y537S site mutation plasmids, HA-Ub plasmids, HA-Ub-K48 plasmids, HA-Ub-K63 plasmids, Flag-STAMBP-D348A site mutation plasmids, control blank plasmids, or lentivirus based on short hairpin RNAs (shRNAs) and the corresponding control vector were acquired from Shanghai Genechem Company. All vectors have siRNA resistance. Corresponding control plasmids or shRNAs were also gained from this company. The MOI value of transfecting the lentivirus to MCF-7 and T46D cells is 2. After transfecting with the lentivirus, cells were selected using 2 μg/mL puromycin (Solarbio, Beijing, China). All cells were transfected with the lentivirus or plasmids by Lipofectamine 3000 reagent according to the manufacturer. Detailed shRNA sequences are listed in Table S2.

### 2.9. Co-Immunoprecipitation (Co-IP) and Poly-Ubiquitination Assay

Cells were lysed in RIPA buffer with protease inhibitors. Cell lysates were added with antibody for 12 h at 4 °C. Protein–antibody complexes were captured with protein A/G beads, washed extensively, and analyzed by WB, with all antibody incubations performed according to manufacturers’ recommended protocols. Proteasomal inhibition was achieved by 4 h treatment with 20 μM MG132. Lysates were subjected to immunoprecipitation using the antibody overnight at 4 °C. Immune complexes were precipitated with protein A/G beads and washed prior to elution. Ubiquitination of proteins was detected by WB. In the IP experiments conducted in this study, MG132 was added to inhibit the proteasome, thereby suppressing the activity of endogenous DUBs in the cell lysate. This step was implemented to prevent the continuous degradation of ubiquitin chains or ubiquitinated proteins by DUBs, which could otherwise lead to the underestimation or complete loss of target ubiquitination signals and the subsequent introduction of false-negative results. For sample processing in the polyubiquitination assay, lysis buffer containing a denaturant (1% SDS) was used to thoroughly disrupt non-covalent interactions between proteins, such as those involving non-specifically bound contaminating proteins or protein complexes. Notably, covalently linked ubiquitin chains (which mediate the ubiquitination modification of target proteins) remain unaffected by SDS; thus, only the ubiquitination modification of the target protein itself was preserved. Subsequent heating (denaturation at 95 °C for 5 min) was performed to further ensure complete protein denaturation, after which the target protein was captured using the corresponding IP antibody.

### 2.10. DUB Small Interfering RNA (siRNA) Library

A siRNA library targeting 96 human DUBs (Dharmacon, Lafayette, CO, USA) was used for high-throughput screening. Cells were reverse-transfected with the DUB siRNA library in 96-well plates. Cells in the control group were transfected with blank control siRNA. After 72 h, total RNA was isolated to perform qRT-PCR. An alteration in GREB1 gene expression greater than 50% was considered indicative of the regulatory effects of the DUB gene. We performed these experiments in this study with three independent replicates.

### 2.11. Protein Stability Assays

Lentivirus-transduced HCC cells were treated with 100 μM cycloheximide (CHX) for 0–24 h to assess protein half-life, with lysates collected at indicated time points for WB assays. In addition, cells were treated with 10 μM MG132 for 12 h prior to lysis in RIPA buffer containing protease inhibitors. Levels of proteins were detected by WB assays.

### 2.12. Cell Proliferative and Migrative Assays

The detailed procedures of Cell Counting Kit-8 (CCK-8) assays, colony formation experiments, 5-ethynyl-2′-deoxyuridine (EDU) assays, and Transwell assays were performed in a previous study [20].

### 2.13. Luciferase Activity Experiments

Luciferase activity reflecting ER signaling was measured with the Dual-Luciferase Reporter Assay Kit (Promega, Walldorf, Germany) following the manufacturer’s protocol. Cells were co-transfected with an estrogen response element (ERE)-driven firefly luciferase reporter plasmid and a Renilla luciferase control vector. At 24 h post-treatment, cells were lysed and luciferase activity was measured using the Dual-Luciferase Reporter Assay (Promega).

### 2.14. Animal Experiments

For the in vivo tumorigenicity assay, 5-week-old female BALB/c nude mice were used, and all mice were purchased from Vital River Company. On the day of receipt, slow-release 17β-estradiol pellets (0.72 mg/90 days, manufactured by Innovative Research of America, Sarasota, FL, USA) were subcutaneously implanted into each mouse. The mice were randomly divided into 5 experimental groups, with 6 mice in each group. Strict randomization was achieved using a random number table to ensure the impartiality of group allocation. Twenty-four hours after pellet implantation, a mixed suspension containing approximately 4 × 10^6^ MCF-7 cells and Matrigel was injected into the mammary fat pad of each mouse. Subsequently, tumor sizes were measured at fixed intervals once a week, and tumor volume was calculated using the following formula: tumor volume = width^2^ × length/2. For the tamoxifen treatment group, tamoxifen citrate powder was dissolved in corn oil to prepare a stock solution with a concentration of 5 mg/mL. Vortex oscillation was performed to aid dissolution, and a 37 °C water bath was used if necessary to ensure complete solubilization. During administration, each mouse received an intraperitoneal injection of 0.1 mL of the stock solution (corresponding to a dosage of 5 mg/kg/day). Drug administration was initiated when tumors reached a measurable size (50 mm^3^), following a schedule of 5 consecutive days of administration followed by a 2-day interval; this cycle was maintained for 3–5 weeks. All experimental procedures involving mice in this study were strictly conducted in accordance with the guidelines approved by the Ethics Committee of Changzhou Medical Center of Nanjing Medical University (No: 2023KY127-01). Strict humane endpoint criteria were established during the experiment, including the following: tumor volume exceeding 2000 mm^3^, tumor ulceration or necrosis, unexpected weight loss of more than 20%, or the presence of distress symptoms such as limited mobility and dyspnea. If any of these criteria were met, the animals were humanely euthanized immediately. Throughout the experiment, mice were housed in a specific pathogen-free (SPF) barrier environment, where temperature, humidity, and light cycle (12 h light/12 h dark) were strictly controlled. All mice had free access to standard laboratory feed and sterile drinking water.

### 2.15. Statistical Analysis

All statistical analysis was based on R 4.2.2 software or GraphPad Prism 4.0.3 software. All experiments were based on three biological repetitions; the error bars in the figures represent the standard error of the mean. Univariate and multivariate Cox regression analyses were conducted to assess the independent prognostic value of STAMBP in BRCA. Statistical analyses in this study utilized student’s *t*-test and Pearson correlation based on available data. *p* < 0.05 and FDR < 0.01 were considered statistically significant.

## 3. Results

### 3.1. STAMBP Was a Regulator of ER Signaling and Associated with Poor Prognosis in ER-Positive Breast Cancer

**Figure 1:**
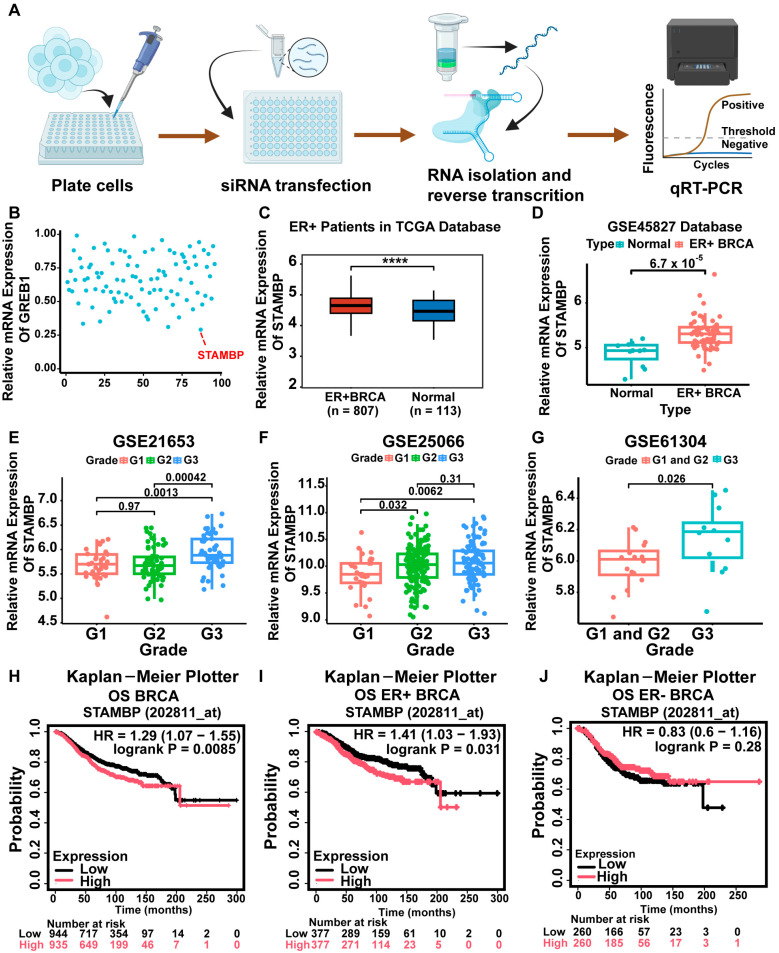
STAMBP was identified as a novel DUB regulating ERα signaling in ERα-positive BRCA. (**A**) The flow diagram of identifying DUBs that regulate ERα signaling through transfecting the siRNA library. Every DUB gene was inhibited in T47D cells transfected with 20 nM dissolved siRNAs. The gene expression levels were examined by qRT-PCR after 2 days. (**B**) The results of qRT-PCR showed the expression of the GREB1 gene in T47D cells transfected with siRNAs. STAMBP was identified as a pivotal DUB that influenced the expression of the GREB1 gene in T47D cells. (**C**) TCGA-BRCA data showed higher STAMBP mRNA levels in ERα-positive BRCA vs. adjacent normal tissues. (**D**) The GSE45827 dataset showed higher STAMBP mRNA levels in ERα-positive BRCA vs. adjacent normal tissues. (**E**–**G**) K-M curves plotted by the K-M plotter website show that the OS rates were dependent on STAMBP levels. High STAMBP expression predicts poor OS rates in ERα-positive BRCA but not in ERα-negative BRCA. (**H**–**J**) K-M curves plotted by the K-M plotter website show that the OS rates were dependent on STAMBP levels. High STAMBP expression predicts poor OS rates in ERα-positive BRCA but not in ERα-negative BRCA. **** *p* < 0.0001.

DUBs have been widely reported to play essential roles in the development of BRCA. To systematically identify novel regulators of ER signaling, we performed a genome-wide siRNA screen using a DUB-focused sub-library in ERα-positive T47D BRCA cells. (Figure 1A). ER signaling output was monitored via GREB1 mRNA quantification (qRT-PCR), a canonical ER target gene. siRNA library screening demonstrated that STAMBP knockdown reduced GREB1 transcriptional levels by more than 50%, identifying STAMBP as a novel DUB regulating ER signaling in ER-positive BRCA. (Figure 1B). Detailed DUB genes and corresponding GREB1 levels were listed in Table S4. Analysis of TCGA and GSE45827 datasets revealed consistent upregulation of STAMBP mRNA expression in ER-positive BRCA tissues compared to adjacent normal tissues. (Figure 1C,D). Furthermore, analysis of three GEO datasets (GSE21653, GSE25066, and GSE61304) demonstrated that elevated STAMBP expression correlates with advanced histopathological grades in ER-positive BRCA. (Figure 1E–G, *p* < 0.05), which resembles a possible association between STAMBP and the progression of ER-positive BRCA.

Univariate and multivariate Cox analyses showed that STAMBP was an independent prognostic factor for BRCA patients according to the clinicopathological information from the TCGA-BRCA dataset (Table S5, *p* < 0.05). To further assess STAMBP’s prognostic significance in BRCA, we analyzed survival data from the K-M Plotter database and GSE22219 dataset, evaluating the correlation between STAMBP expression levels and clinical outcomes. Corresponding K-M curves were plotted. The high expression of STAMBP indicated poor overall (OS) rate (Figure 1H, *p* < 0.05), recurrence-free survival (RFS) rate (Figure S1A, *p* < 0.05), distant metastasis-free survival (DMFS) rate (Figure S1D, *p* < 0.05), and disease-free survival (DFS) rate (Figure S1G, *p* < 0.05) of total patients with BRCA. Patients were subsequently stratified into ERα-positive and ERα-negative subgroups. Unexpectedly, elevated STAMBP was correlated with shorter OS (Figure 1I, *p* < 0.05), RFS (Figure S1B, *p* < 0.05), DMFS (Figure S1E, *p* < 0.05), and DFS (Figure S1H, *p* < 0.05) rates of ER-positive BRCA patients, but the expression level of STAMBP was not correlated with survival of ER-negative BRCA patients (Figure 1J and Figure S1C,F,I, *p* > 0.05). These results indicated that the influence of STAMBP on the survival of BRCA patients might depend on the status of ERα protein.

### 3.2. STAMBP Expression Upregulates and Influences Proliferation and Metastasis of ER-Positive BRCA In Vitro

**Figure 2:**
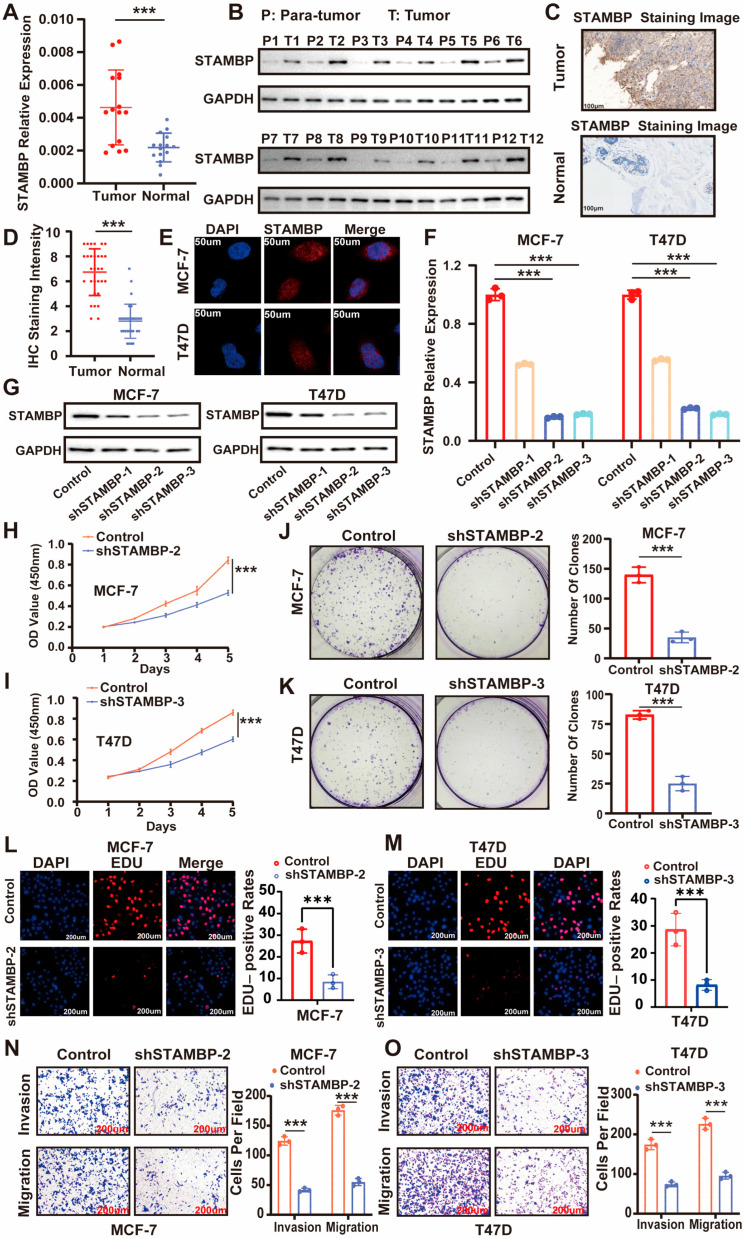
STAMBP accelerates the proliferation and metastasis of ERα-positive BRCA cells. (**A**) qRT-PCR showed higher STAMBP mRNA levels in tumor and adjacent normal tissues from 15 patients with ERα-positive BRCA. (**B**) Western blot showed STAMBP expression in tumor and adjacent normal (Para-tumor) tissues from 12 patients with ERα-positive BRCA. (**C**,**D**) Immunohistochemical staining showed protein expression of STAMBP in ERα-positive BRCA tissues and adjacent normal tissues from 30 patients (scale bar: 100 μm). (**E**) Immunofluorescence staining showed the location of STAMBP (red) in MCF-7 and T47D cells, with DAPI (blue) for nuclei (scale bar: 50 μm), confirming cytoplasmic and nuclear localization of STAMBP. (**F**,**G**) Efficiency of STAMBP shRNAs (shSTAMBP-2, shSTAMBP-3) verified by qRT-PCR (**F**) and Western blot (**G**) in MCF-7 cells. Cells were transfected with lentiviral vectors encoding shRNA or shControl. (**H**–**M**) Proliferation assays: CCK-8 (**H**,**I**), colony formation (**J**,**K**), and EDU staining (**L**,**M**) showed reduced viability and clonogenicity in shSTAMBP-transfected MCF-7 and T47D cells (scale bar: 200 μm). (**N**,**O**) Transwell assays revealed decreased migration and invasion in STAMBP-depletion cells (scale bar: 200 μm). *** *p* < 0.001. Original western blots can be found at Supplementary Materials.

We utilized clinical samples from patients diagnosed with ER-positive BRCA to validate the expression levels of STAMBP by experiments. qRT-PCR analysis revealed higher STAMBP mRNA levels in tumor tissues compared to adjacent normal tissues from 15 patients with ERα-positive breast cancer (Figure 2A). Western blot assays confirmed STAMBP expression in tumor and paired adjacent non-tumor (para-tumor) tissues from 12 patients with ERα-positive breast cancer (Figure 2B). Consistent with these findings, IHC staining demonstrated STAMBP expression in ERα-positive breast cancer tissues and their matched adjacent normal tissues from 30 patients (scale bar: 100 μm; Figure 2C,D). Collectively, these results suggest that STAMBP may be associated with the progression of ERα-positive BRCA.

The ER-positive cell lines MCF-7 and T47D were utilized for further experimental validation. The intracellular location of STAMBP in MCF-7 and T47D was explored by IF staining (Figure 2E). Specific shRNAs were designed and implemented to effectively knock down STAMBP expression in cellular models. The efficiencies for inhibiting STAMBP expression by shRNAs were validated through qRT-PCR (Figure 2F, *p* < 0.05) and WB assays (Figure 2G). The results of CCK-8 assays (Figure 2H,I, *p* < 0.05), clone formation assays (Figure 2J,K, *p* < 0.05), and EDU assays (Figure 2L,M, *p* < 0.05) showed that the proliferative capacities of MCF-7 and T47D cells were inhibited when the expression of STAMBP was decreased through shRNAs. Transwell migration and invasion assays demonstrated that STAMBP inhibition significantly suppressed cellular migratory and invasive capabilities (Figure 2N,O, *p* < 0.05). These findings indicated a positive association between STAMBP and the progression of ER-positive BRCA.

### 3.3. STAMBP Influences Epithelial–Mesenchymal Transition (EMT) and ER Signaling in ER-Positive BRCA Cells

**Figure 3:**
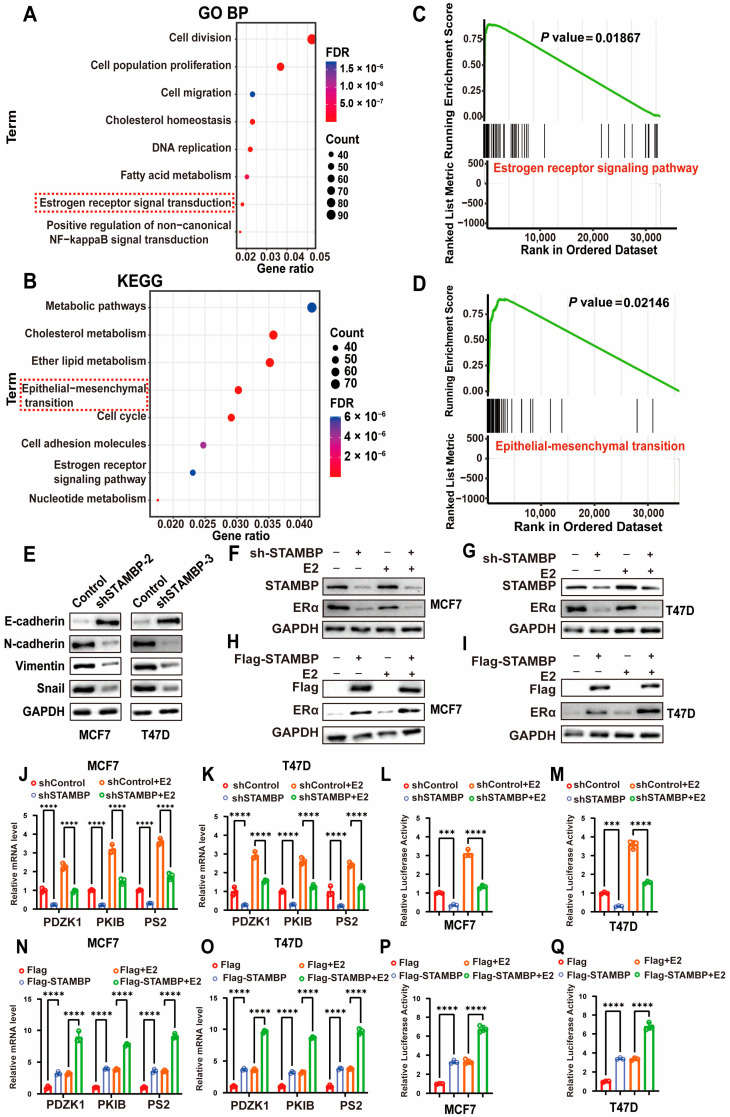
STAMBP is associated with the activity of ERα signaling in BRCA. (**A**,**B**) The mRNA data of patients with ERα-positive BRCA were divided into 2 different groups according to the STAMBP expression levels. DEGs between the high-expression group and the low-expression group were identified. GO (**A**) and KEGG (**B**) analyses were performed to explore potential biological mechanisms correlated with STAMBP according to these DEGs. (**C**,**D**) GSEA analysis based on DEGs showed the expression of STAMBP in Erα-positive BRCA was correlated with the ER signaling pathway (**C**) and EMT (**D**). (**E**) Western blot exhibited EMT markers levels in MCF-7 and T47D cells with STAMBP knockdown. (**F**–**I**) The protein levels of STAMBP and ERα in ERα-positive BRCA cells were examined by WB. BRCA cells were transfected with lentiviral vectors encoding shRNA or shControl and then added with the control vector or 10 nM estradiol for 6 h (**F**,**G**). BRCA cells were transfected with Flag-STAMBP overexpression plasmid and then added with the control vector or 10 nM estradiol for 6 h (**H**,**I**). (**J**,**K**,**N**,**O**) The expression levels of three downstream genes of ER signaling were determined through qRT-PCR in ERα-positive BRCA cells. BRCA cells were transfected with lentiviral vectors encoding shRNA or shControl and then added with control vector or 10 nM estradiol for 6 h. (**L**,**M**,**P**,**Q**) The activities of ERE luciferases were examined through luciferase experiments in ERα-positive BRCA cells. BRCA cells were transfected with lentiviral vectors encoding shRNA or shControl and then added with control vector or 10 nM estradiol for 6 h. *** *p* < 0.001, **** *p* < 0.0001. Original western blots can be found at Supplementary Materials.

To investigate STAMBP’s functional roles in breast cancer, we conducted GO and KEGG pathway enrichment analyses using TCGA data, identifying key biological processes and signaling pathways linked to STAMBP expression. GO biological process analysis revealed STAMBP’s association with cell division, population proliferation, migration, cholesterol homeostasis, fatty acid metabolism, and estrogen receptor signaling pathways (Figure 3A). KEGG pathway analysis similarly indicated STAMBP’s involvement in metabolic pathways, cholesterol metabolism, epithelial–mesenchymal transition, and estrogen receptor signaling (Figure 3B). Additionally, GSEA enrichment analyses further supported STAMBP’s potential association with estrogen receptor signaling and EMT (Figure 3C,D, *p* < 0.05). These results all indicated that STAMBP might regulate the ERα signaling pathway and EMT.

WB assays demonstrated that the protein level of E-cadherin was significantly increased, and the protein levels of N-cadherin, snail, and vimentin were obviously decreased when inhibiting STAMBP expression (Figure 3E). The results of qRT-PCR also exhibited similar changes (Figure S2A,B). IF staining, for the expression of E-cadherin, was significantly increased when knocking down the STAMBP in BRCA cells (Figure S2C,D). In addition, the cellular shapes were also changed (Figure S2E,F). STAMBP-depleted cells exhibited a compact, cobblestone-like epithelial morphology, whereas control lentivirus-transfected cells displayed an elongated, spindle-shaped fibroblastic appearance. All results showed that inhibiting STAMBP expression could significantly suppress the EMT of MCF-7 and T47D cells. These findings validated that STAMBP could regulate the EMT and ERα signaling pathway in ER-positive BRCA cells. STAMBP knockdown in MCF-7 and T47D cells significantly decreased ERα abundance under both basal (vector) and estradiol-stimulated conditions (Figure 3F,G). Conversely, STAMBP overexpression elevated ERα protein levels in both treatment contexts, suggesting a regulatory role in ERα protein stability (Figure 3H,I). The concordant downregulation of PDZK1, PKIB, and PS2 upon STAMBP depletion (Figure 3J,K, *p* < 0.05)—along with their upregulation upon STAMBP overexpression (Figure 3N,O, *p* < 0.05)—supports a functional role for STAMBP in potentiating ERα transcriptional activity, potentially through stabilizing ERα or enhancing its coactivator recruitment. In addition, the ERE luciferase assay was utilized to assess the transcriptional activity of ERα. Similar results were shown in ERE luciferase assays when inhibiting (Figure 3L,M, *p* < 0.05) or overexpressing (Figure 3P,Q, *p* < 0.05) STAMBP expression in ER-positive BRCA cells that were treated with vector or estradiol.

### 3.4. STAMBP Accelerates the Progression of BRCA Through ERα

**Figure 4:**
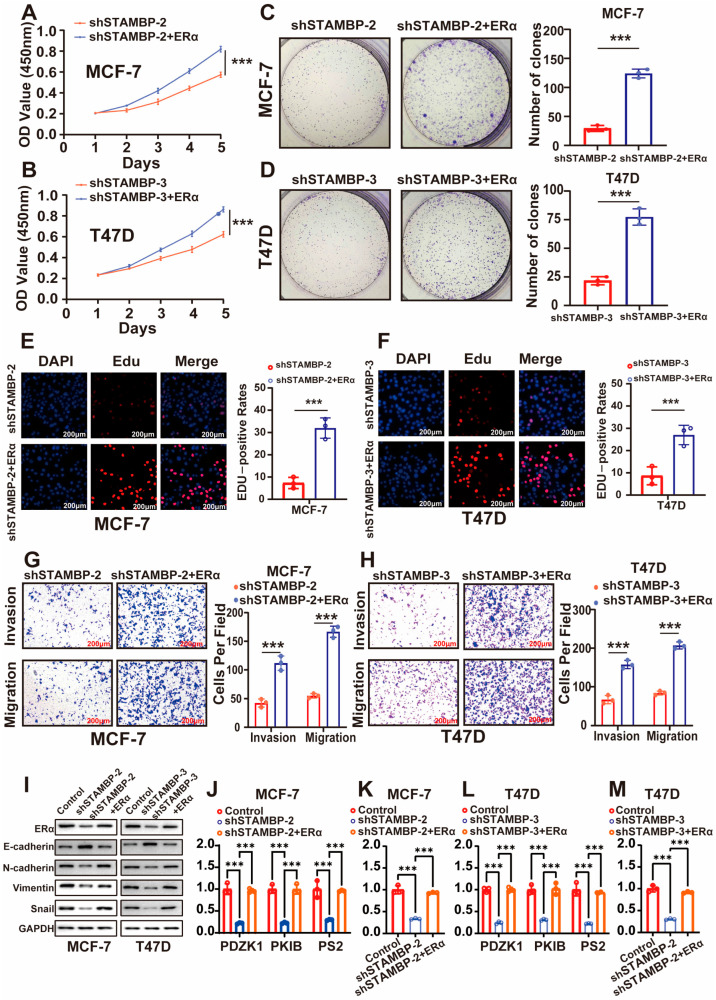
STAMBP accelerates BRCA progression through ERα-mediated signaling. (**A**,**B**) CCK-8 assays showed ERα overexpression reversed the proliferative defect induced by shSTAMBP. BRCA cells with STAMBP knockdown were transfected with either control plasmid vectors or a full-length ERα expression plasmid. (**C**,**D**) Colony formation assays confirmed restored clonogenicity with ERα rescue. BRCA cells with STAMBP knockdown were transfected with either control plasmid vectors or a full-length ERα expression plasmid. (**E**,**F**) EDU assays exhibited ERα overexpression reversed the proliferative defect induced by shSTAMBP. BRCA cells with STAMBP knockdown were transfected with either control plasmid vectors or a full-length ERα expression plasmid (scale bar: 200 μm). (**G**,**H**) Transwell assays revealed that ERα overexpression rescued the metastatic defect in shSTAMBP cells (scale bar: 200 μm). BRCA cells with STAMBP knockdown were transfected with either control plasmid vectors or a full-length ERα expression plasmid. (**I**) Western blot showed ERα overexpression reversed EMT inhibition by shSTAMBP. BRCA cells with STAMBP knockdown were transfected with either control plasmid vectors or a full-length ERα expression plasmid. After 48 h, total proteins were isolated. (**J**,**L**) The expression levels of three downstream genes of ER signaling were restored after overexpressing ERα in BRCA cells with STAMBP inhibition. BRCA cells were transfected with lentiviral vectors encoding shRNA or shControl, or a full-length ERα expression plasmid. (**K**,**M**) The activity of ERE luciferases was reversed after overexpressing ERα in BRCA cells with STAMBP inhibition. BRCA cells were transfected with lentiviral vectors encoding shRNA or shControl, or a full-length ERα expression plasmid. *** *p* < 0.001. Original western blots can be found at Supplementary Materials.

Our findings suggest a potential functional association between STAMBP and ERα signaling in ER-positive BRCA. Based on the observed STAMBP-dependent regulation of ERα downstream transcriptional activity, we proposed that STAMBP regulates the malignant behaviors of ER-positive BRCA cells via regulating ERα. The rescue experiments were utilized to validate our guess. CCK-8 assays (Figure 4A,B, *p* < 0.05), clone formation assays (Figure 4C,D, *p* < 0.05), EDU assays (Figure 4E,F, *p* < 0.05), and Transwell assays (Figure 4G,H, *p* < 0.05) demonstrated that the inhibition of proliferative, invasive, and migrative capacities of BRCA cells was obviously reversed by the overexpression of ERα. Additionally, the regulation of EMT phenotype by STAMBP inhibition was also reversed through the overexpression of ERα (Figure 4I). Similar results were shown in the results of ERα signaling correlated experiments. To overexpress the ERα in BRCA with STAMBP inhibition also obviously increased the expression of ERα downstream genes (Figure 4J,L, *p* < 0.05) and the transcriptional activity of ERα (Figure 4K,M, *p* < 0.05). These results all demonstrated that STAMBP regulated the proliferation, metastasis, EMT, and the activity of ERα signaling through ERα.

### 3.5. STAMBP Promotes ERα Protein Stability Through Inhibiting K48-Linking Poly-Ubiquitination

**Figure 5:**
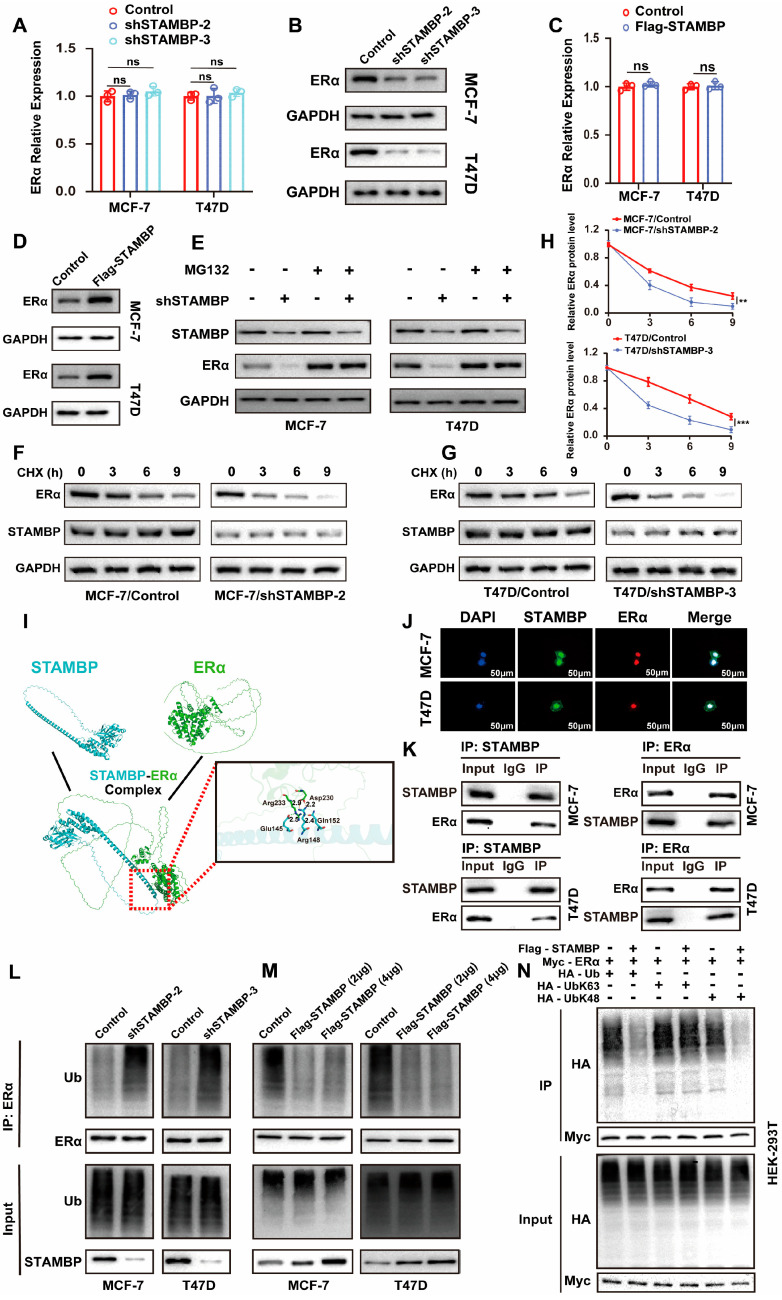
STAMBP deubiquitinates ERα protein through suppressing K48-linking poly-ubiquitination of ERα. (**A**,**C**) qRT-PCR analysis of ERα mRNA levels in MCF-7 and T47D cells with STAMBP knockdown (**A**) or overexpression (**C**) demonstrated unchanged ERα transcription. BRCA cells were transfected with lentiviral vectors encoding shRNA or shControl. (**B**,**D**) Western blot assays revealed altered ERα protein levels upon STAMBP knockdown (**B**) or overexpression (**D**) in MCF-7 and T47D cells. BRCA cells were transfected with lentiviral vectors encoding shRNA or shControl. (**E**) Treatment with proteasome inhibitor MG132 in BRCA cells rescued ERα protein reduction induced by STAMBP knockdown, suggesting STAMBP regulation via ubiquitin–proteasome pathway. Cells were transfected with lentiviral vectors encoding shRNA or shControl. Following a 24 h transfection period, cells were exposed to 10 μM MG132 for 6 h to inhibit proteasomal activity. (**F**–**H**) CHX chase assay showed decreased ERα protein half-life in STAMBP-knockdown cells. Cells were transfected with lentiviral vectors encoding shRNA or shControl. Cells were incubated with 100 μM cycloheximide for specified durations to inhibit protein synthesis. ERα protein expression levels were quantified using ImageJ 1.8.0 software. (**I**) Molecular docking prediction of ERα protein and STAMBP. (**J**) IF staining showed the colocalization of STAMBP and ERα in ERα-positive BRCA cells (scale bar: 50 μm). (**K**) WB and IP assays showed the combination of STAMBP and ERα protein in ERα-positive BRCA cells. (**L**,**M**) IP and WB assays revealed increased ERα polyubiquitination upon STAMBP knockdown (**L**) and decreased polyubiquitination upon STAMBP overexpression (**M**) in MCF-7 and T47D cells. Cells were transfected with shSTAMBP or either 2 μg or 4 μg of full-length Flag-STAMBP expression plasmid or corresponding control vectors. Twenty-four hours post-transfection, cells were treated with 10 μM MG132 for 6 h. Co-IP assays were subsequently conducted to assess ubiquitination levels of ERα. (**N**) K48 or K63 special polyubiquitination of ERα in HEK-293T cells was examined through WB and IP assays after transfecting with corresponding plasmids. Cells were transfected with full-length Flag-STAMBP, Myc-ERα, HA-Ub, HA-Ub-K48-only, or HA-Ub-K63-only plasmids. Twenty-four hours post-transfection, cells were treated with 10 μM MG132 for 6 h. Co-IP assays were subsequently conducted to assess ubiquitination levels of ERα. ns = not significant, ** *p* < 0.01, *** *p* < 0.001. Original western blots can be found at Supplementary Materials.

The relationship between STAMBP and ERα was investigated in ER-positive BRCA cells. The mRNA levels of ERα were not altered when the expression of STAMBP was suppressed or promoted in BRCA cells (Figure 5A,C, *p* > 0.05). These results all showed that STAMBP could not regulate ERα expression via influencing the transcription of ERα. However, WB assays exhibited that altering STAMBP expression in BRCA cells significantly changed the protein levels of ERα (Figure 5B,D). Given that protein stability is predominantly regulated by the ubiquitin–proteasome system, we investigated whether STAMBP modulates ERα stability through regulating its ubiquitination status. Treatment with the proteasome inhibitor MG132 prevented the STAMBP knockdown-induced decrease in ERα protein levels, suggesting STAMBP regulates ERα stability through the ubiquitin-proteasome system (Figure 5E). In addition, CHX assays further demonstrated that STAMBP depletion reduces ERα protein half-life in BRCA cells (Figure 5F–H). Molecular docking prediction showed a possible protein combination mode of STAMBP and ERα (Figure 5I). The IF staining showed the colocalization of STAMBP and ERα, which further validates a possible combination of STAMBP and ERα (Figure 5J). An endogenous IP assay demonstrated that STAMBP could interact with ERα in ER-positive BRCA cells (Figure 5K). The levels of ubiquitin in ERα protein were examined by IP assays and WB assays. We found that inhibiting or overexpressing STAMBP in BRCA cells could significantly increase or reduce the polyubiquitination levels of ERα (Figure 5L,M). K48- and K63-linked polyubiquitination represent the most prevalent ubiquitin chain types. We aimed to determine the specific ubiquitin linkage type through which STAMBP regulates ERα. Transfection with K48- or K63-specific ubiquitin plasmids in HEK-293T cells revealed that STAMBP knockdown reduced total polyubiquitination and specifically decreased K48-linked ubiquitination of ERα, without affecting K63-linked chains (Figure 5N). These findings demonstrate that STAMBP stabilizes ERα by selectively inhibiting its K48-linked polyubiquitination. Overexpression of the catalytically inactive STAMBP mutant (D348A) failed to reduce the total polyubiquitination level of ERα (Figure S3A,B), indicating that STAMBP-mediated deubiquitination of ERα is dependent on its intrinsic deubiquitinase activity.

### 3.6. Suppressing STAMBP Expression Sensitize Endocrine-Resistance BRCA to Tamoxifen Therapy

**Figure 6:**
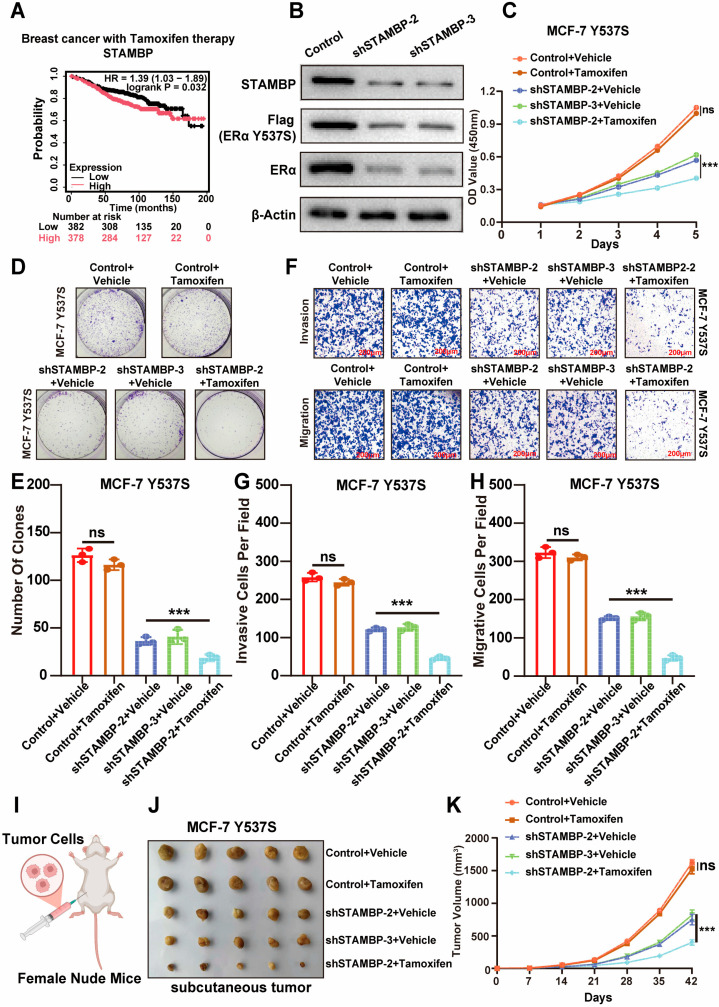
Suppression of STAMBP recovered tamoxifen sensitivity in endocrine-resistant BRCA. (**A**) K-M analysis of RFS rates in ERα-positive BRCA patients treated with tamoxifen from K-M Plotter online database, showing worse prognosis in patients with high STAMBP expression. (**B**) WB assays revealed wild-type and mutant (Y537S) ERα protein levels in endocrine-resistant MCF-7 cells with STAMBP knockdown. (**C**–**E**) CCK-8 (**C**) and colony formation (**D**,**E**) assays demonstrated that inhibiting STAMBP could suppress proliferative ability of MCF-7 Y537S cells. Cells were transfected with shControl or shSTAMBP for 48 h. Then, cells were added with 1μM Tamoxifen or control vehicle for 24 h. N = 3. (**F**–**H**) Transwell assays showed reduced invasive (**G**) and migrative (**H**) ability upon STAMBP inhibition in MCF-7 Y537S cells (scale bar: 200 μm). Cells were transfected with shControl or shSTAMBP for 48 h. Then, cells were added with 1μM Tamoxifen or control vehicle for 24 h. N = 3. (**I**) Schematic of xenograft model establishment using endocrine-resistant MCF-7 (ERα Y537S) cells in nude mice. (**J**,**K**) STABMP inhibition could suppress tumor growth and improve tamoxifen effects in MCF-7 Y537S cells by a xenograft model. N = 5. Tumor pictures (**J**) and tumor growth curves (**K**) showed restoration of tamoxifen efficacy in STAMBP-knockdown xenografts. ns = not significant, *** *p* < 0.001. Original western blots can be found at Supplementary Materials.

Tamoxifen is a common therapeutic drug to treat ER-positive BRCA by targeting and inhibiting ERα. Notably, according to the data from the K-M Plotter online website, high STAMBP expression was significantly associated with worse RFS rates in ER-positive BRCA patients receiving long-term tamoxifen therapy (Figure 6A, *p* < 0.05), which indicated that STAMBP might be correlated with tamoxifen resistance in ER-positive breast cancer. The MCF-7 cell line with mutant ERα (Y537S) was constructed to build an endocrine resistance model [21]. This model was utilized to explore the influences of STAMBP on ERα signaling and malignant behaviors in BRCA with endocrine resistance. WB assays demonstrated that knocking down the STAMBP expression could reduce the mutant type and wild type of ERα proteins (Figure 6B). Knocking down STAMBP expression and treating with tamoxifen also obviously decreased the expression of ERα downstream genes (Figure S4A, *p* < 0.05) and the transcriptional activity of ERα (Figure S4B, *p* < 0.05) in BRCA cells with resistance to tamoxifen therapy. CCK-8 assays (Figure 6C, *p* < 0.05), clone formation assays (Figure 6D,E, *p* < 0.05), and transwell assays (Figure 6F–H, *p* < 0.05) showed that inhibiting STAMBP expression could obviously sensitize BRCA cells with endocrine resistance to tamoxifen therapy. MCF-7 cells with mutant ERα (Y537S) were injected into the fat pad of nude mice to build an endocrine resistance xenograft tumor model (Figure 6I). We found that the suppression of STAMBP expression could also restore the therapeutic effects of tamoxifen in the xenograft tumor model (Figure 6J,K, *p* < 0.05). According to these findings, targeting STAMBP expression might be an effective strategy for destroying tamoxifen resistance caused by the mutation of ERα.

### 3.7. Entrectinib Inhibits STAMBP Expression and Suppresses Tumor Progression in BRCA Cells

A previous study identified entrectinib as a special inhibitor of STAMBP to reduce the protein levels of STAMBP [17]. They found an anti-tumor role of entrectinib in pancreatic cancer. We aimed to explore the effects of entrectinib in ER-positive BRCA. Treating with entrectinib could obviously inhibit the expression of STAMBP in ER-positive BRCA cells (Figure S5A,B). Additionally, the proliferative and metastasizing abilities of MCF-7 cells and T47D cells were also suppressed when treated with entrectinib (Figure S5C–J, *p* < 0.05).

## 4. Discussion

The remarkable clinical success of endocrine therapies in ER-positive BRCA stands as a paradigm for targeted cancer treatment, demonstrating how mechanistic understanding of hormone receptor biology can translate into durable patient benefit [22]. The hormone receptor-positive (ERα+/PR+) and HER2-negative luminal subtypes exhibit superior long-term survival compared to other malignancies [23]. Although endocrine therapy has significantly improved the therapeutic efficiencies of ER-positive BRCA, increasing resistance rates in endocrine therapy have gradually become a more severe problem for the treatment of patients diagnosed with ER-positive BRCA [24]. It is urgent to find a reliable target to promote the sensibility of ER-positive BRCA to endocrine therapy at present.

Tamoxifen, the most widely used endocrine therapy drug for BRCA, is similar to the construction of estradiol [25]. Tamoxifen binds to the estrogen receptor and competitively blocks estrogen binding, thereby suppressing the transcription of downstream oncogenes [26]. A recent study found that intrinsic or acquired resistance to tamoxifen occurs in 30–40% of ER+ BRCA, posing a critical therapeutic challenge that limits durable remission [27]. Current research has identified multiple molecular pathways driving tamoxifen resistance in BRCA; major resistance mechanisms include ERα loss (30%) and ESR1 mutations (Y537S/D538G; 25–40%), with both pathways enabling estrogen-independent proliferation [28,29]. However, Erα is overexpressed or overactivated in many cases with tamoxifen resistance, which reveals that there might be other mechanisms causing resistance by promoting ERα expression or inhibiting its degradation. Recent studies have identified several mechanisms linking ERα expression patterns to tamoxifen resistance. A previous study demonstrated that USP36, a USP family deubiquitinase, directly binds to ERα and stabilizes it by reducing ERα ubiquitination [30]. Similarly, the researchers also found that another DUB PSMD14 could deubiquitinate and stabilize ERα protein to promote the progression and tamoxifen resistance of ER-positive BRCA [31]. These studies all demonstrated that DUBs could be the therapeutic targets to improve the tamoxifen efficiency of BRCA. The ubiquitination status of proteins is dynamically regulated by multiple ubiquitinases and deubiquitinases. Therefore, we aimed to identify additional DUBs that modulate ERα ubiquitination and drive BRCA progression, which may represent promising therapeutic targets for future investigation.

STAMBP is a member of the JAB1/MPN-domain-associated metalloisopeptidase family of deubiquitinases, which comprises an N-terminal MIT domain (microtubule binding), central SH3-binding motif (protein recruitment), and C-terminal JAMM domain (deubiquitinase activity), integrating membrane trafficking with ubiquitin signaling [32,33]. In this study, we employed a DUB-targeted siRNA library and identified STAMBP as a key regulator of ER signaling in ER-positive BRCA cells. The expression levels and prognostic values of STAMBP in ER-positive BRCA were explored by bioinformatic analyses and validated by assays. To investigate STAMBP’s biological functions and mechanisms in ER-positive BRCA, we performed functional experiments and enrichment analyses. These results indicate that STAMBP modulates ER signaling to influence the progression of ER-positive BRCA cells, showing an important significance between STAMBP and ER signaling in BRCA. As a typical deubiquitinase, STAMBP exerts oncogenic effects in multiple malignancies by directly binding to substrates and inhibiting their polyubiquitination. Therefore, we sought to investigate the molecular mechanisms underlying the interaction between STAMBP and ERα in BRCA cells. Further investigation revealed that STAMBP binds to ERα and enhances its stability through deubiquitination, thereby promoting the progression of BRCA cells.

Tamoxifen resistance was also reported to be induced by the mutation of ERα at the Y537S site [34,35]. Interestingly, we found that the high expression of STAMBP might be correlated with worse prognosis in the patients who were only treated by tamoxifen, according to the data in an online database. This finding suggests STAMBP may play a critical role in mediating tamoxifen resistance in patients. To test this hypothesis, we established a tamoxifen-resistant cell model using a Y537S mutation plasmid. We observed that STAMBP suppression substantially reduced both wild-type and mutant ERα protein expression. Both in vitro cellular assays and in vivo subcutaneous tumor models demonstrated that combining STAMBP inhibition with tamoxifen significantly enhanced therapeutic efficacy, confirming our hypothesis. These results further support the potential efficiency of improving endocrine therapy by inhibiting STAMBP, which could be explored by more pharmacological research. Our findings further demonstrate the anti-tumor efficacy of the STAMBP inhibitor entrectinib in BRCA cells, underscoring its promise as a therapeutic candidate for future development. While limitations remain in our current study, we plan to utilize STAMBP inhibitors in endocrine resistance models to further validate their clinical relevance in the future.

## 5. Conclusions

In conclusion, we have demonstrated the oncogenic roles of STAMBP in ER-positive BRCA through bioinformatic analyses and experimental validation. However, there were still some limitations in this study. We did not have enough data from clinical cohorts to validate the prognostic value of STAMBP. In the future, more clinical samples and prognostic values could be collected to validate our findings. More kinds of resistance models will be used to validate the correlation between STAMBP and tamoxifen resistance of BRCA. Future pharmacological and clinical studies will focus on exploring the clinical potential of STAMBP inhibition as a therapeutic strategy for BRCA. Above all, we found that high expression of STAMBP might be correlated with the poor prognosis of ER-positive BRCA. STAMBP could directly bind to ERα protein and decrease the K48-linked ubiquitination levels of ERα protein, which might contribute to the progression and tamoxifen resistance of BRCA cells. This finding revealed that STAMBP might become a potential target for improving tamoxifen therapeutic efficiencies in BRCA patients.

## Data Availability

The datasets used to support the findings of this study are available from the corresponding author upon request.

## Supplementary Materials

The following supporting information can be downloaded at: https://www.mdpi.com/article/10.3390/biom15111502/s1, Figure S1: High STAMBP expression is correlated with poor DMFS and DFS rates in ERα-positive BRCA; Figure S2: STAMBP knockdown inhibits EMT signaling in ERα-positive BRCA cells; Figure S3: The catalytically inactive STAMBP mutant (D348A) fails to reduce ERα polyubiquitination levels; Figure S4: Suppression of STAMBP recovered the effects of tamoxifen to suppress ERα signaling in endocrine-resistant BRCA cells; Figure S5: Entrectinib inhibits STAMBP expression and tumor progression in ER positive BRCA cells; Table S1: List of primer sequences; Table S2: List of transfection sequences; Table S3: List of primary and secondary antibodies; Table S4: List of DUB genes that regulate GREB1 expression in T47D cells; Table S5: Cox regression analyses of STAMBP in BRCA patients.

## Abbreviations

The following abbreviations are used in this manuscript:

BRCA	Breast cancer
CCK-8	Cell Counting Kit-8
CHX	Cycloheximide
Co-IP	Co-immunoprecipitation
DEG	Differential-expressed gene
DFS	Disease-free survival
DMFS	Distant metastasis-free survival
DUB	Deubiquitinase
EDU	5-ethynyl-2′-deoxyuridine
EGA	European Genome-phenome Archive
EMT	Epithelial–mesenchymal transition
ER	Estrogen receptor
ERE	Estrogen response element
FDR	False discovery rate
GEO	Gene Expression Omnibus
IF	Immunofluorescence
IHC	Immunohistochemistry
JAMM	JAB1/MPN-domain-associated metalloisopeptidase
K-M	Kaplan–Meier
MCPIP	Monocyte chemotactic protein-induced protein
MINDY	Motif-interacting with ubiquitin-containing novel DUB family
MJD	Machado–Joseph disease protein domain protease
OS	Overall survival
OTU	Ovarian tumor-related protease
qRT-PCR	Quantitative real-time PCR
RFS	Recurrence-free survival
shRNA	Short hairpin RNA
siRNA	Small interfering RNA
STAMBP	STAM binding protein
TCGA	The Cancer Genome Atlas Program
UCH	Ubiquitin C-terminal hydrolase
USP	Ubiquitin-specific protease
WB	western blot
