# Supplementary material for: STAMBP Accelerates Progression and Tamoxifen Resistance of Breast Cancer Through Deubiquitinating ERα
Source: Biomolecules. 2025 Oct 24;15(11):1502. doi: 10.3390/biom15111502 (PMC12650272; doi:10.3390/biom15111502)

Figure 2B

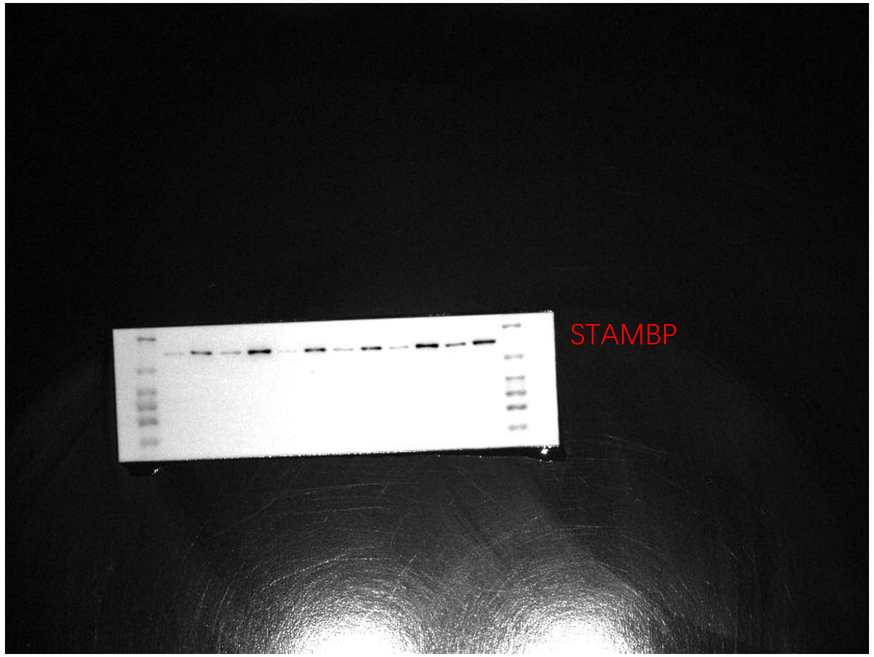

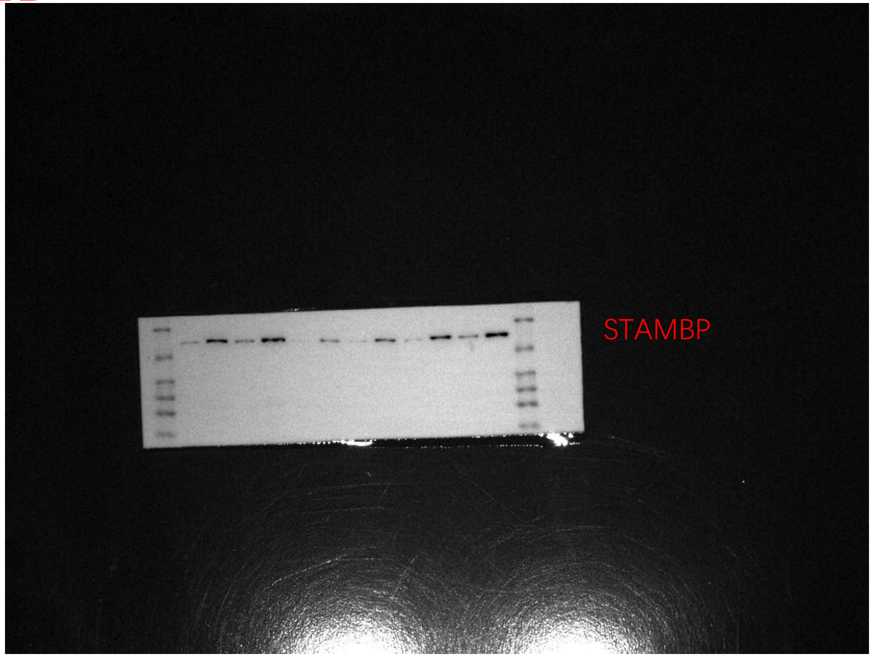

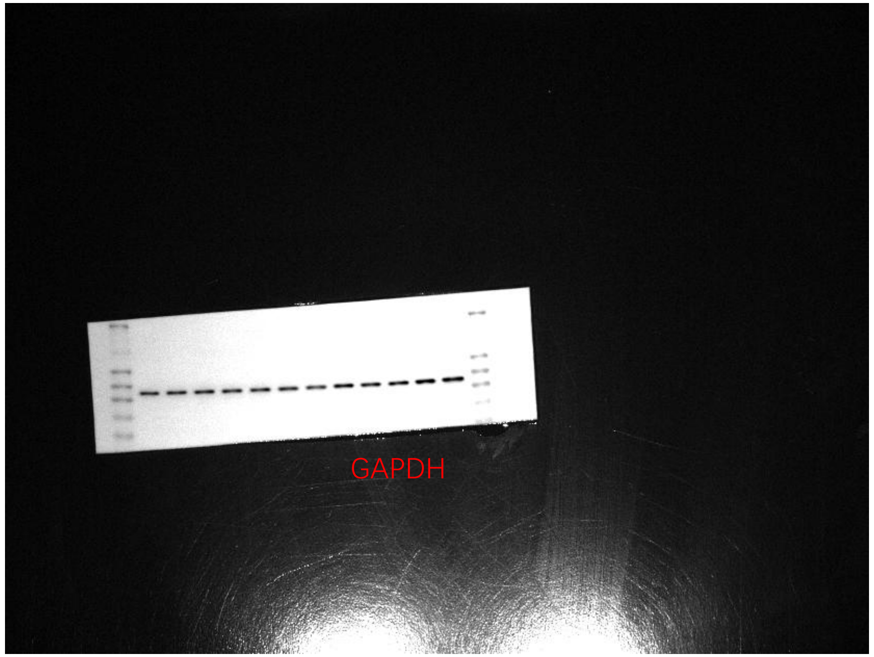

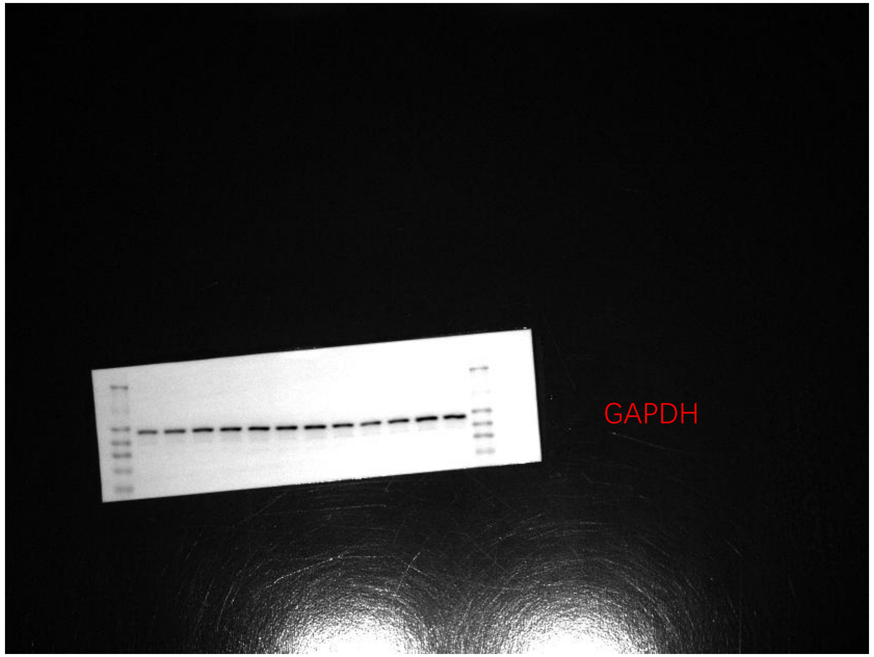

Figure 2G

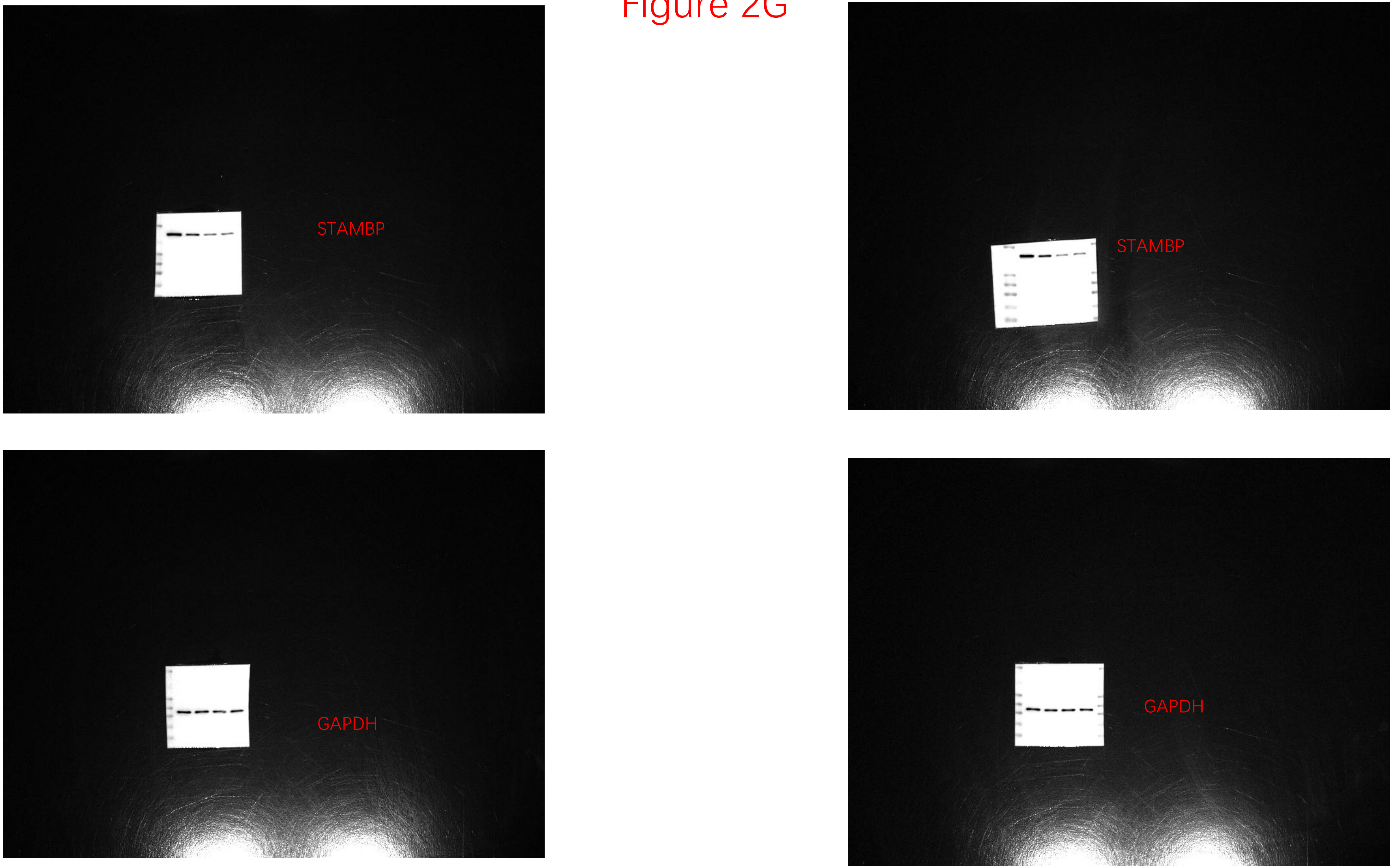

Figure 3E

MCF-7

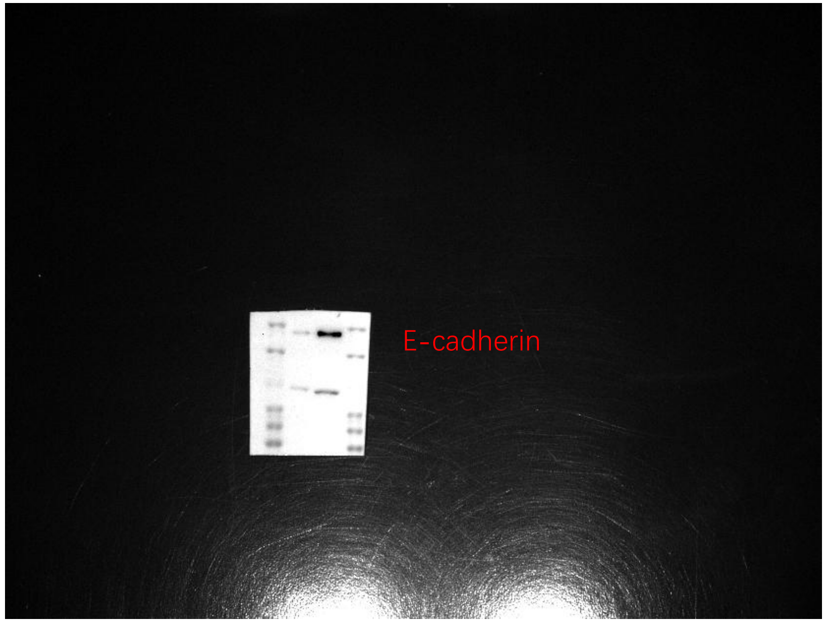

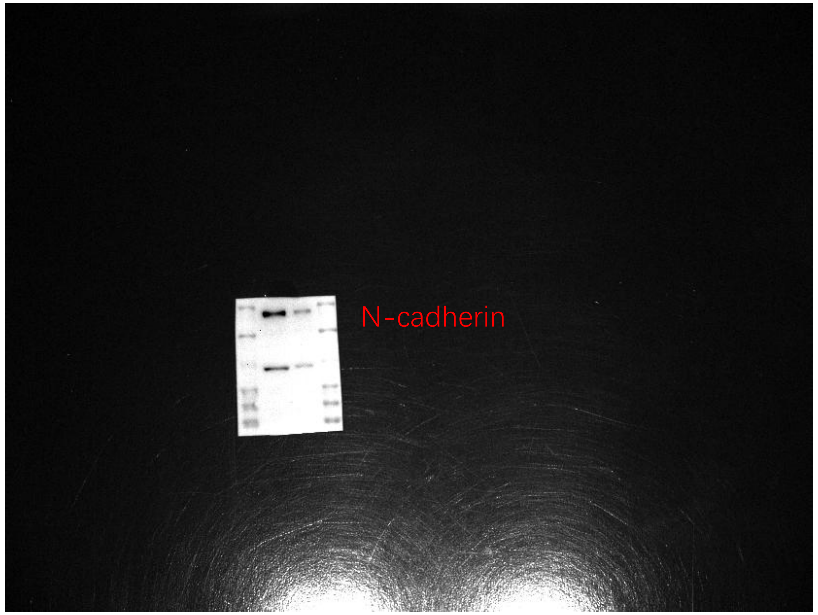

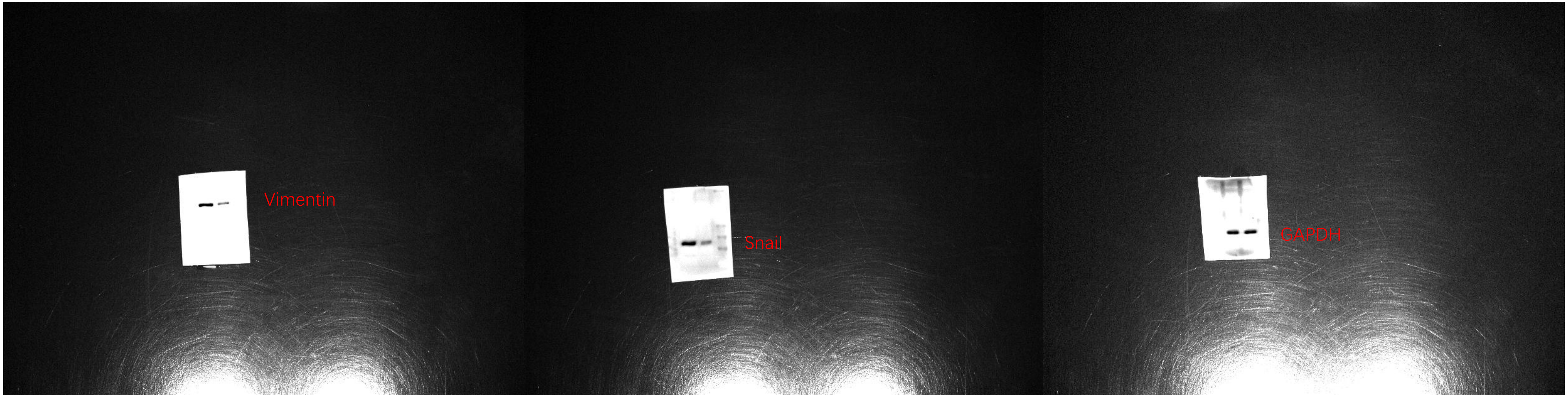

Figure 3E
T47D

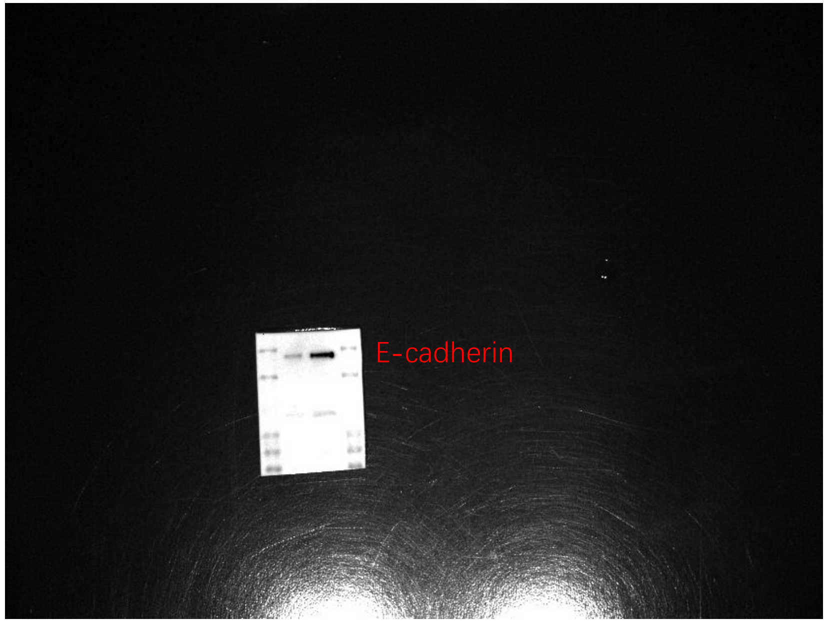

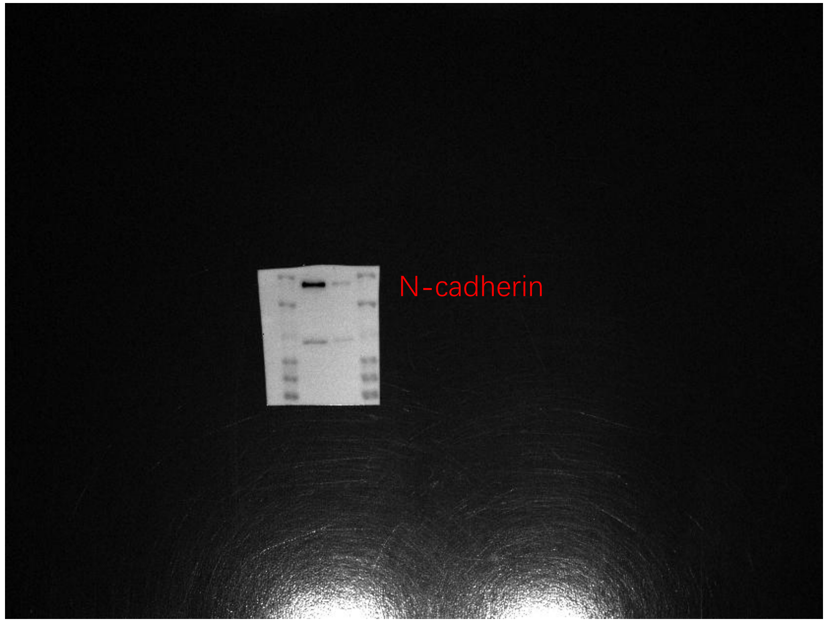

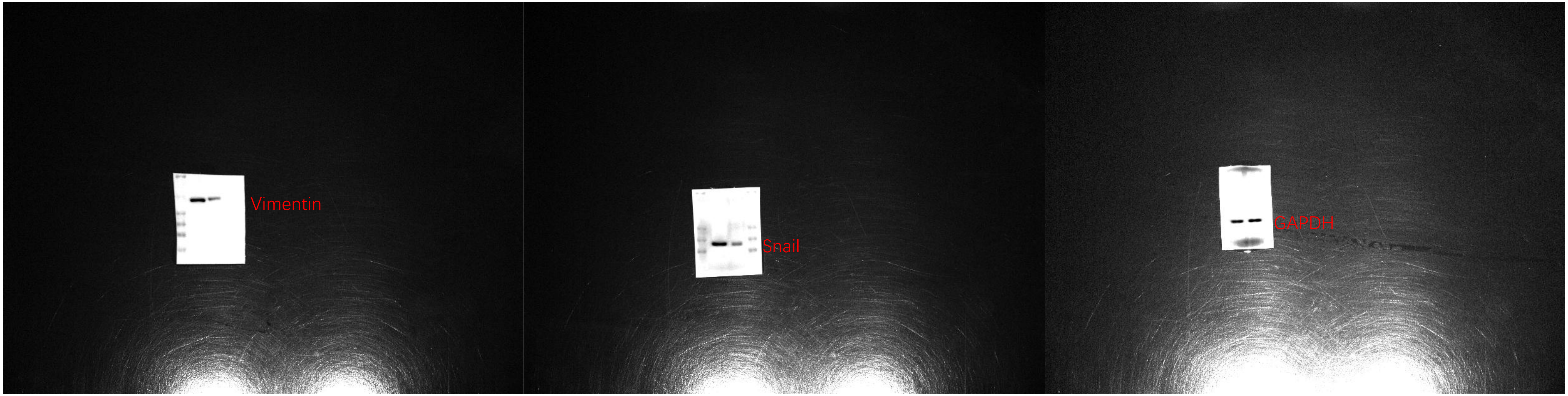

Figure 3F
MCF-7

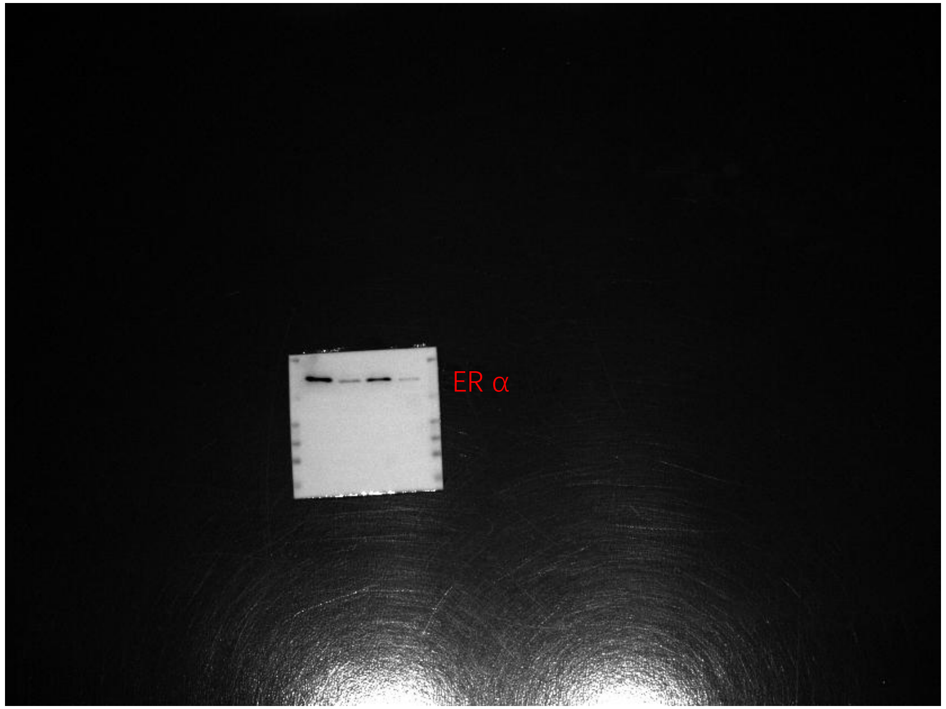

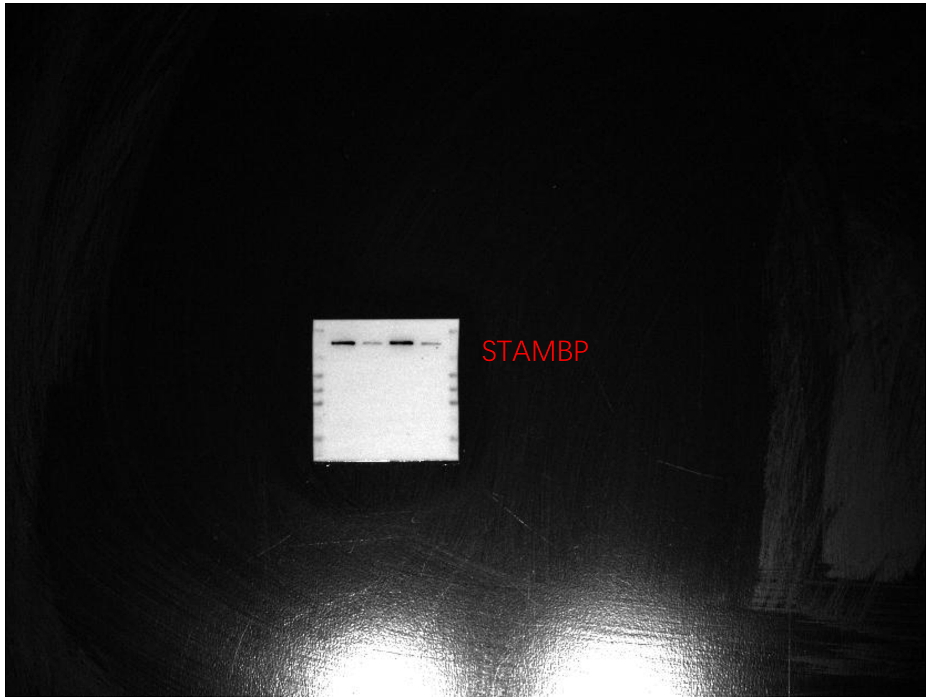

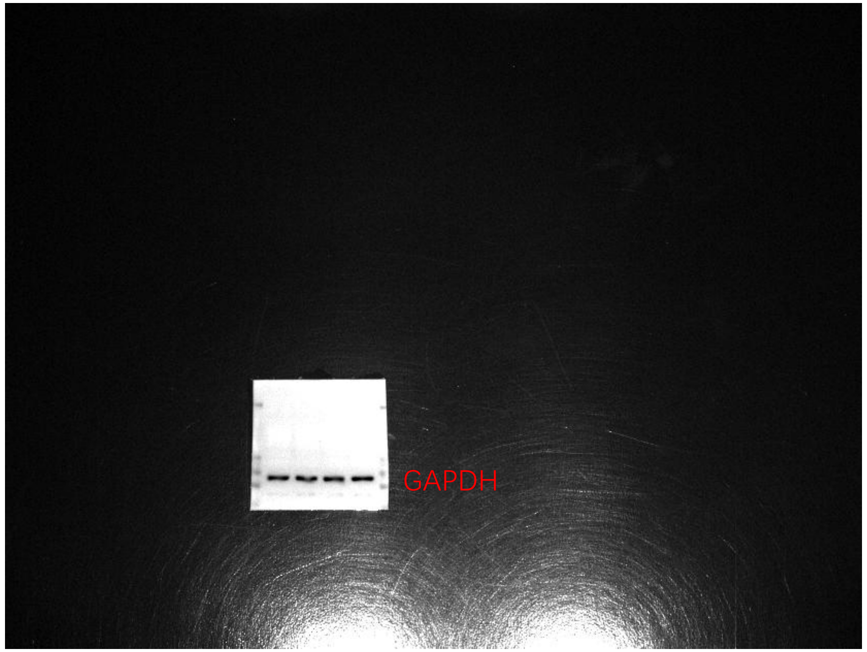

Figure 3G
T47D

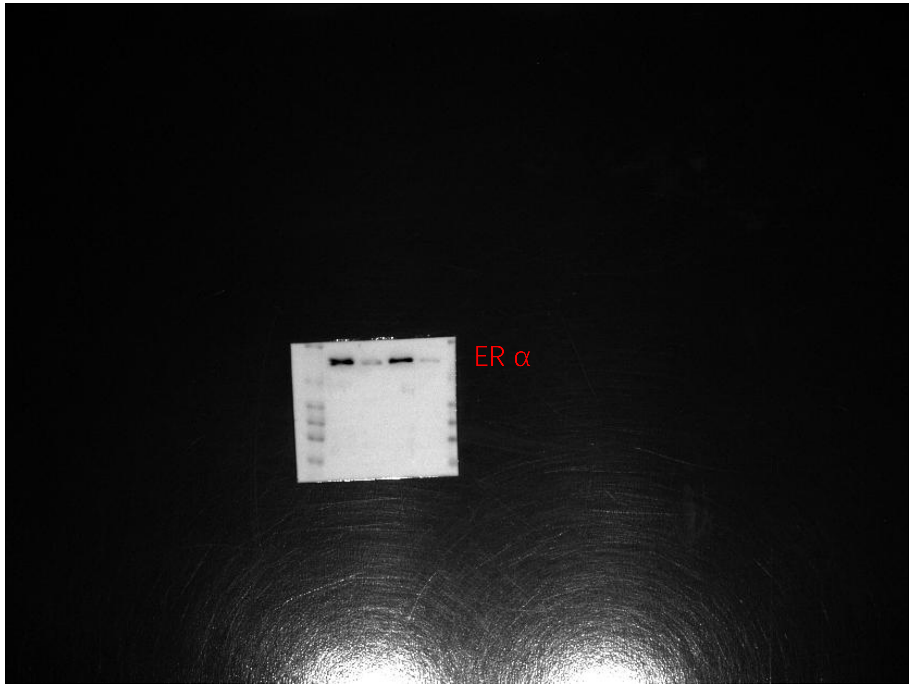

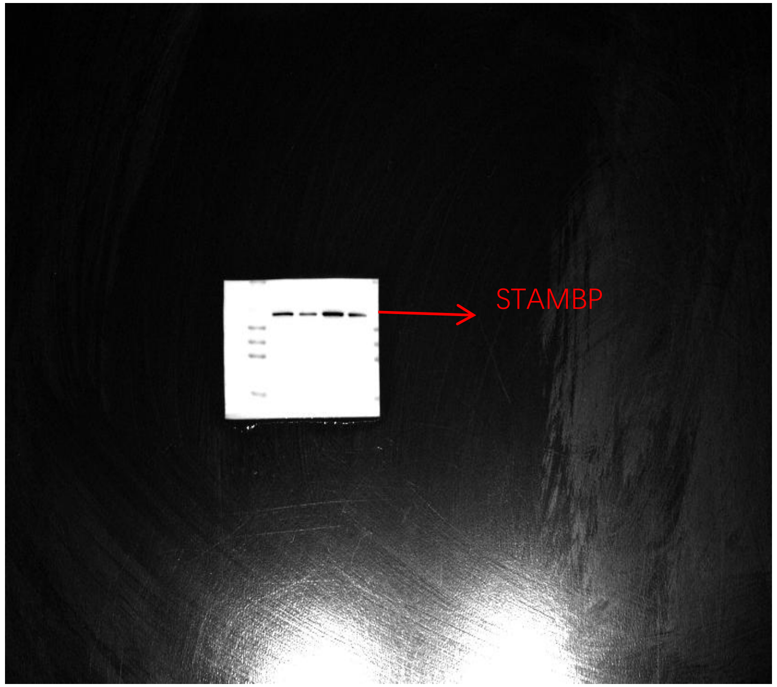

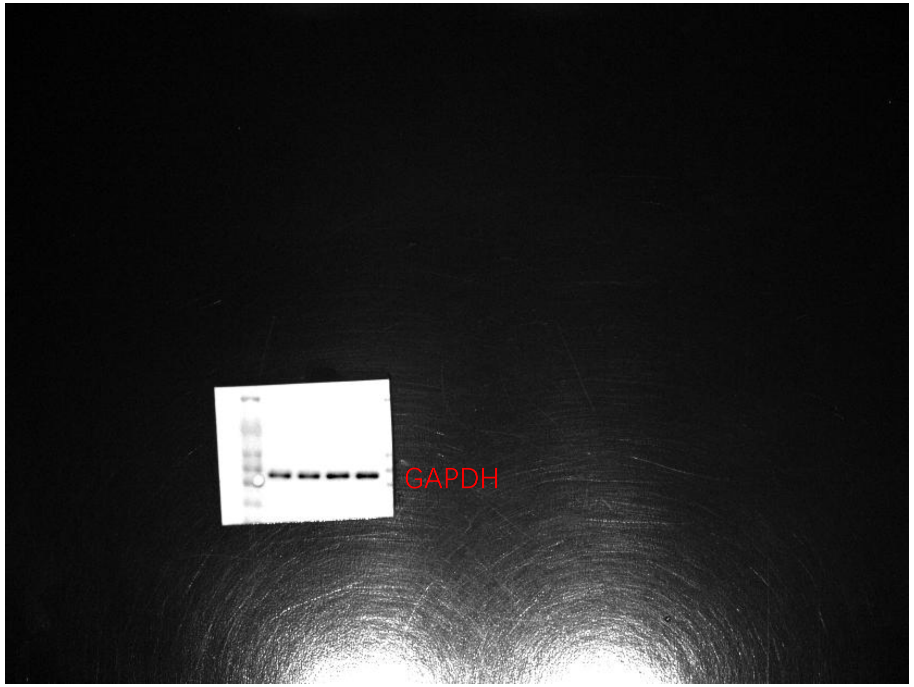

Figure 3H
MCF-7

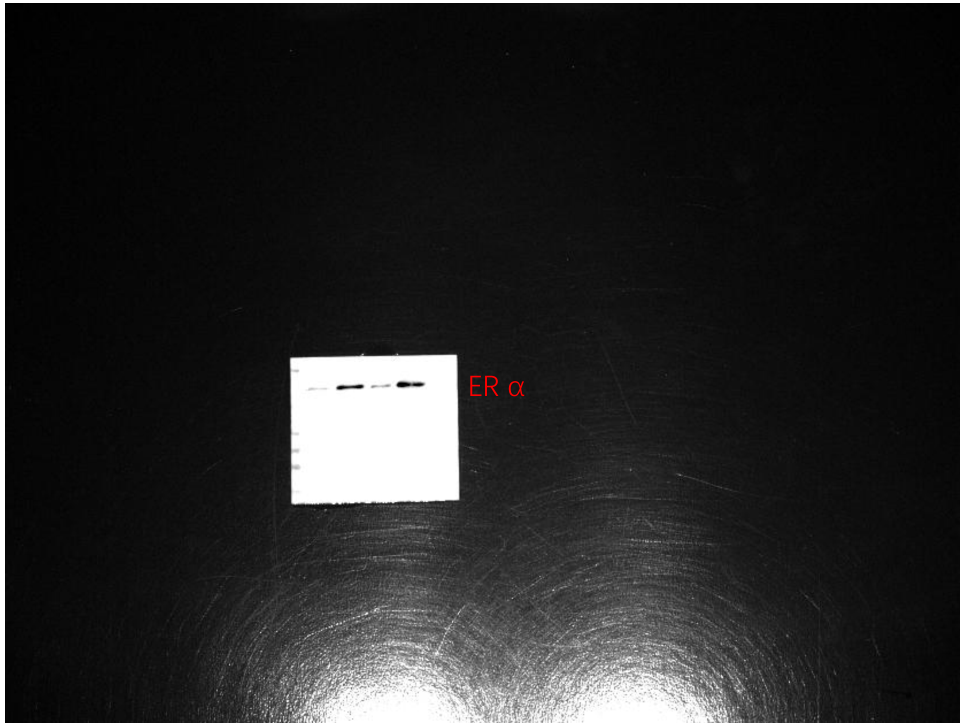

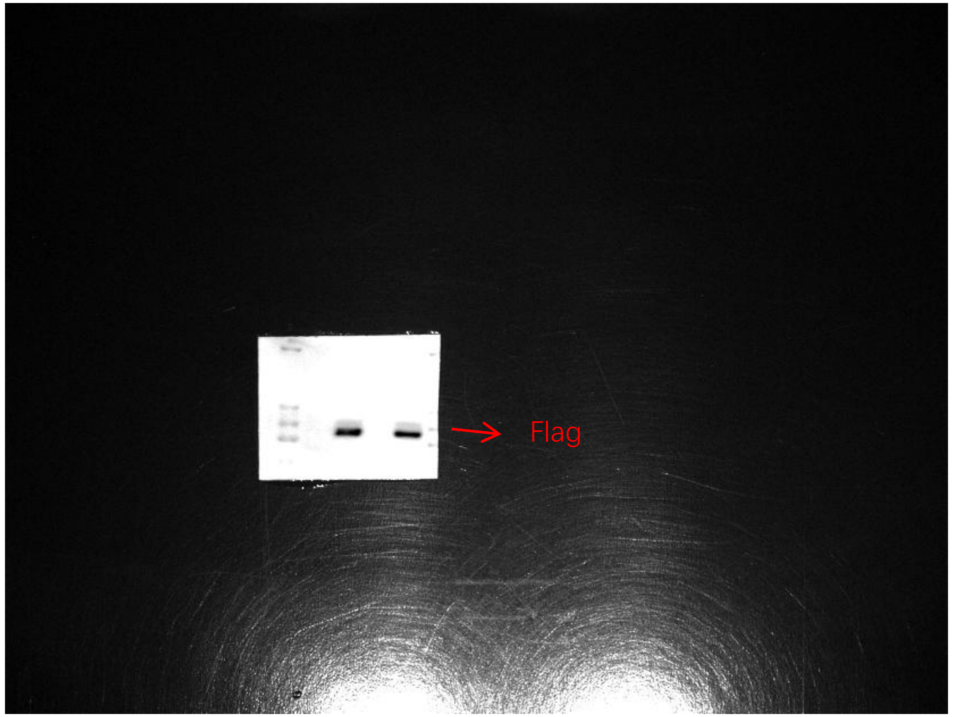

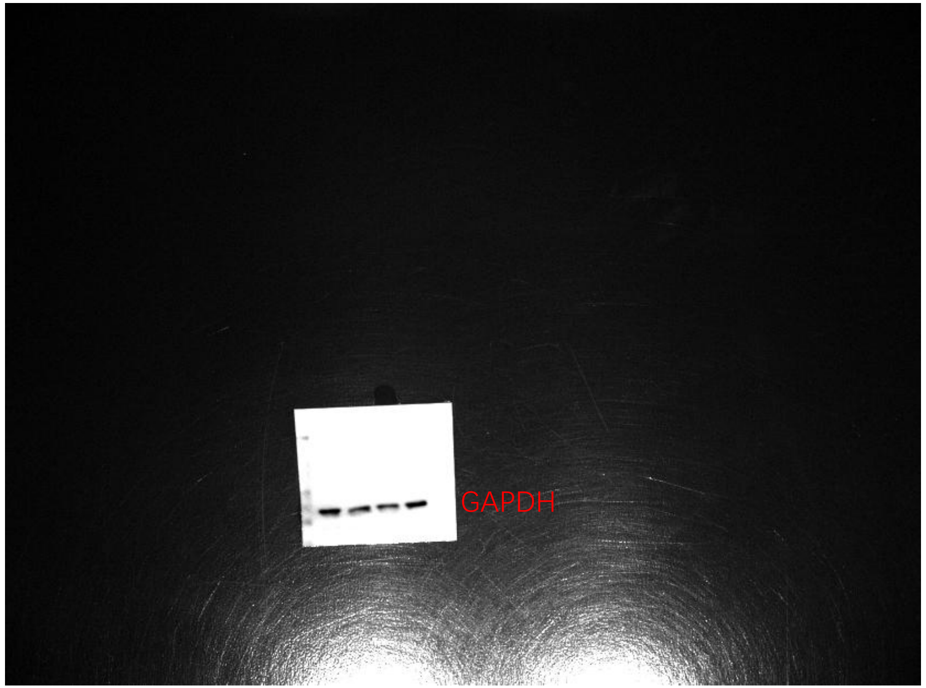

Figure 3I
T47D

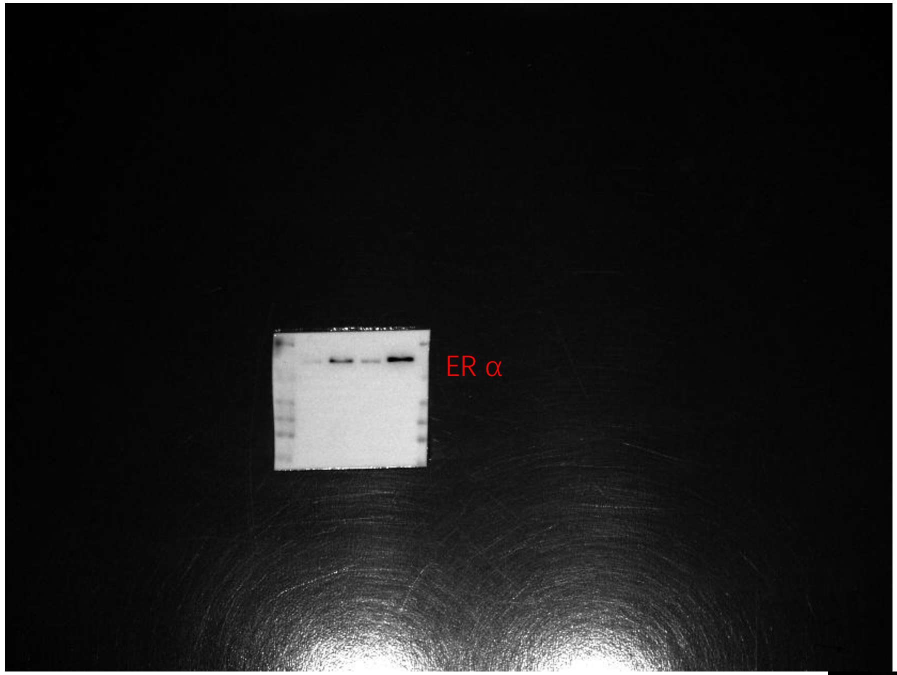

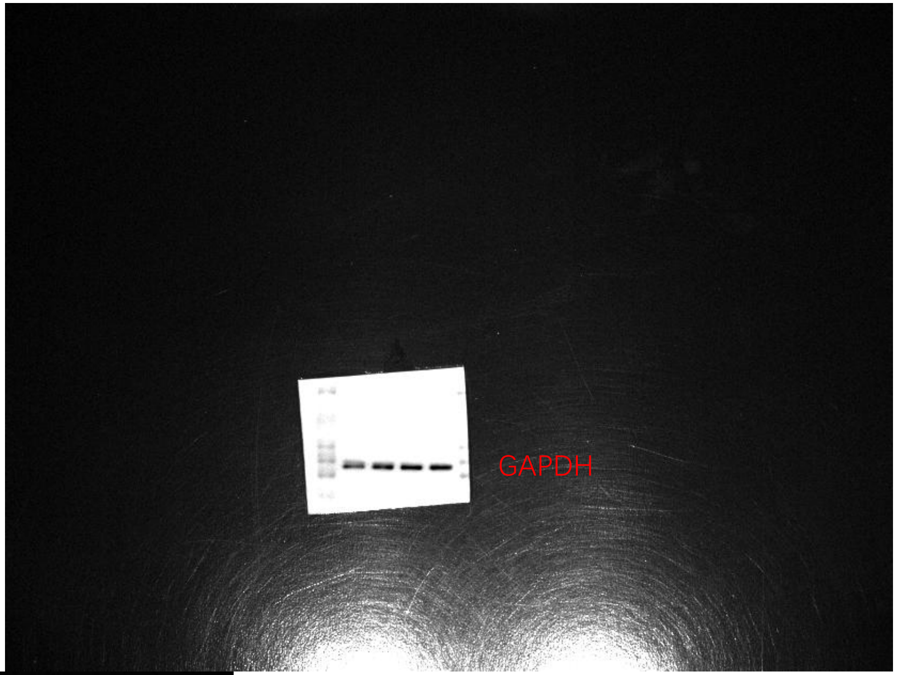

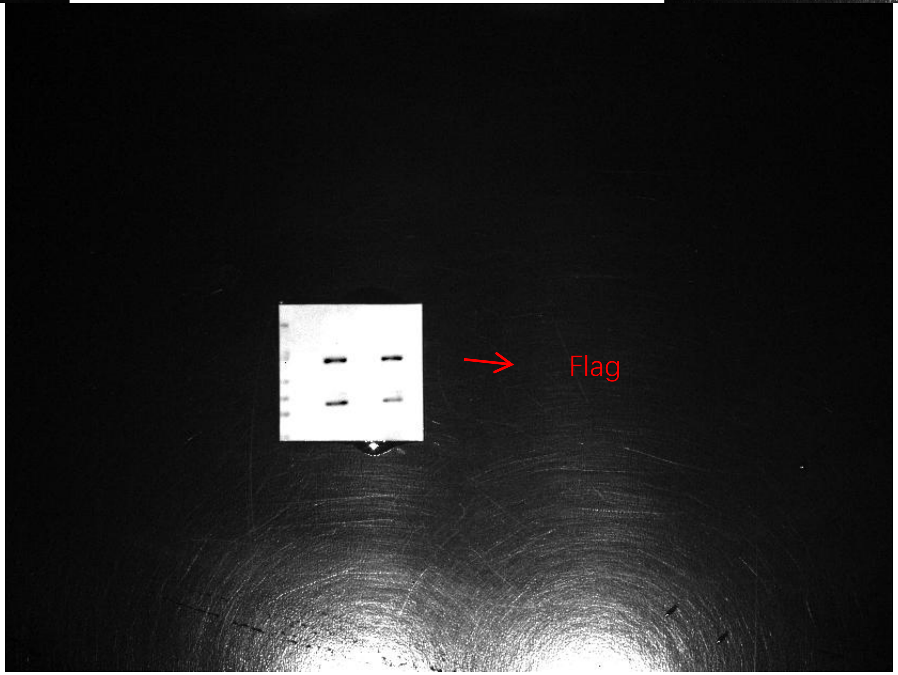

Figure 4I MCF-7

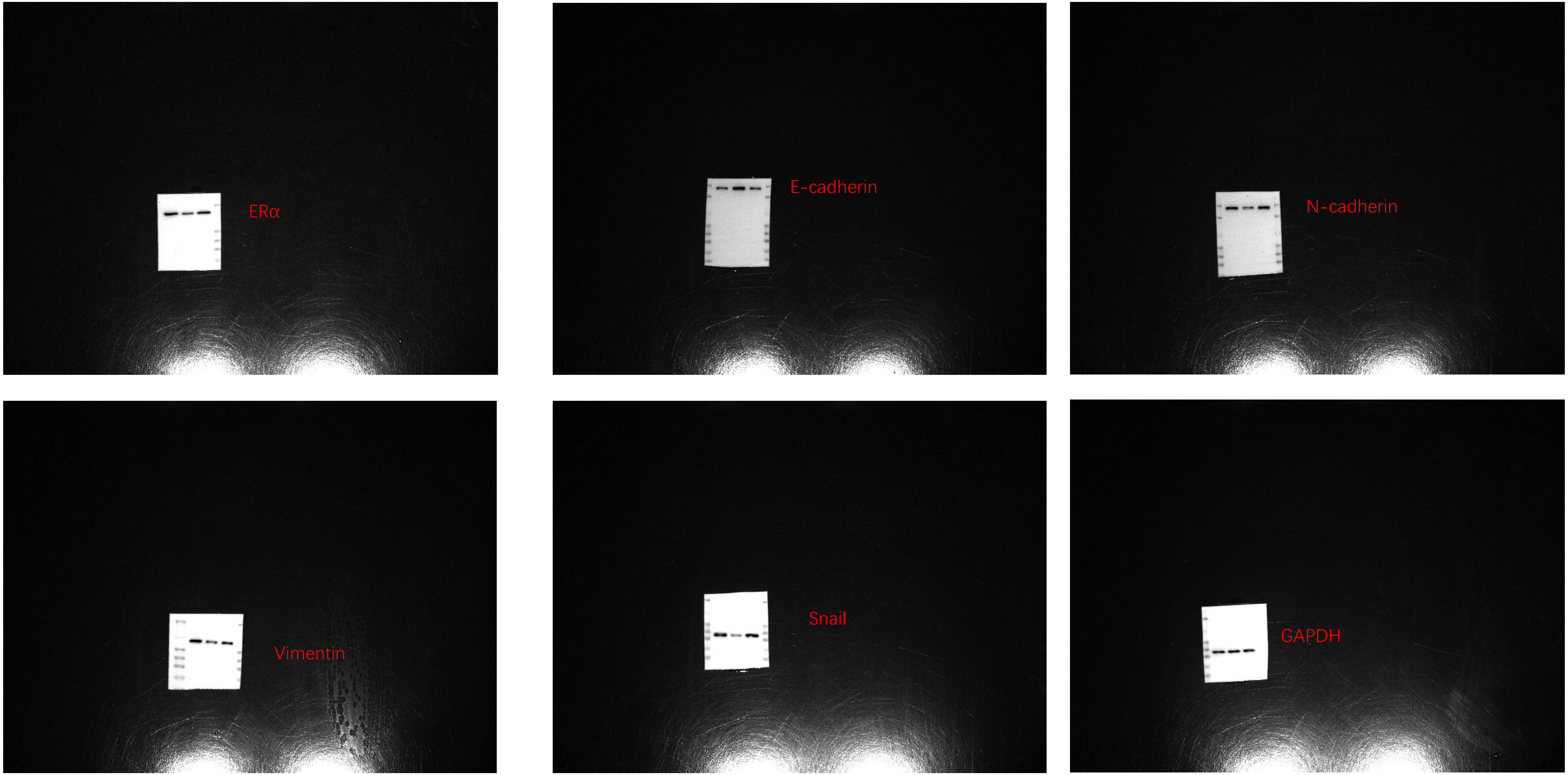

Figure 4I T47D

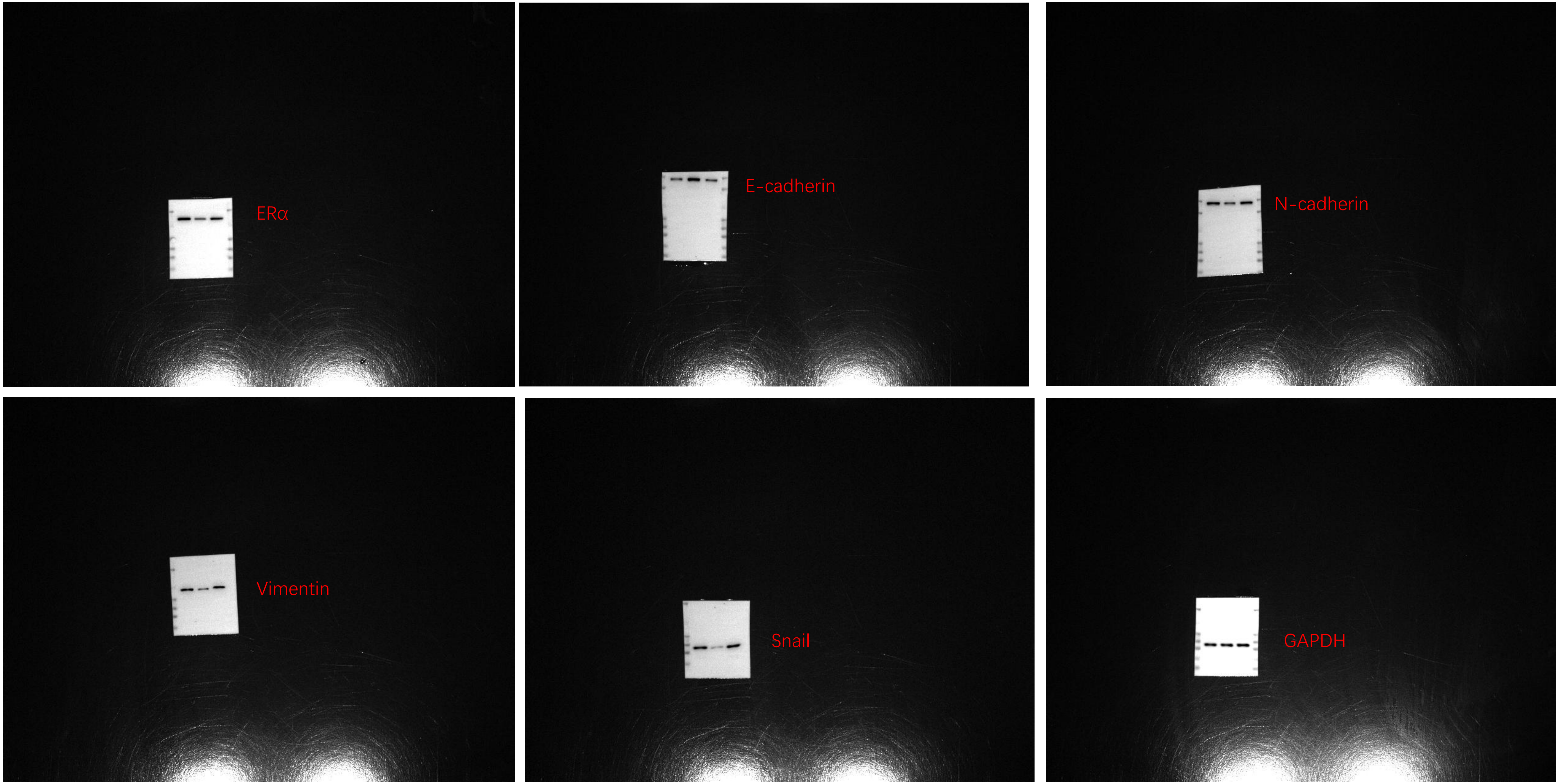

Figure 5B

MCF-7

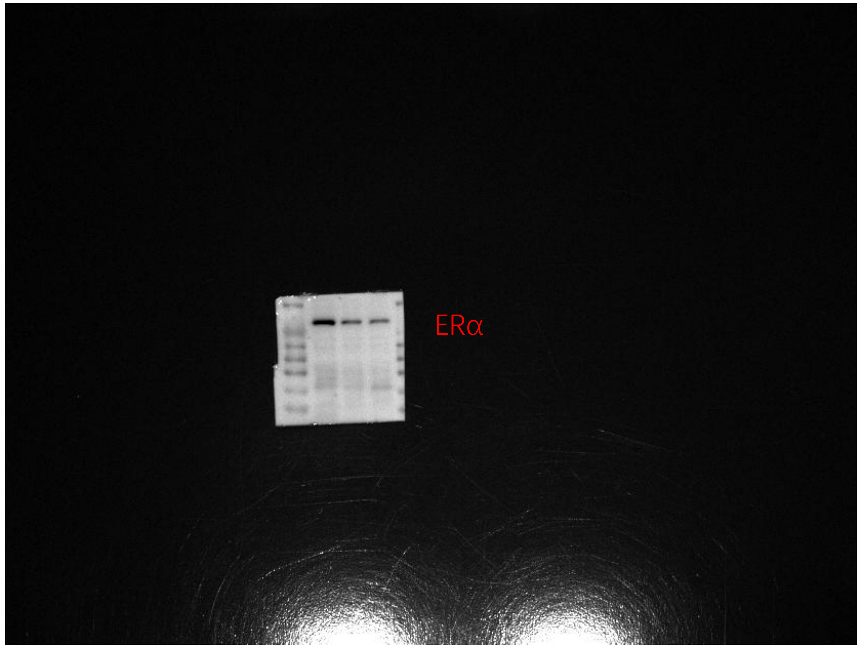

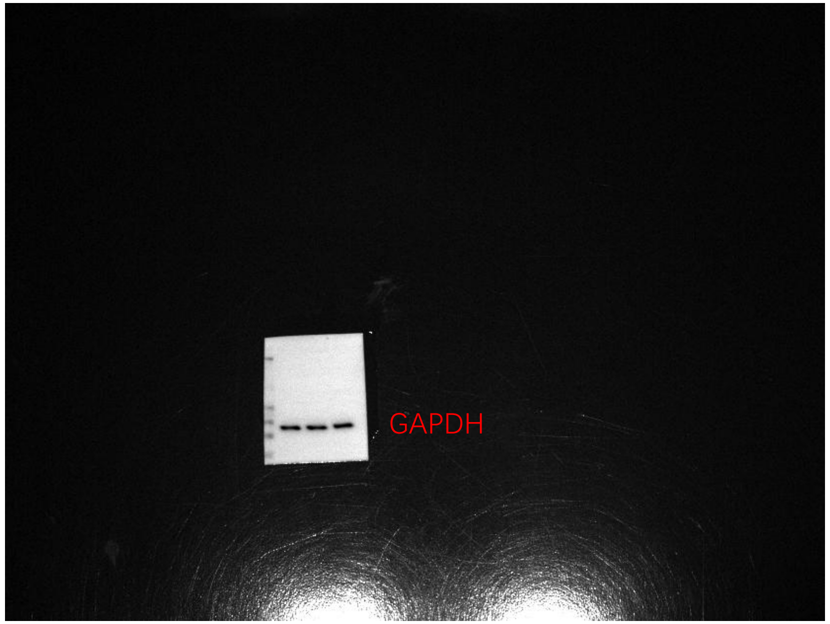

T47D

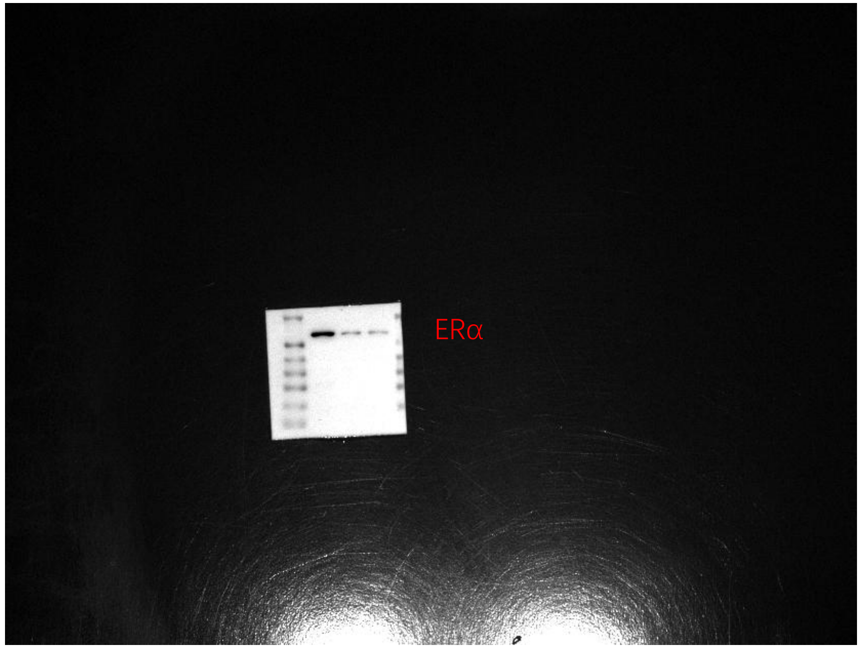

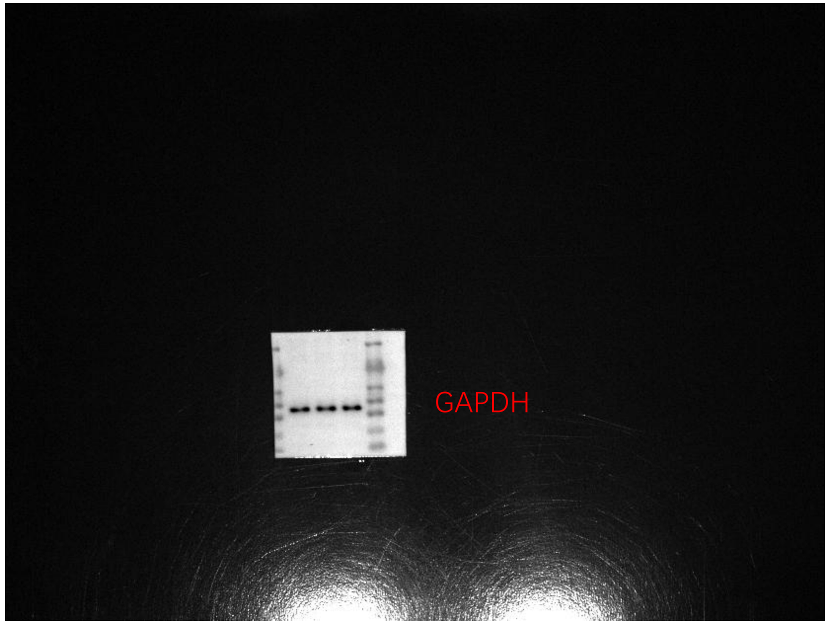

Figure 5D

MCF-7

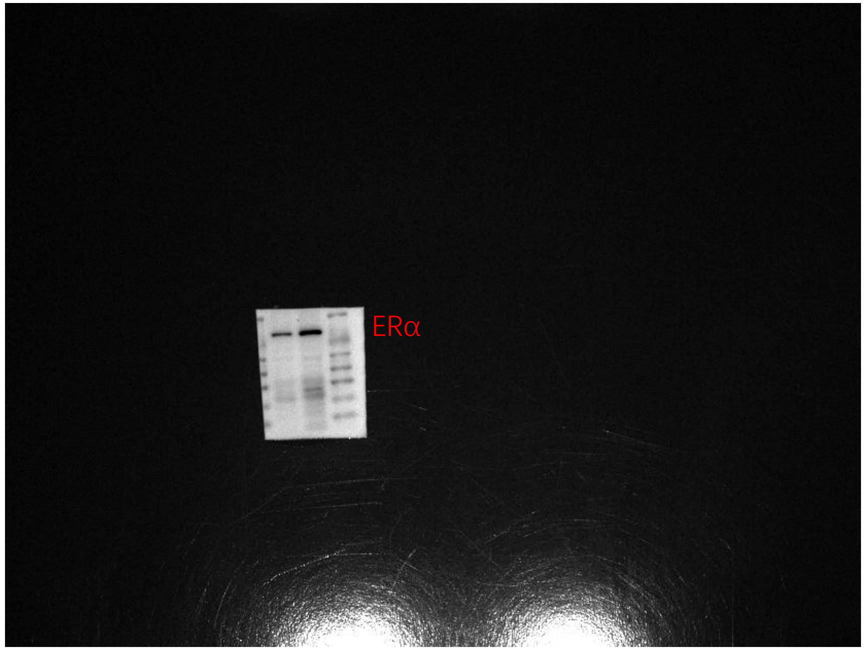

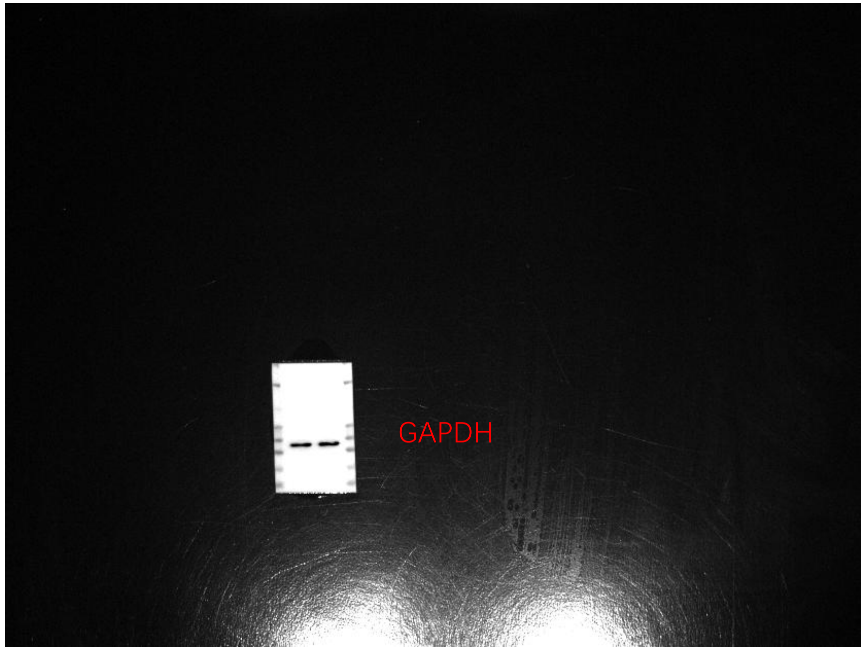

T47D

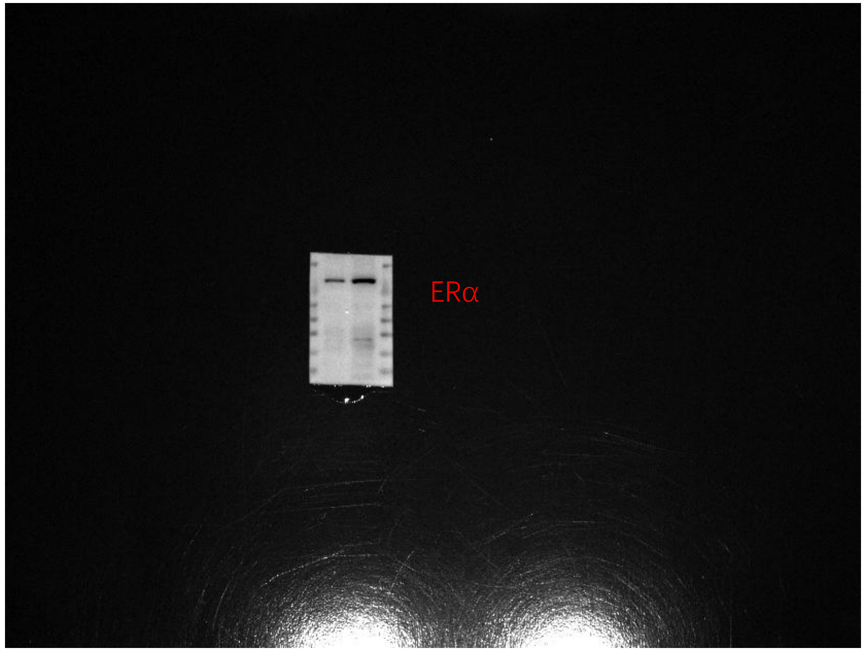

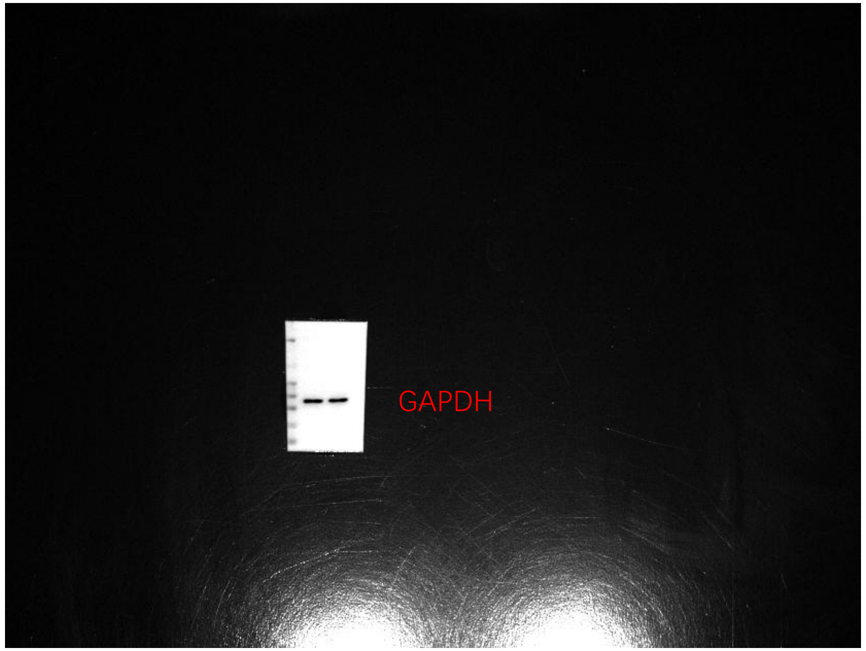

Figure 5E MCF-7

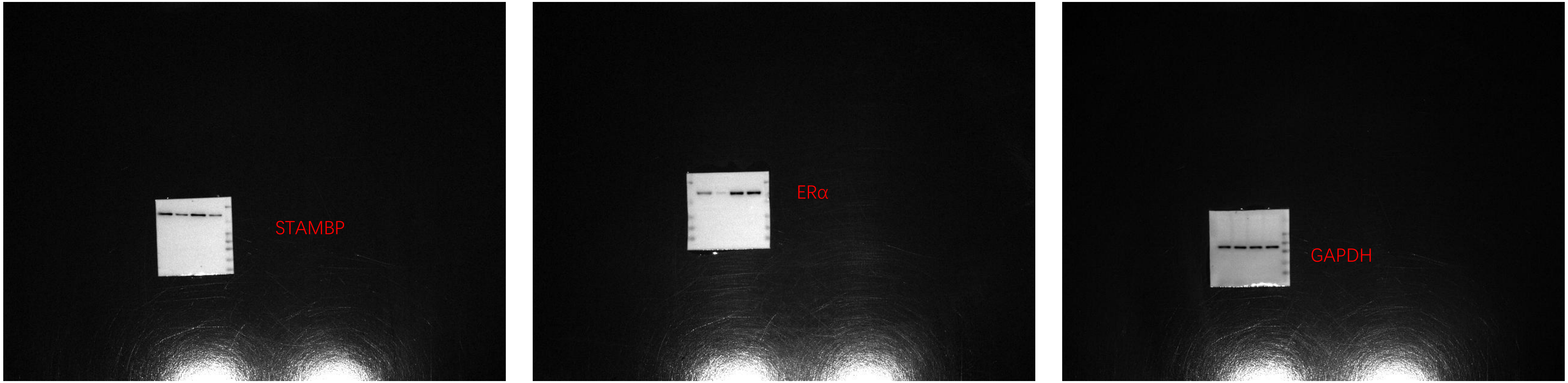

T47D

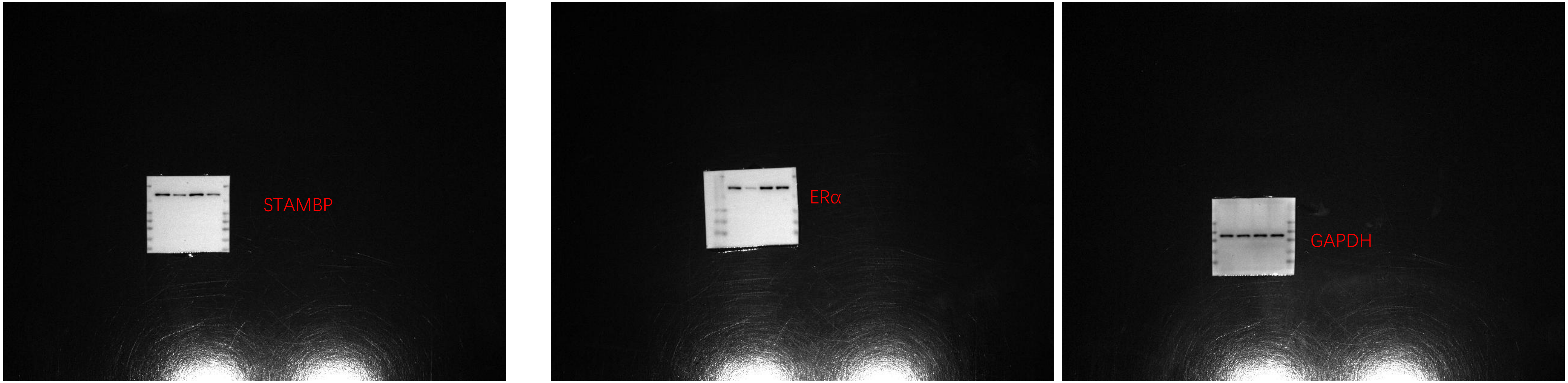

Figure 5F MCF-7

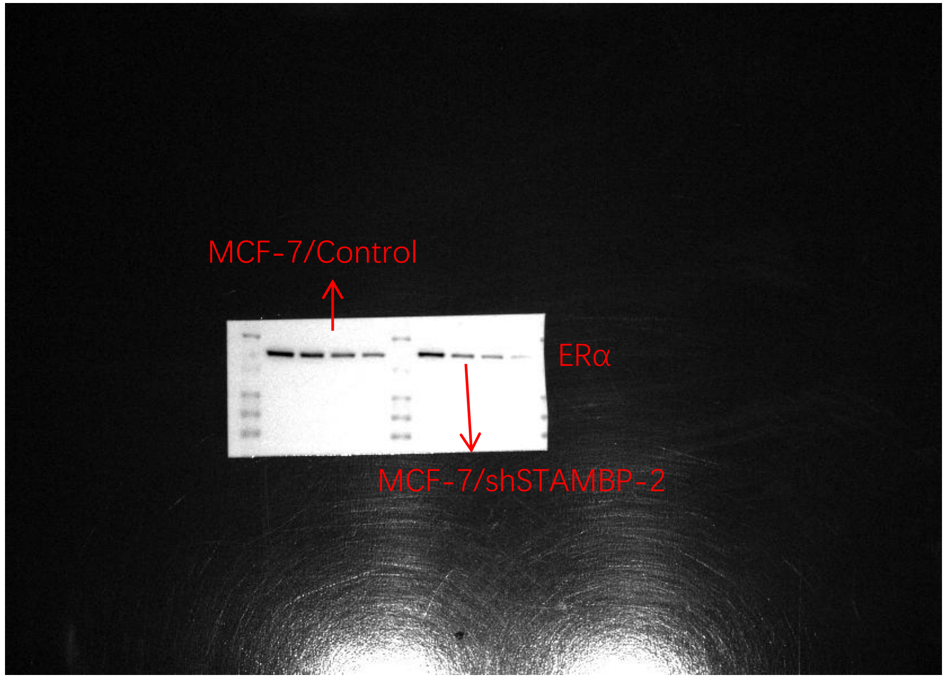

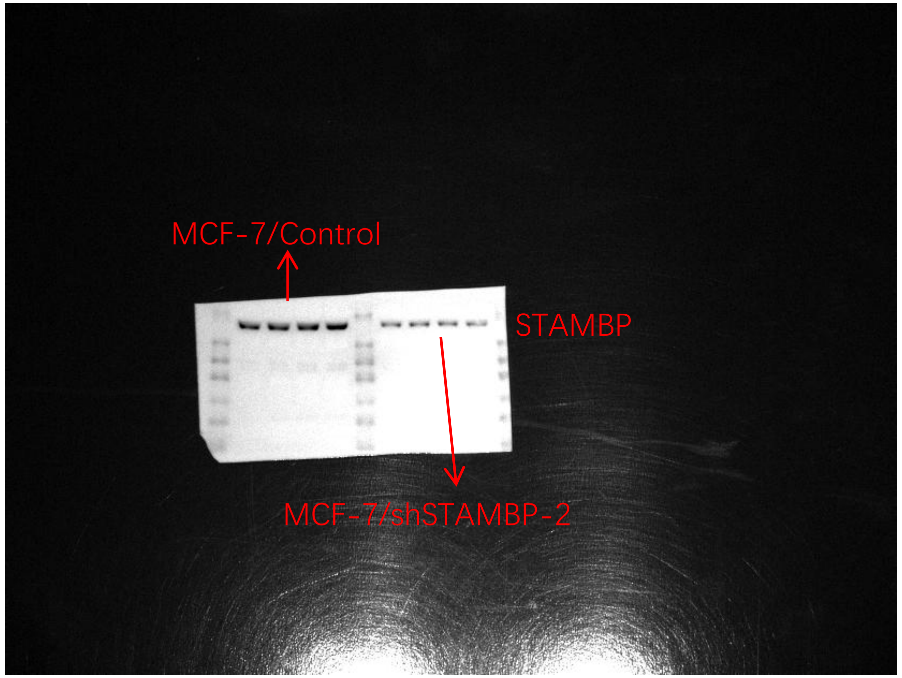

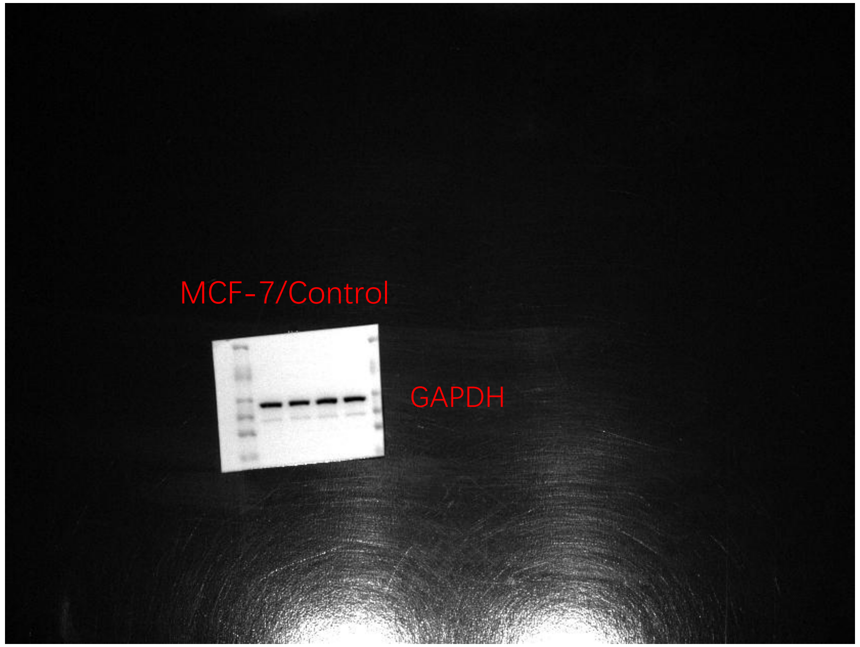

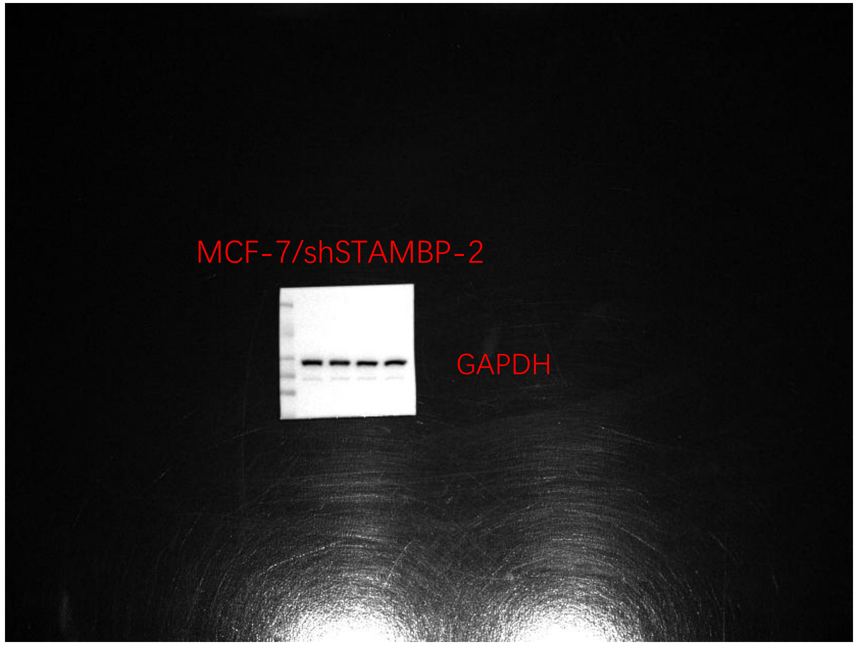

Figure 5G T47D

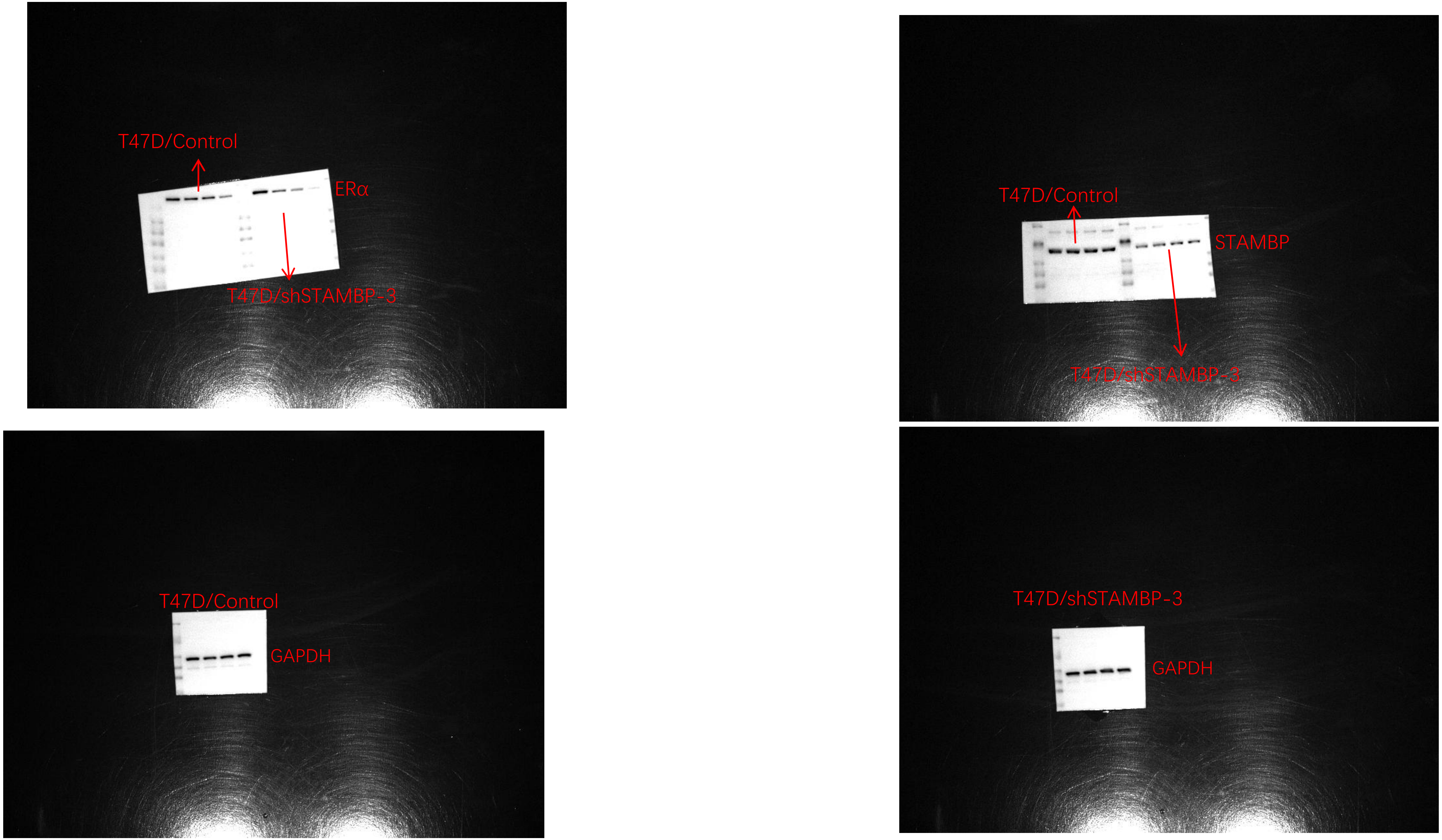

Figure 5K MCF-7

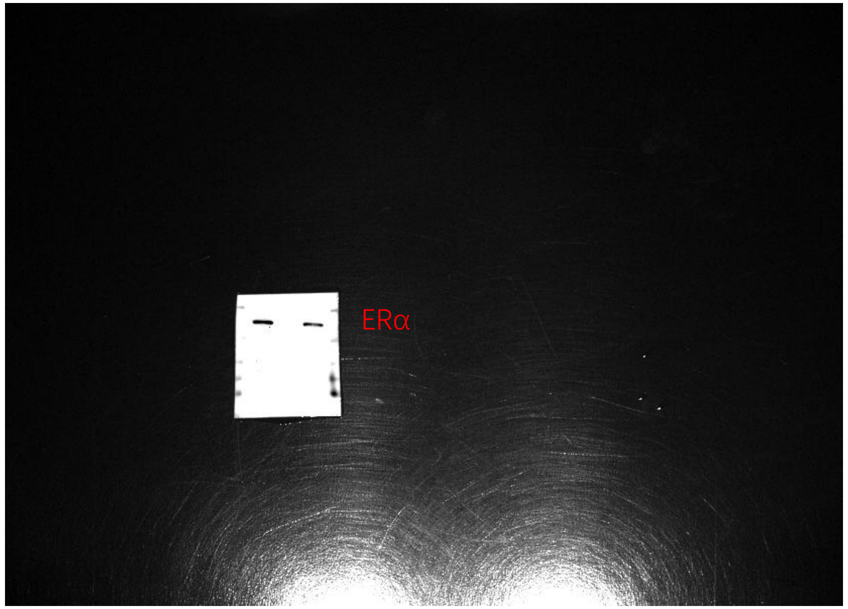

IP:STAMBP

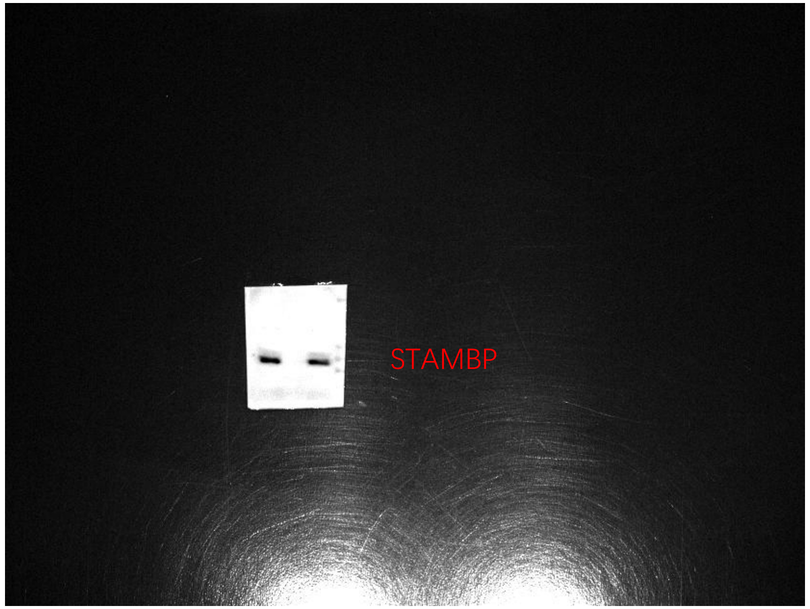

IP:STAMBP

MCF-7

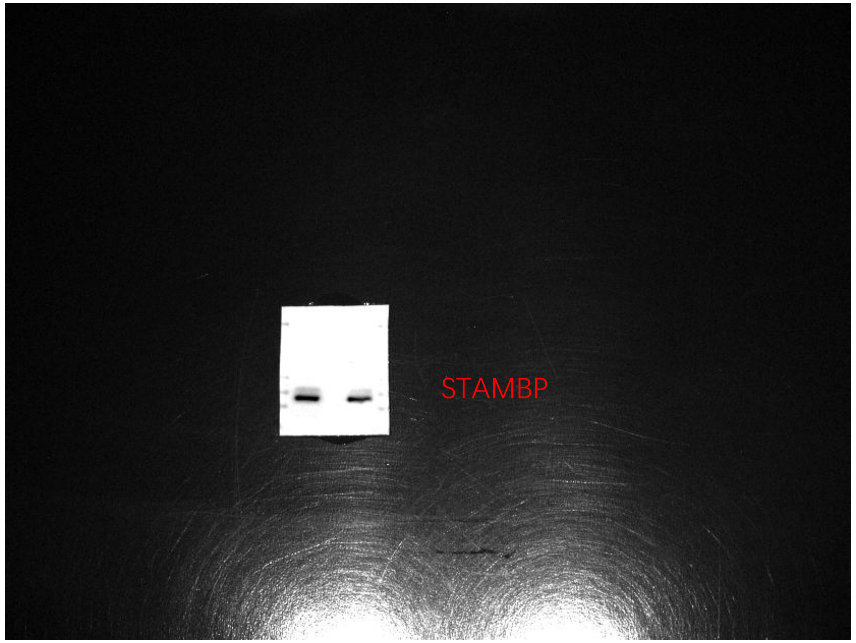

IP:ERα

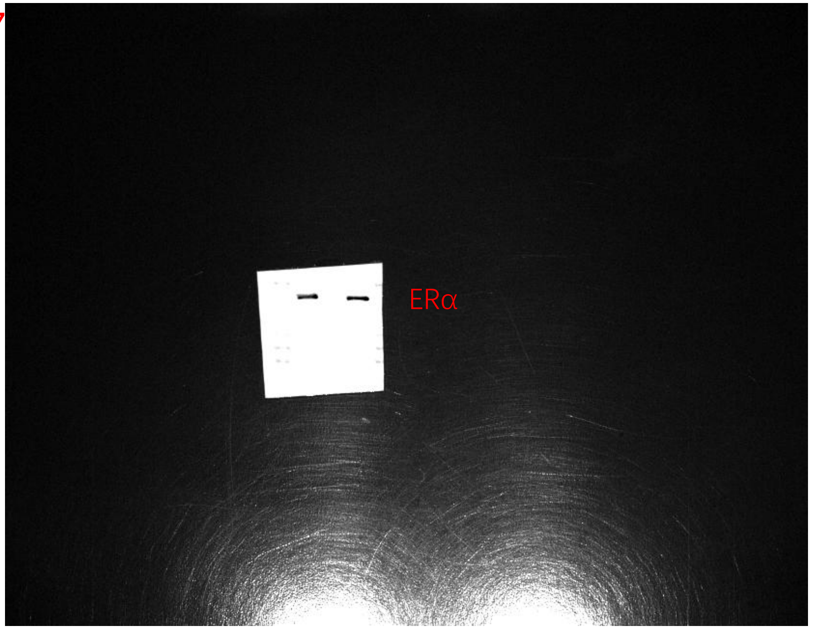

IP:ERα

Figure 5K

T47D

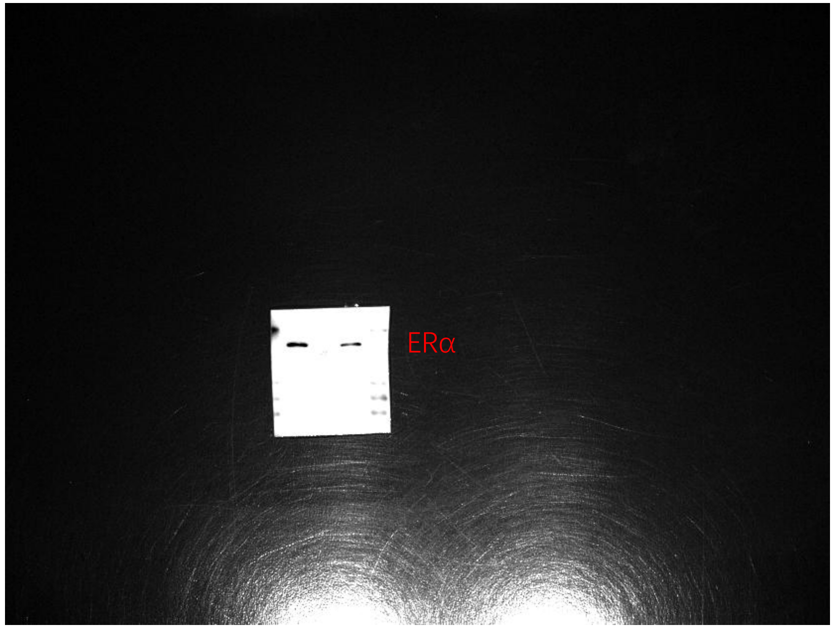

IP:STAMBP

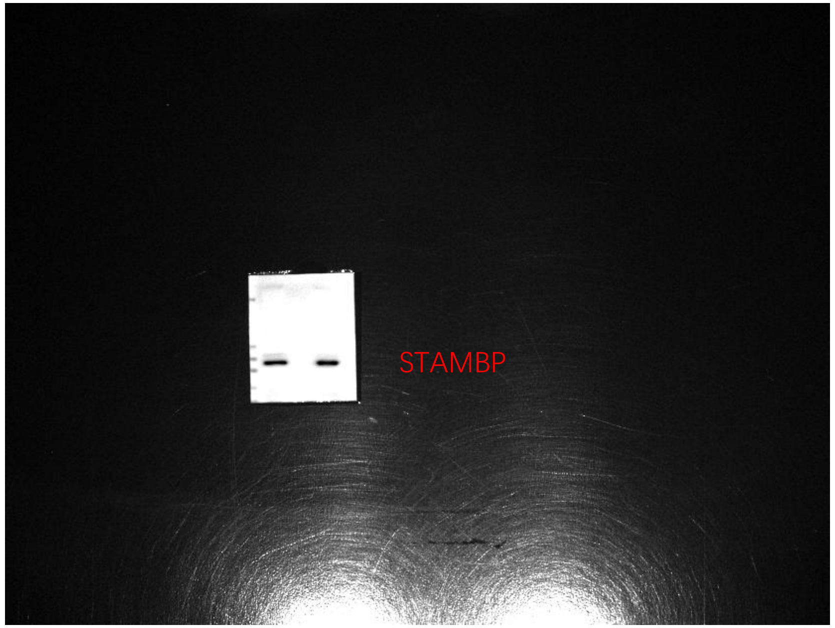

IP:STAMBP

T47D

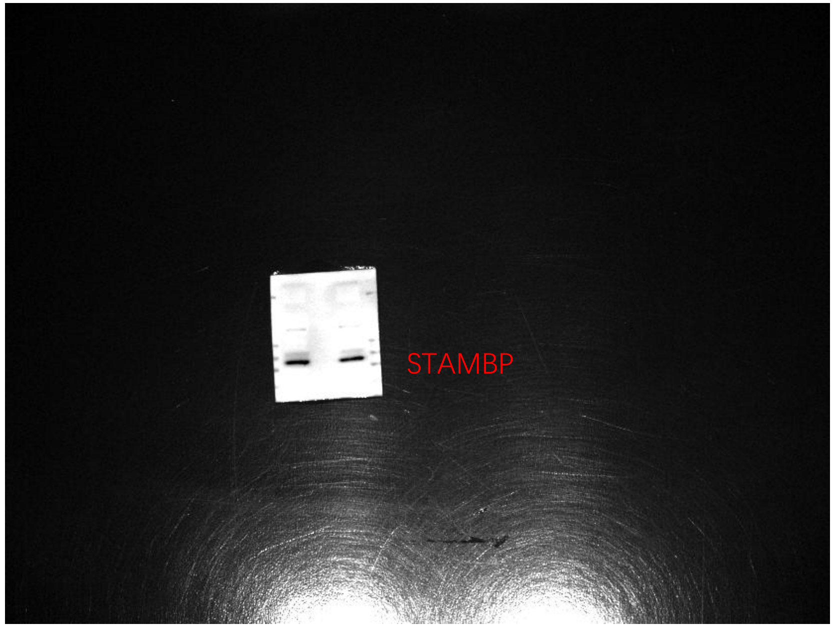

IP:ERα

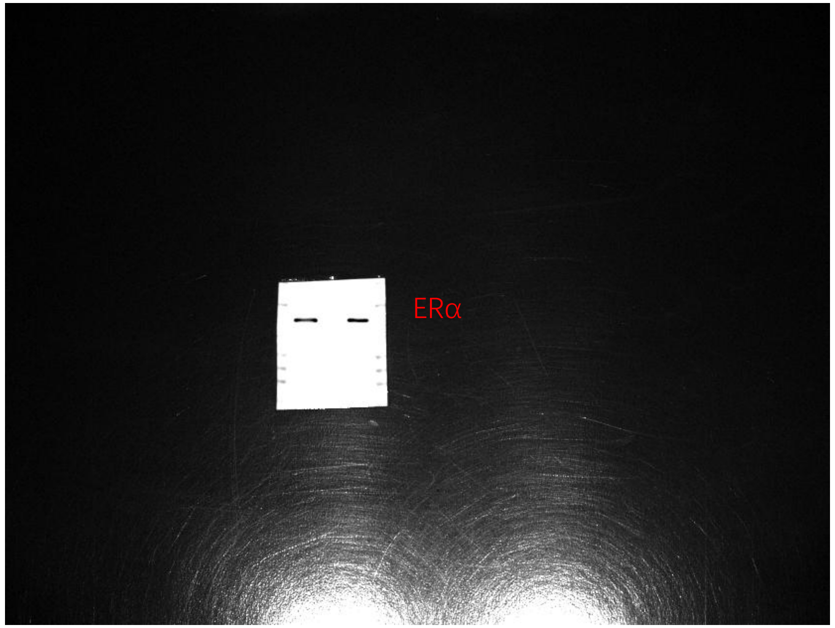

IP:ERα

Figure 5L
MCF-7

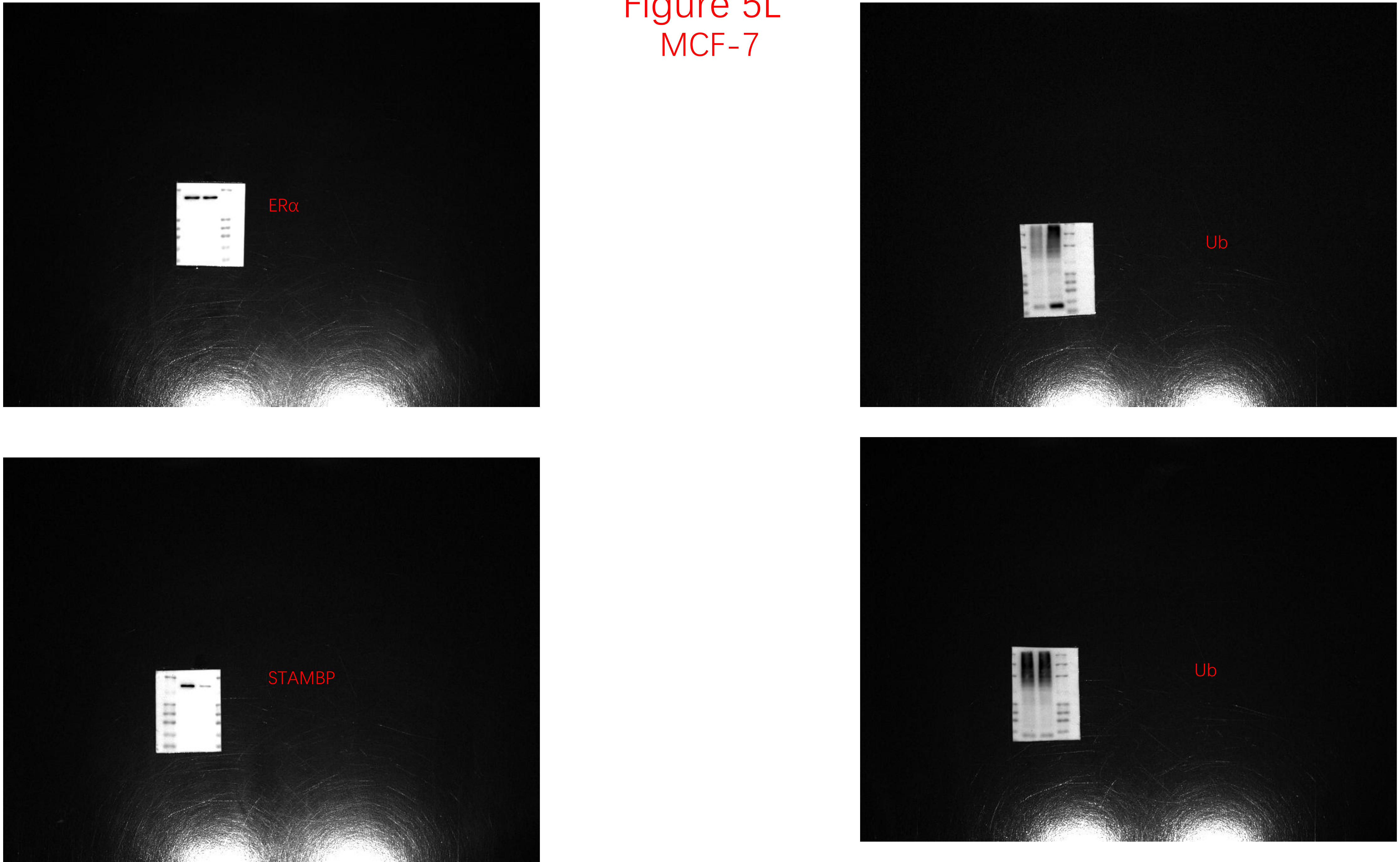

Figure 5L T47D

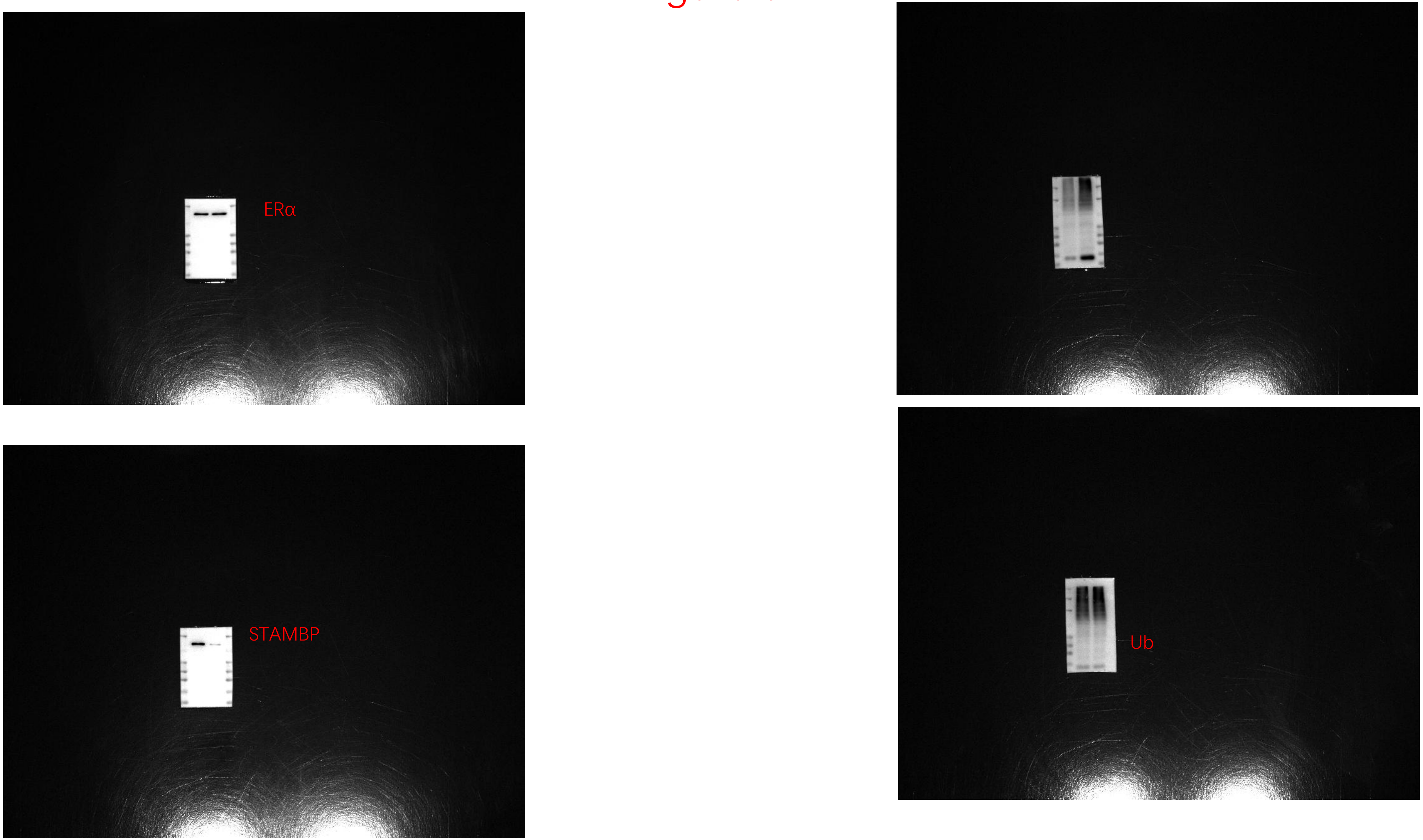

Figure 5M

MCF-7

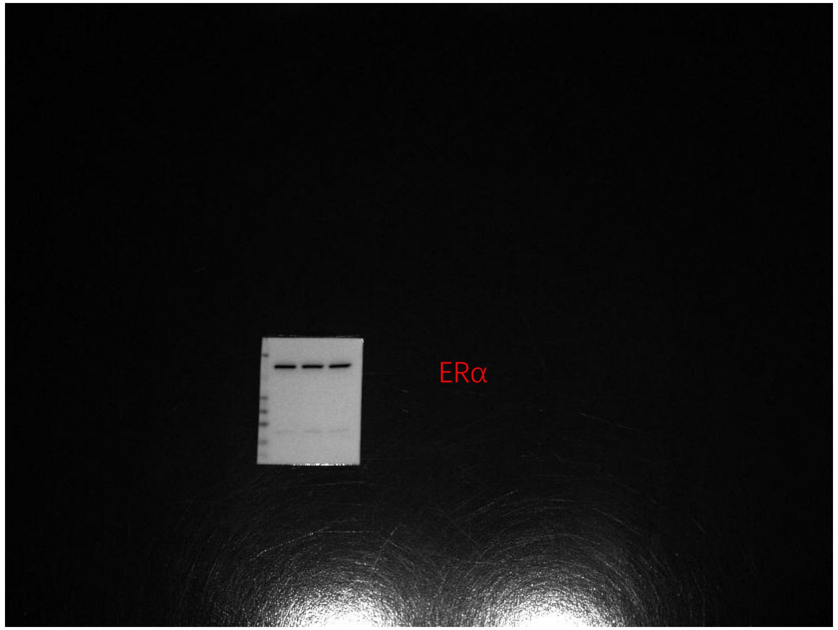

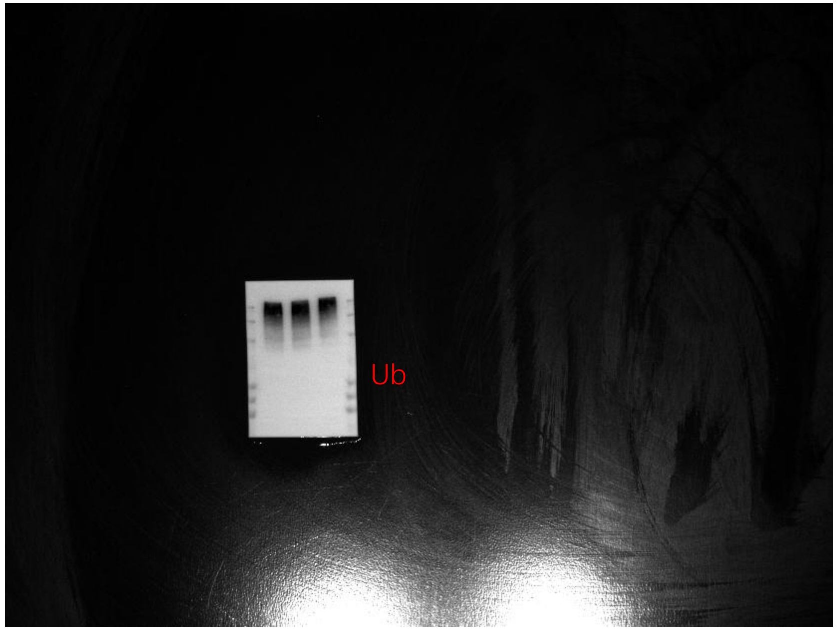

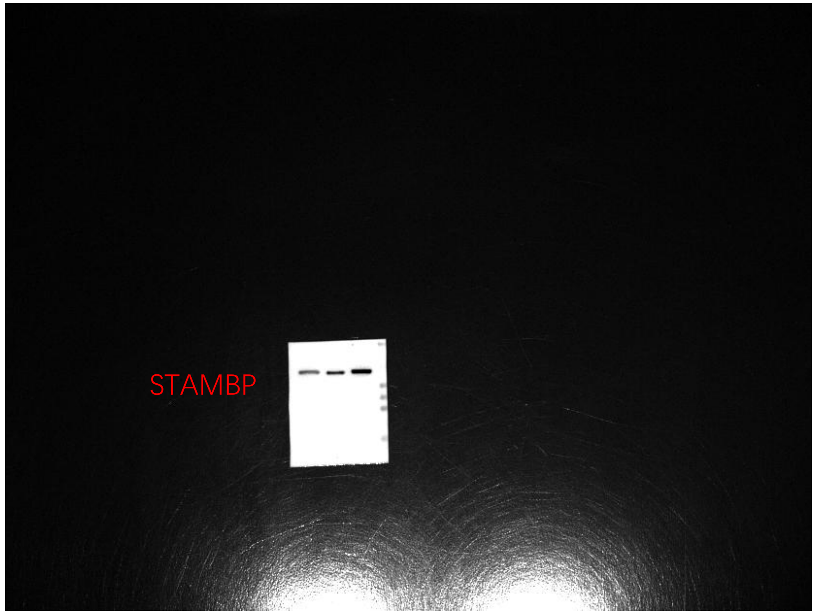

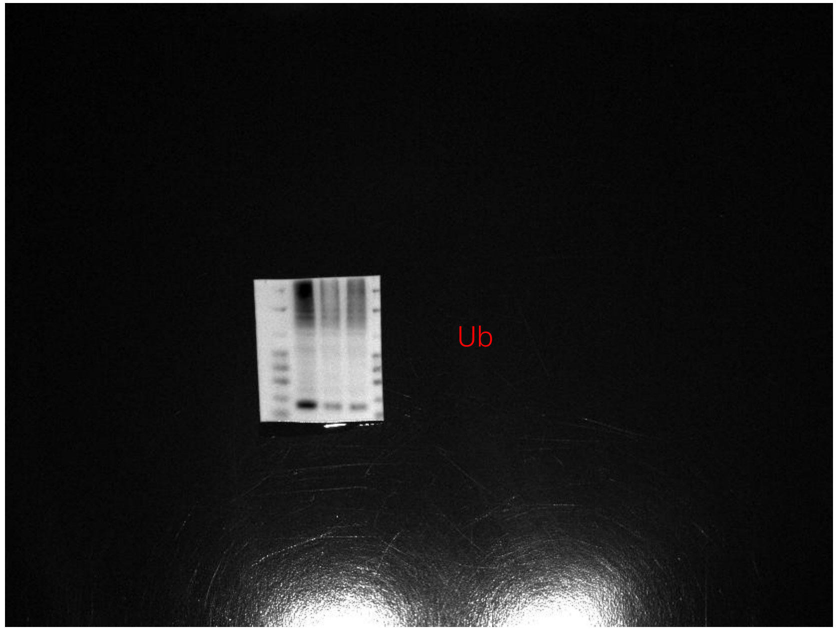

Figure 5M
T47D

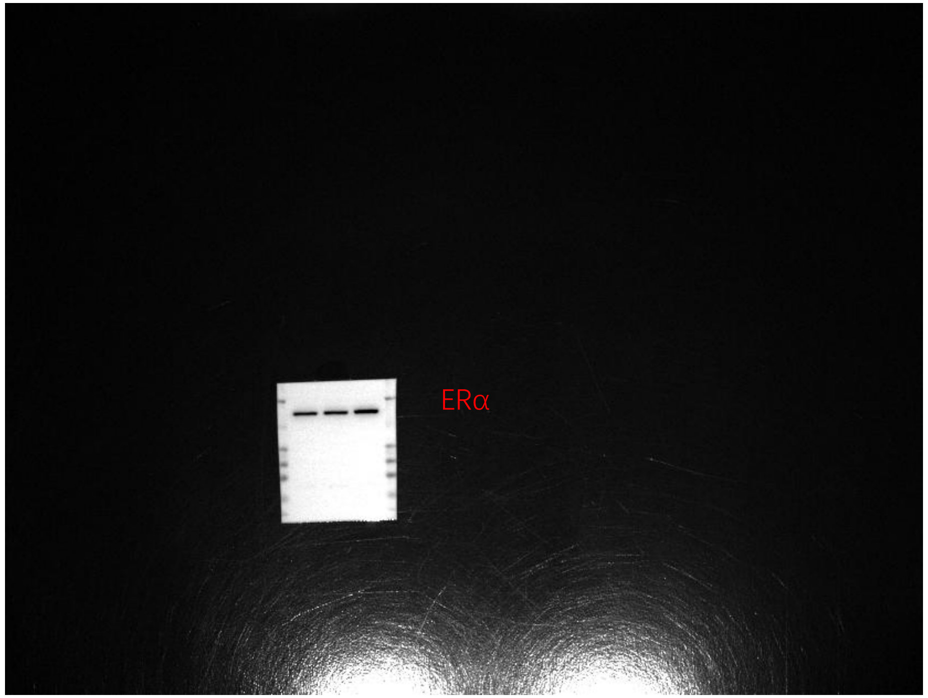

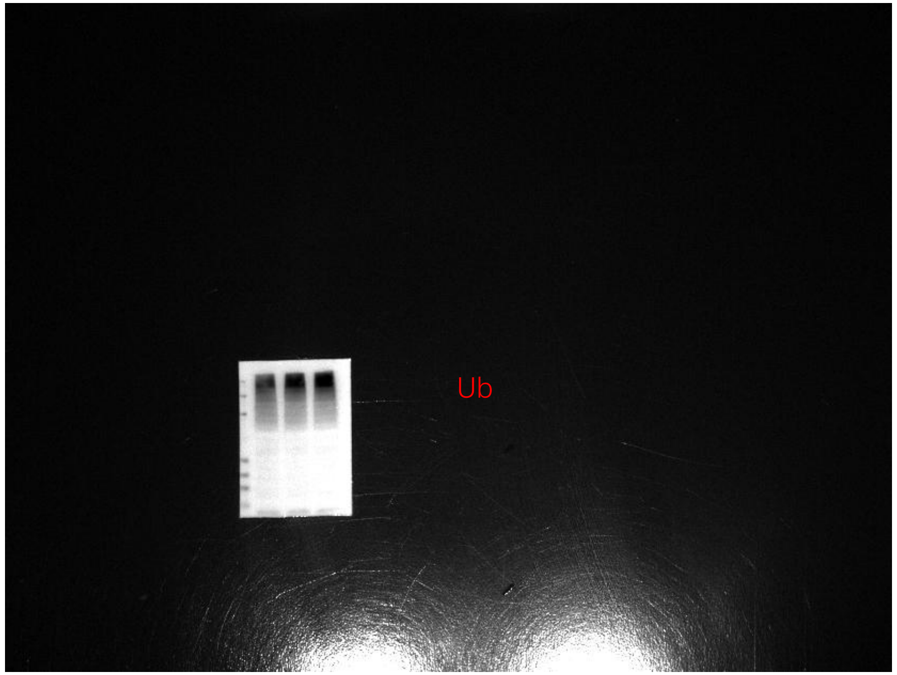

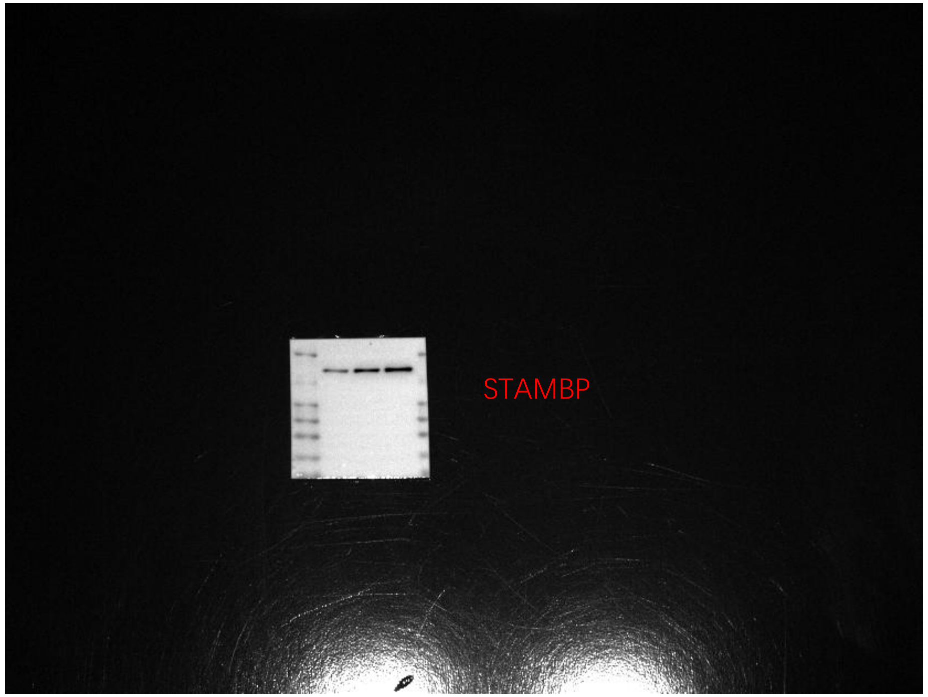

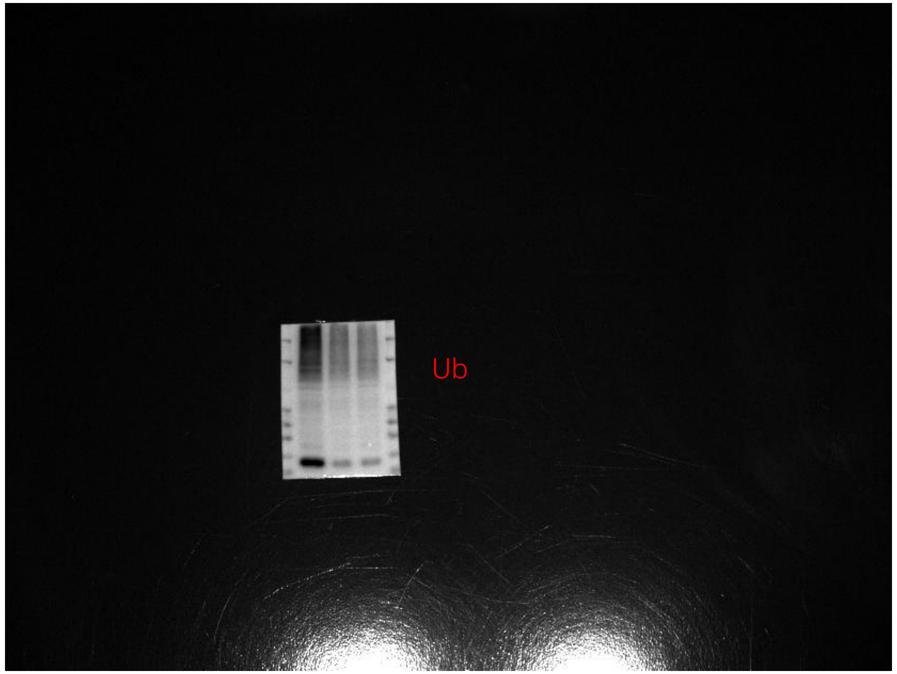

Figure 5N

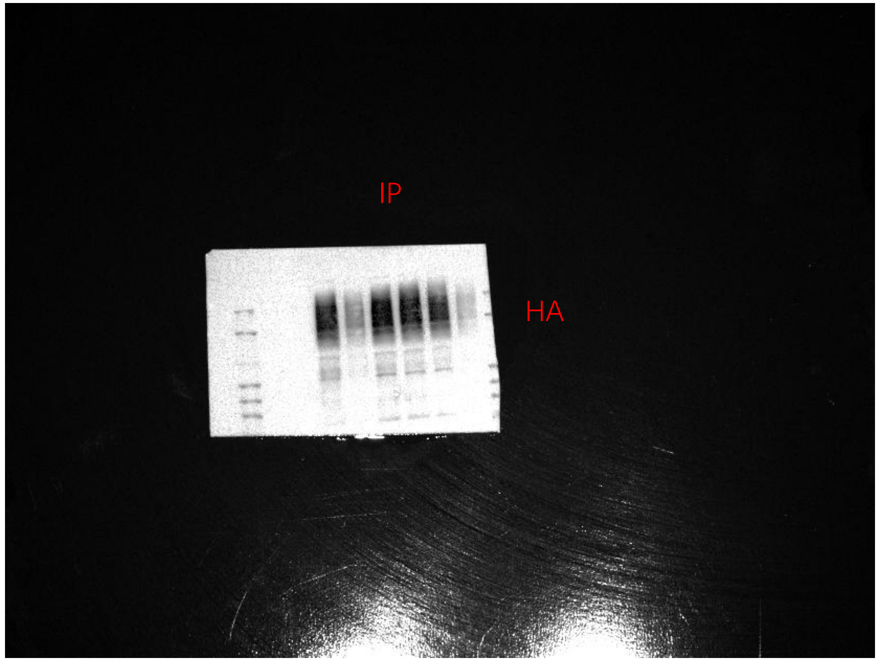

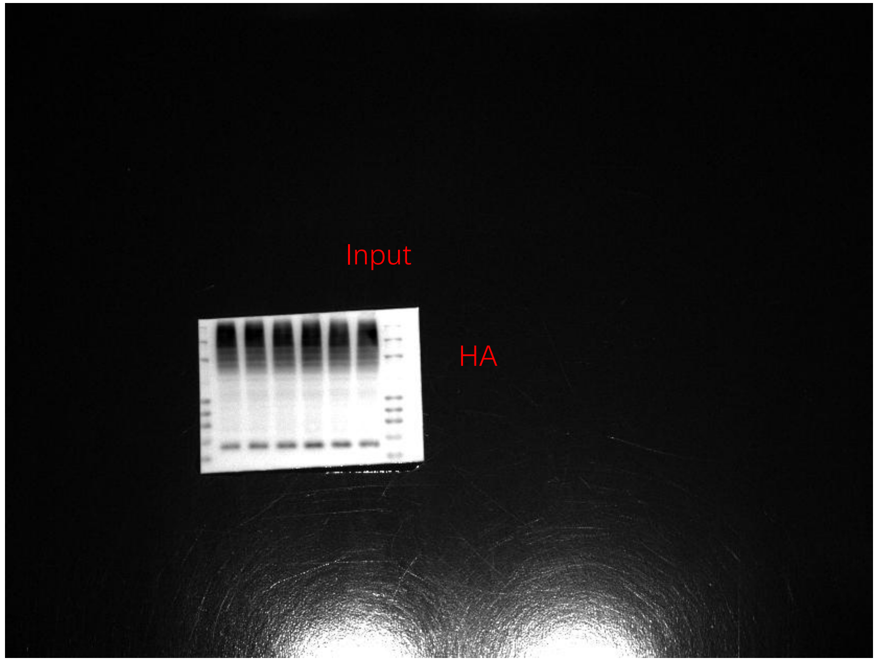

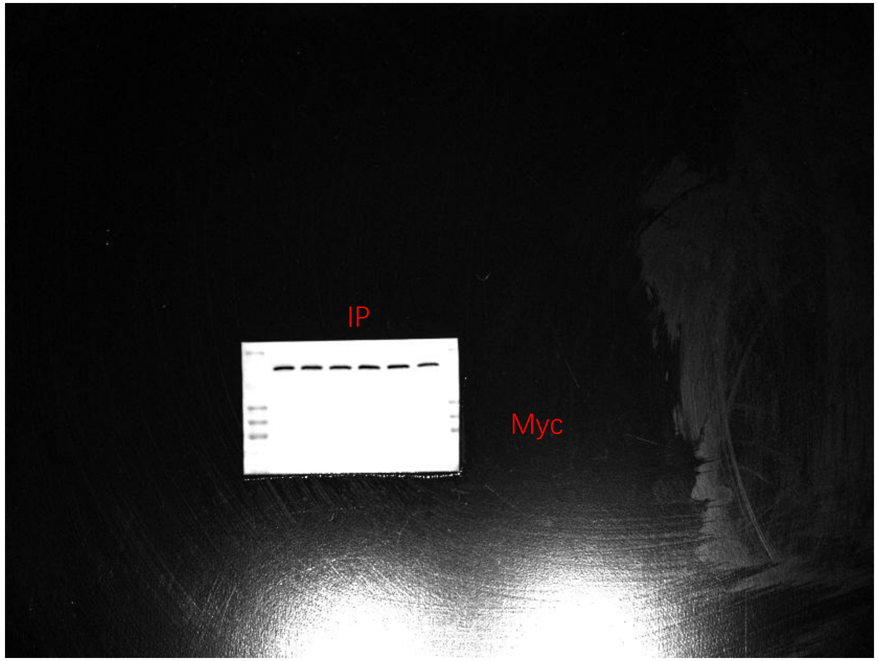

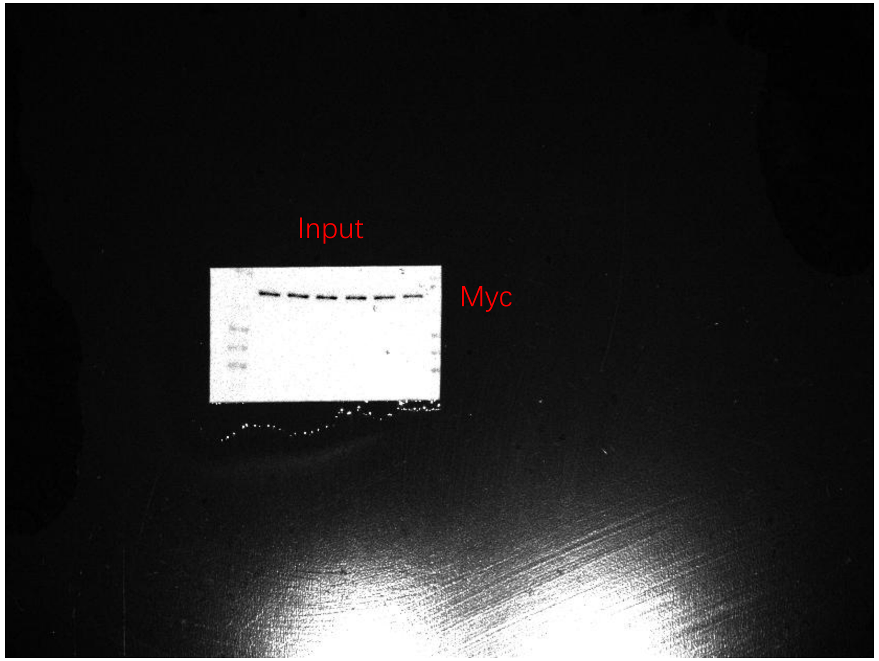

Figure 6B

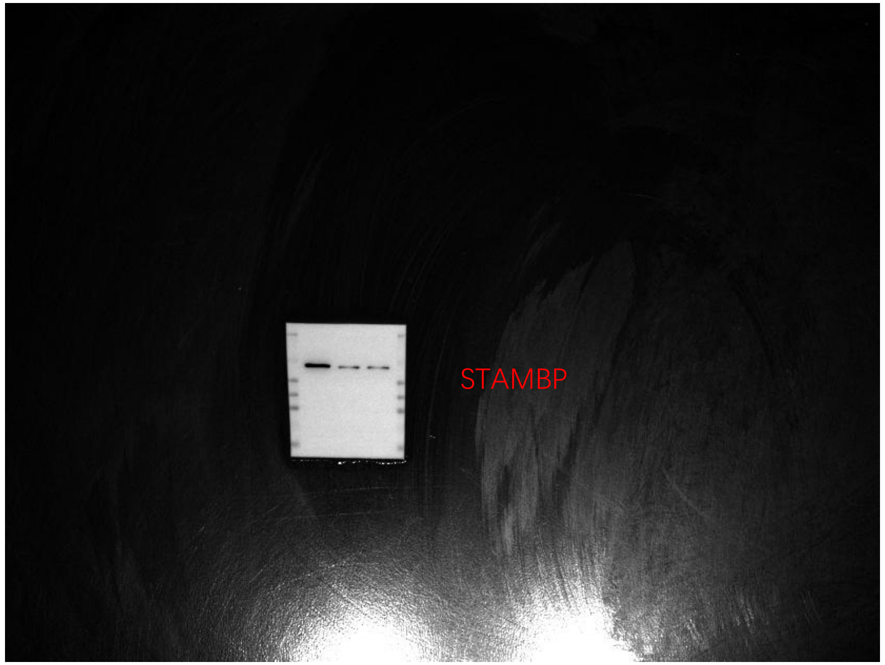

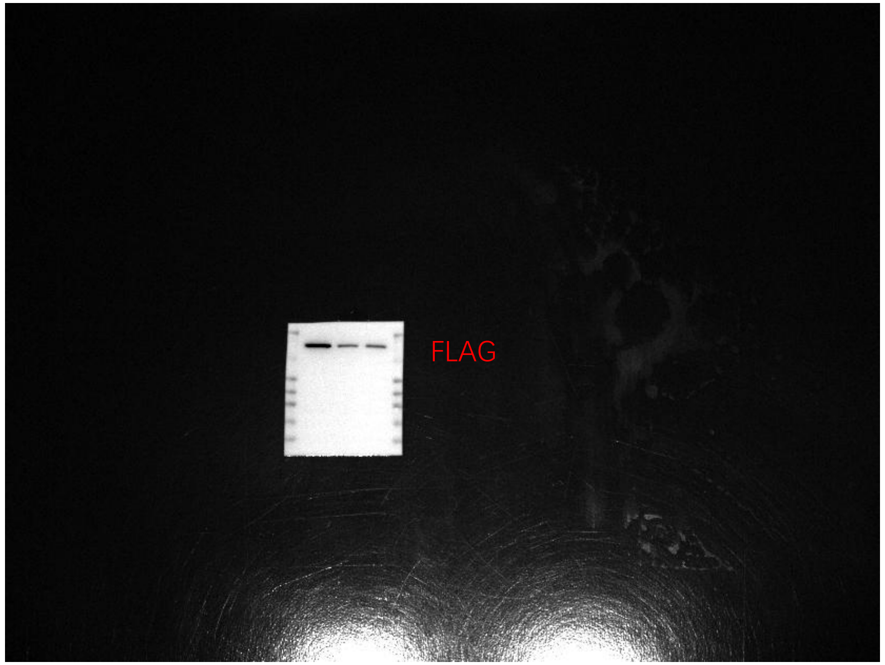

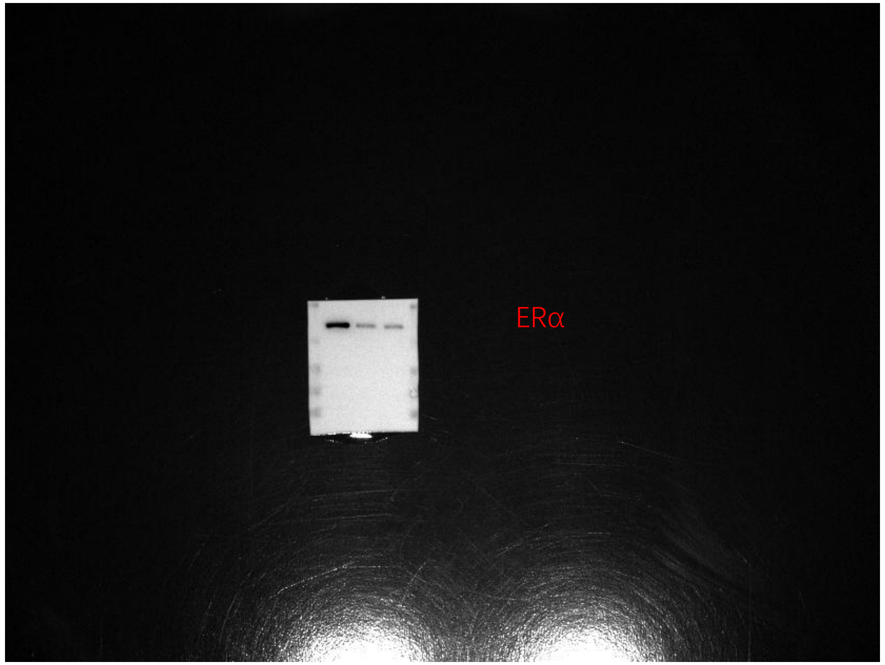

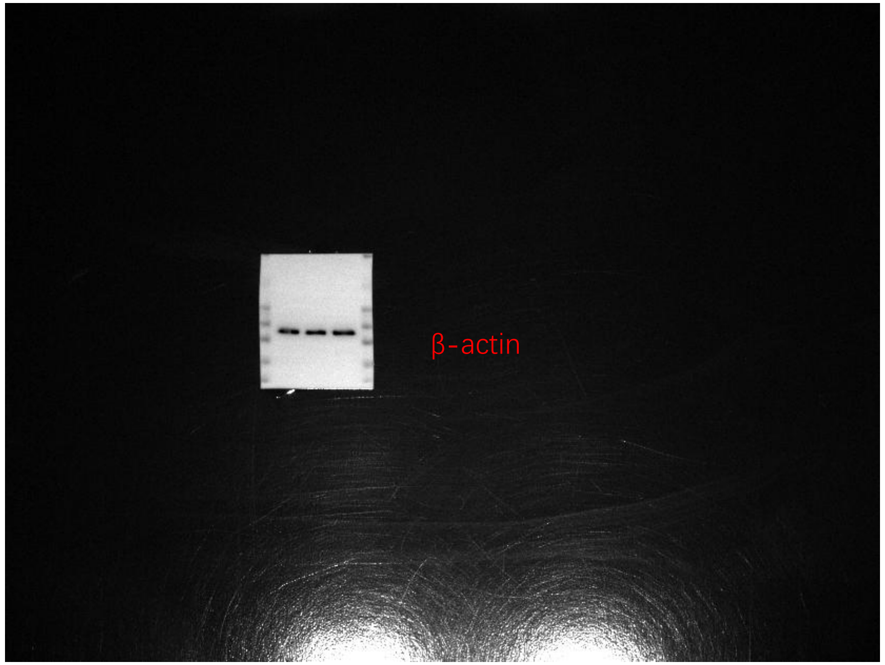

Figure S3

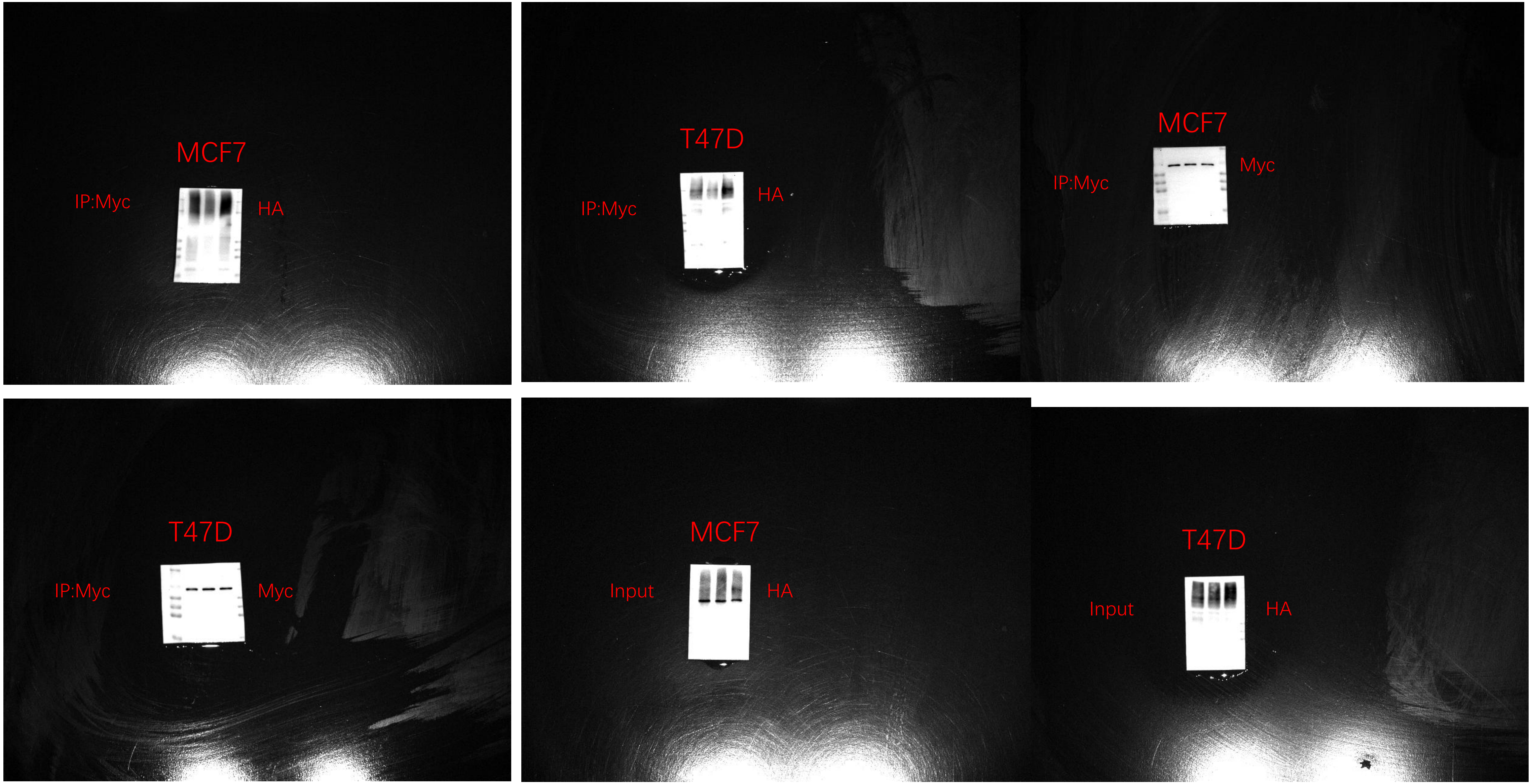
MCF7
IP:Myc
HA
T47D
IP:Myc
HA
MCF7
IP:Myc
Myc
T47D
IP:Myc
Myc
MCF7
Input
HA
T47D
Input
HA

Figure S3

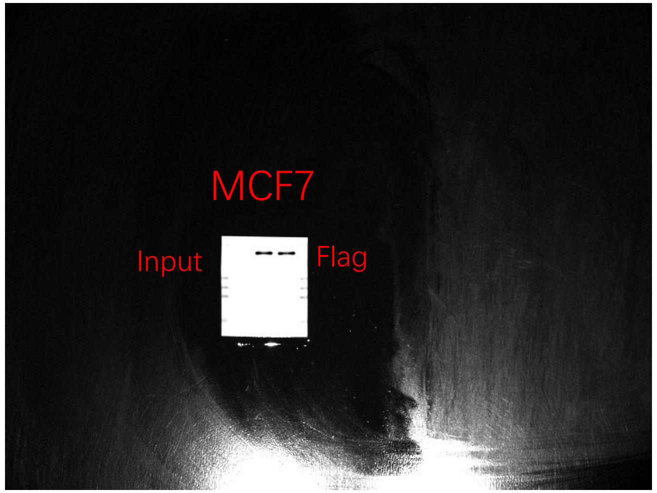
MCF7
Input
Flag

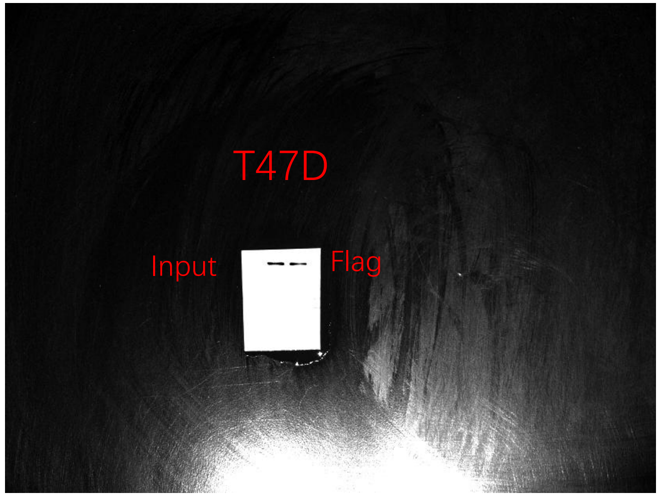
T47D
Input
Flag

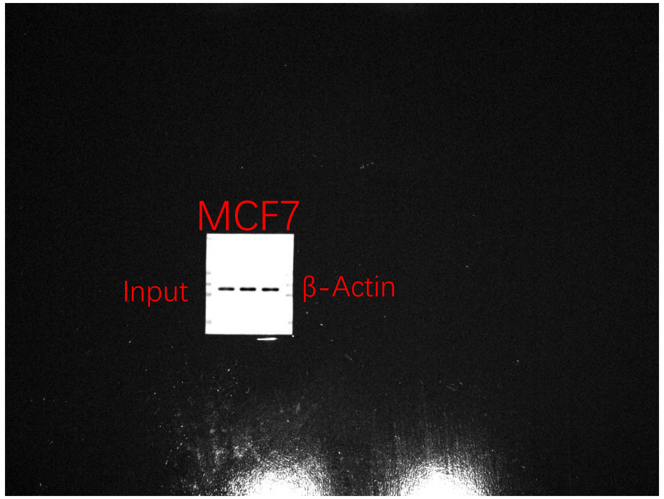
MCF7
Input
β-Actin

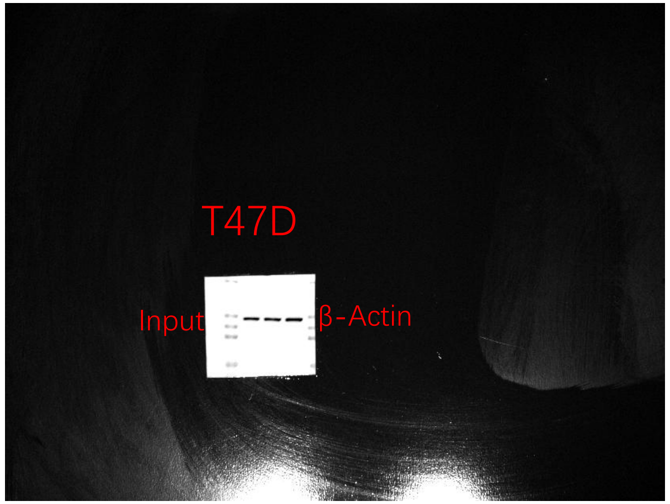
T47D
Input
β-Actin

Figure S5

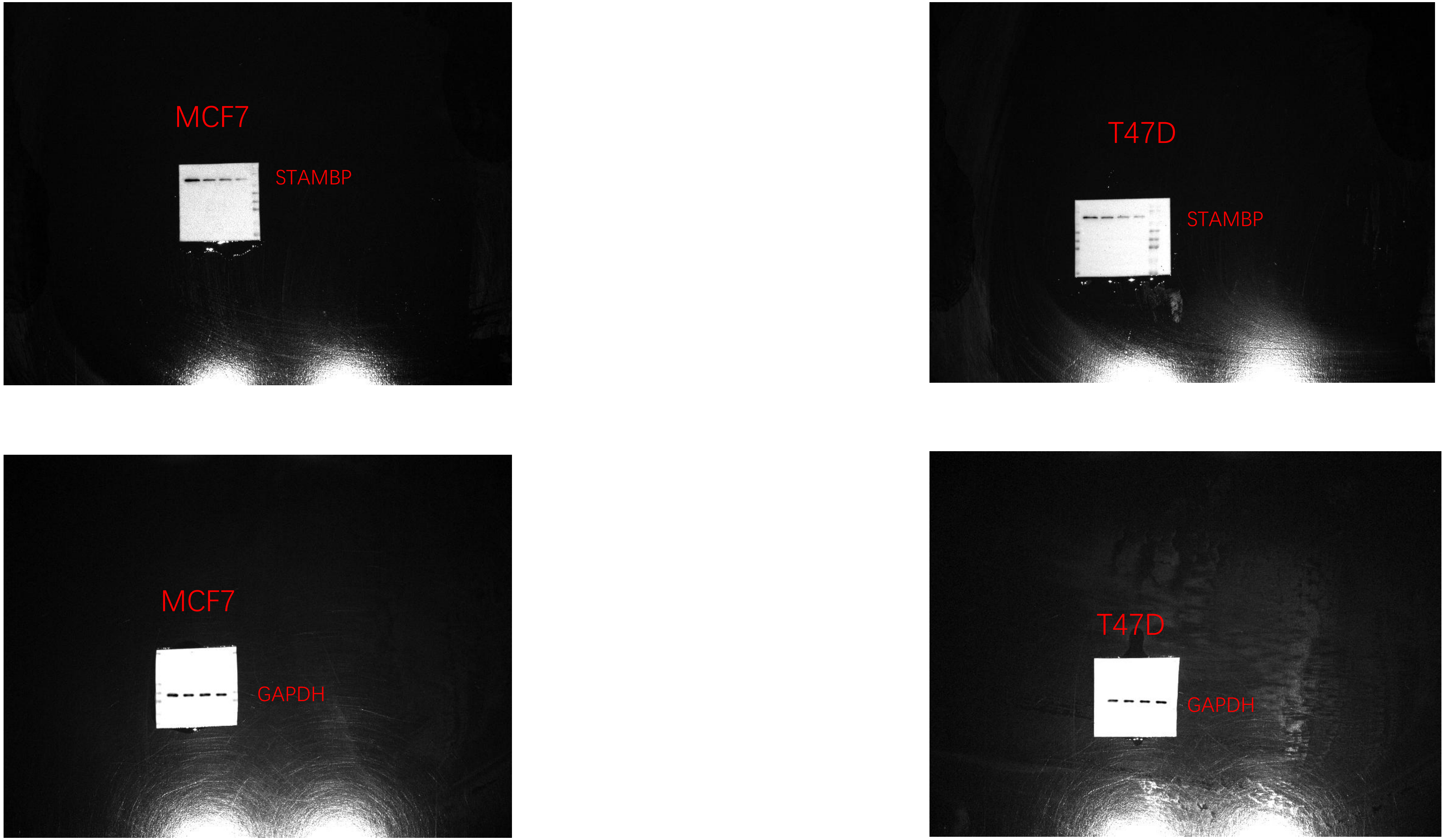

Supplement: Supplementary file 1 [file biomolecules-15-01502-s001.zip › Original images for blots.pdf]
