# Supplementary material for: STAMBP Accelerates Progression and Tamoxifen Resistance of Breast Cancer Through Deubiquitinating ERα
Source: Biomolecules. 2025 Oct 24;15(11):1502. doi: 10.3390/biom15111502 (PMC12650272; doi:10.3390/biom15111502)
Supplement: Supplementary file 1 [file biomolecules-15-01502-s001.zip › supplementary tables.pdf]

Supplementary Table S1 List of primer sequences

Gene	Forward primer sequence	Reverse primer sequence
β-actin	CATGTACGTTGCTATCCA GGC	CTCCTTAATGTCACGCA CGAT
STAMBP	CGGTAGAGGTGAATGAA GACAT	TGTTGCCTTCCTCAGAG TAAAT
ERα	GTGCTCTTTTTCCAGGTG GC	CAATGGTGCACTGGTTG GTG

Supplementary Table S2 List of transfection sequences

Gene	Transfection sequences
shSTAMBP-1	GCTGCTACTCTAAGTGCTGTT
shSTAMBP-2	GCTTCCTAACCATCGAGATTA
shSTAMBP-3	GCTTGAGGTTTCTGCTTGTAA

Supplementary Table S3 List of primary and secondary antibodies

Antibody name	Corporation	Application
Mouse anti-human GAPDH	Proteintech	WB:1/20000
Mouse anti-human β-actin	Proteintech	WB:1/10000
Rabbit anti-human STAMBP	Proteintech	WB:1/1000<br>IHC:1/200<br>IF:1/200
Mouse anti-FLAG	Cell Signaling Technology	WB:1/1000<br>IP:1/50
Mouse anti-human Ubiquitin	Santa	WB:1/200
Alexa Fluor 488 donkey anti-mouse IgG	Thermo Fisher Technology	IF:1/500
Alexa Fluor 555 donkey anti-rabbit IgG	Thermo Fisher Technology	IF:1/1000
HRP-labeled goat anti-rabbit IgG	Cell Signaling Technology	IF:1/1000
HRP-labeled goat anti-mouse IgG	Cell Signaling Technology	WB:1/2500
Normal Mouse IgG	Cell Signaling Technology	IP:1/50

Supplementary Table S4 List of DUB genes that regulate GREB1 expression in T47D cells

**Relative GREB1 expression**	**DUB gene**
0.291	STAMBP
0.311	BRCC3
0.335	USP39
0.352	PSMD14
0.367	USP36
0.382	UCHL5
0.389	FBXO7
0.423	OTUD5
0.425	UBL5
0.442	USP28
0.443	USPL1
0.456	USP45
0.476	BAP1
0.477	OTUB1
0.478	JOSD1
0.486	SENP2
0.497	USP16
0.499	USP49

Supplementary Table S5 Cox regression analyses of STAMBP in BRCA patients

Characteristics	Total(N)	Univariate analysis		Multivariate analysis	
		Hazard ratio (95% CI)	P value	Hazard ratio (95% CI)	P value
Pathologic T stage	1,083				
T1	277	Reference		Reference	
T2	631	1.334 (0.889 - 2.003)	0.164	0.924 (0.508 - 1.681)	0.796
T3&T4	175	1.931 (1.208 - 3.088)	**0.006**	2.592 (1.235 - 5.441)	**0.012**
Pathologic N stage	1,067				
N0	516	Reference		Reference	
N1	358	1.947 (1.322 - 2.865)	**< 0.001**	1.519 (0.859 - 2.685)	0.150
N2	116	2.522 (1.484 - 4.287)	**< 0.001**	1.897 (0.868 - 4.143)	0.108
N3	77	4.191 (2.318 - 7.580)	**< 0.001**	3.633 (1.488 - 8.871)	**0.005**
Pathologic M stage	925				
M0	905	Reference		Reference	
M1	20	4.266 (2.474 - 7.354)	**< 0.001**	3.445 (1.374 - 8.636)	**0.008**
Race	996				
Asian	60	Reference			
Black or African American	181	1.524 (0.463 - 5.020)	0.489		
White	755	1.320 (0.418 - 4.170)	0.636		
Age	1,086				
<= 60	603	Reference		Reference	
> 60	483	2.024 (1.468 - 2.790)	**< 0.001**	3.216 (1.945 - 5.318)	**< 0.001**
PR status	1,033				
Negative	342	Reference		Reference	
Positive	691	0.729 (0.521 - 1.019)	0.065	1.034 (0.472 - 2.266)	0.933

Characteristics	Total(N)	Univariate analysis		Multivariate analysis	
		Hazard ratio (95% CI)	P value	Hazard ratio (95% CI)	P value
ER status	1,036				
Negative	240	Reference		Reference	
Positive	796	0.709 (0.493 - 1.019)	0.063	0.371 (0.159 - 0.862)	**0.021**
HER2 status	717				
Negative	560	Reference		Reference	
Positive	157	1.593 (0.973 - 2.609)	0.064	1.036 (0.582 - 1.842)	0.905
STAMBP	1,086				
Low	542	Reference		Reference	
High	544	1.402 (1.015 - 1.936)	**0.040**	1.903 (1.149 - 3.153)	**0.012**
