# Supplementary figures and images for: STAMBP Accelerates Progression and Tamoxifen Resistance of Breast Cancer Through Deubiquitinating ERα
Source: Biomolecules. 2025 Oct 24;15(11):1502. doi: 10.3390/biom15111502 (PMC12650272; doi:10.3390/biom15111502)

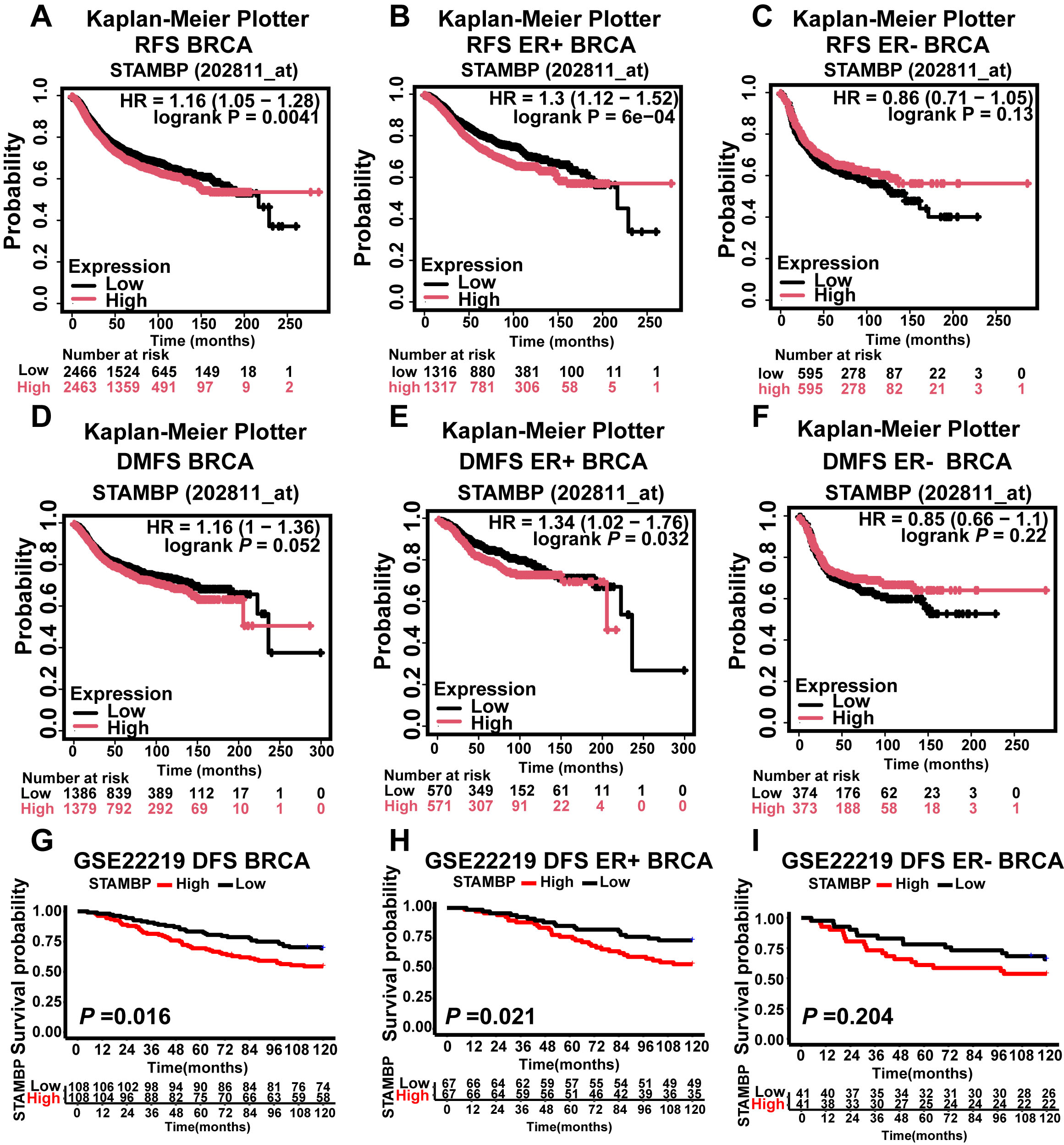

Supplement: Supplementary file 1 [file biomolecules-15-01502-s001.zip › Figure S1.jpg]

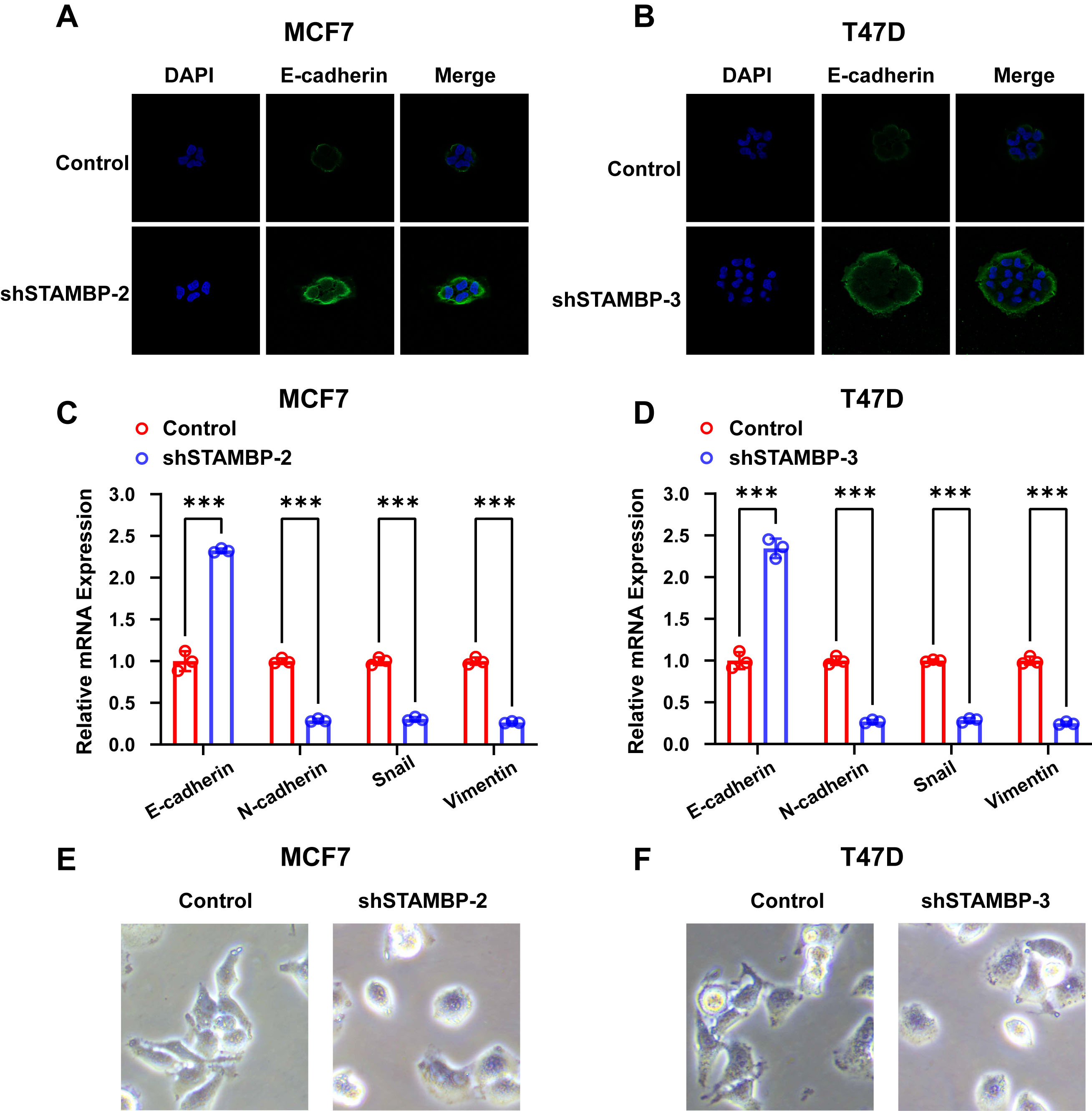

Supplement: Supplementary file 1 [file biomolecules-15-01502-s001.zip › Figure S2.jpg]

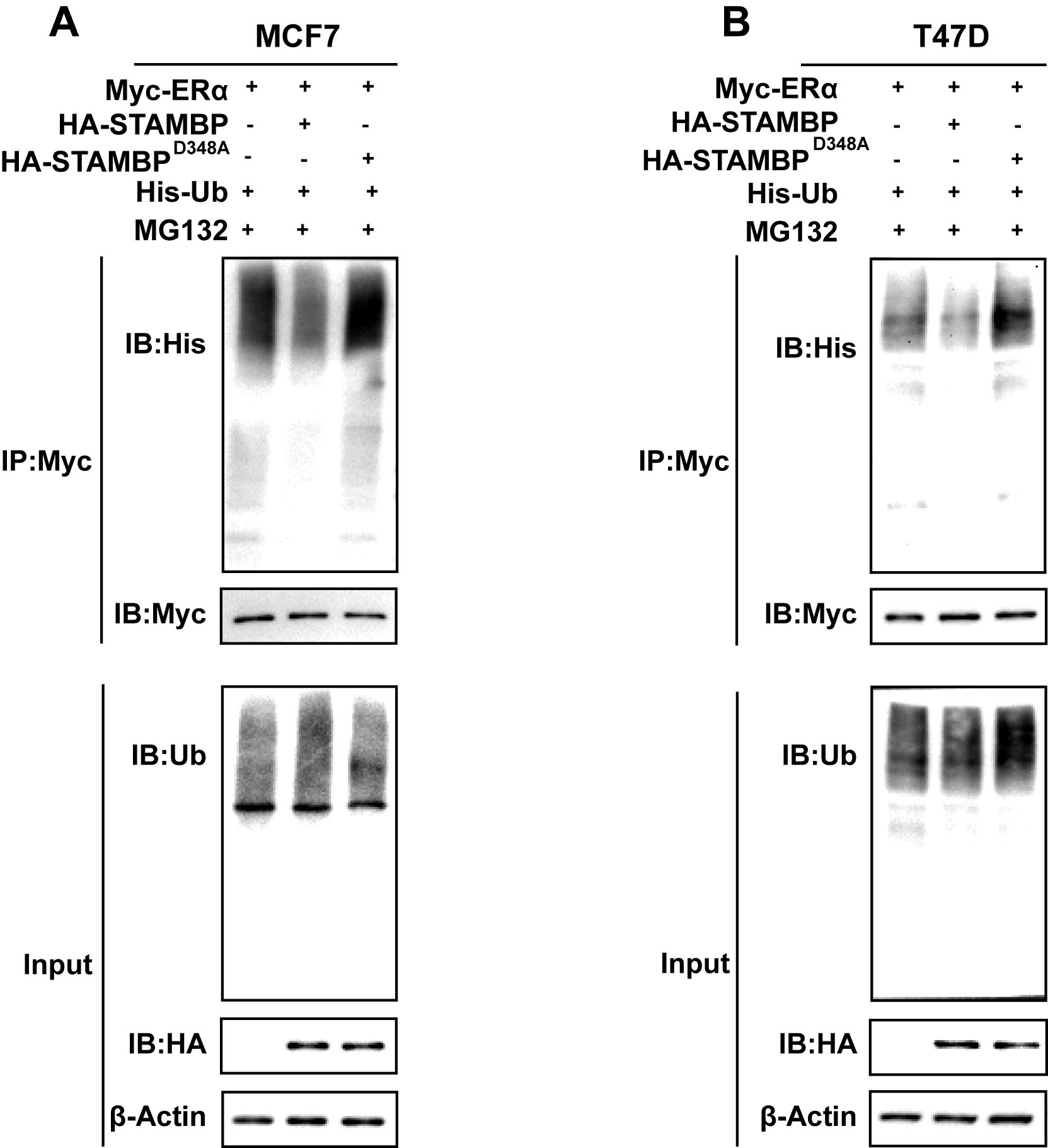

Supplement: Supplementary file 1 [file biomolecules-15-01502-s001.zip › figure S3.jpg]

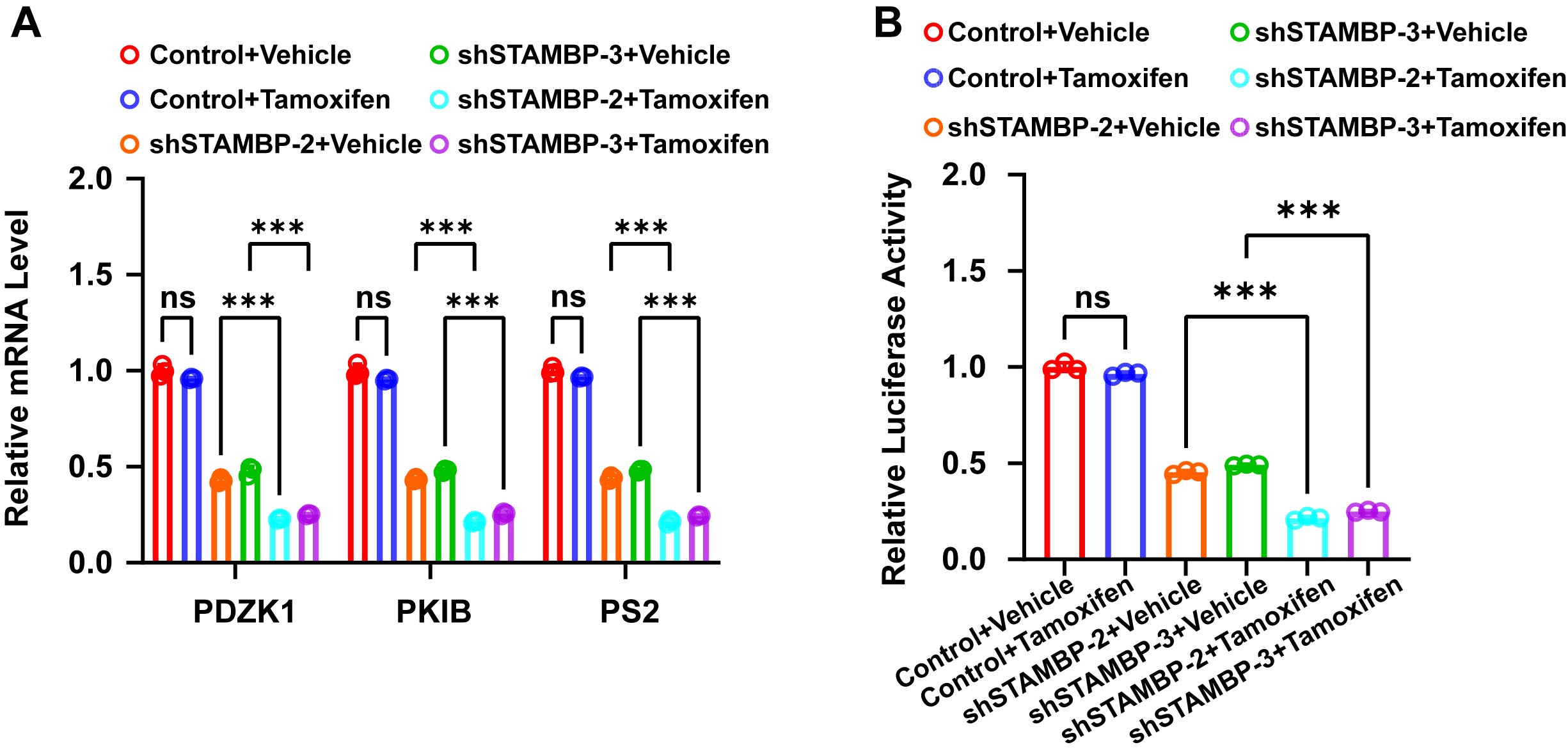

Supplement: Supplementary file 1 [file biomolecules-15-01502-s001.zip › Figure S4.jpg]

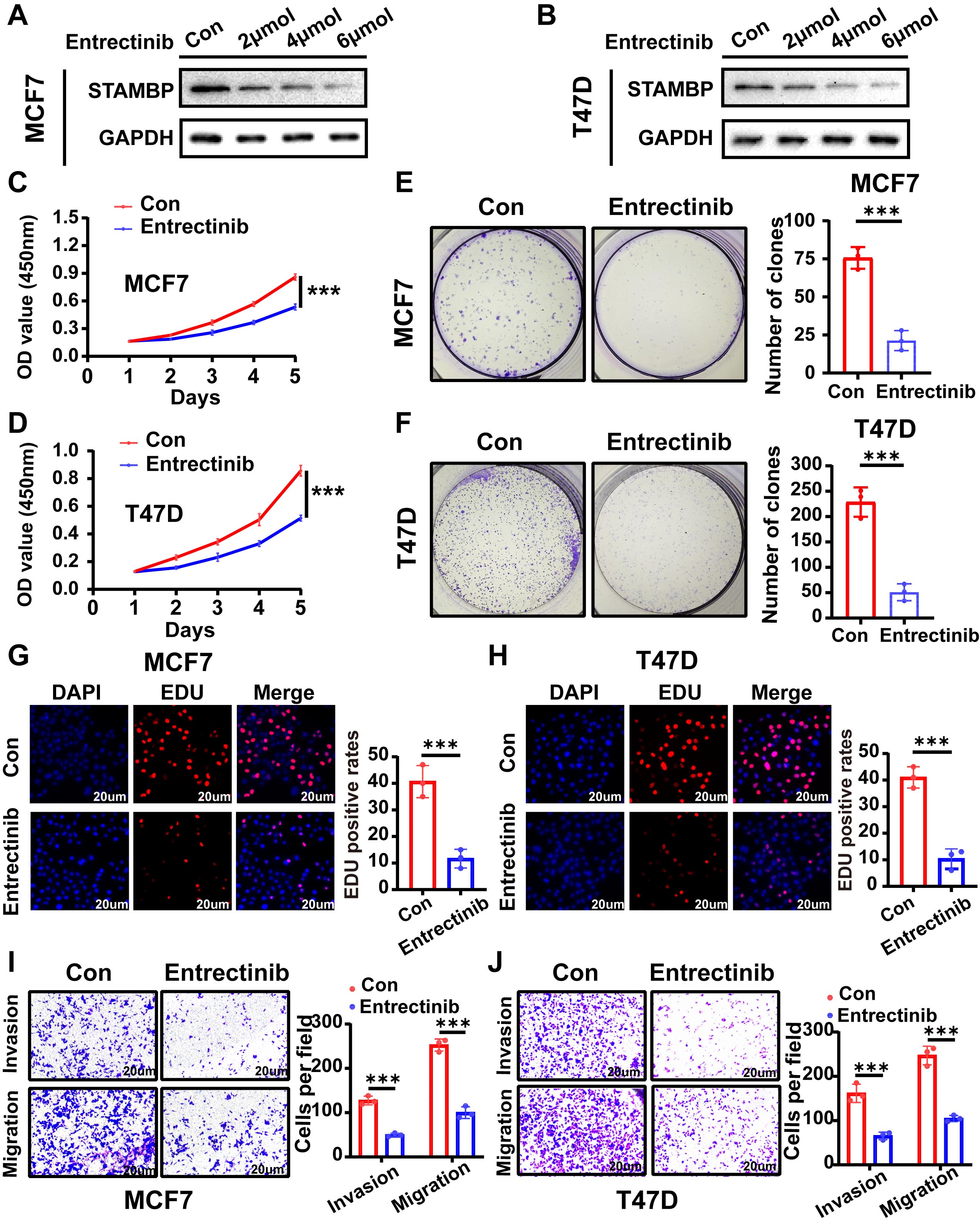

Supplement: Supplementary file 1 [file biomolecules-15-01502-s001.zip › figure S5.jpg]
